# Thermally activated delayed fluorescence in neutral and cationic stages of aza-nanographene decorated with 6.6.6 defects

**DOI:** 10.1039/d6sc06038b

**Published:** 2026-10-09

**Authors:** Rohith Valavil Suku, Illia E. Serdiuk, Krzysztof Dzieszkowski, Łukasz Orzeł, Monika Kijewska, Tadeusz M. Muzioł, Miłosz Siczek, Miłosz Pawlicki

**Affiliations:** a Faculty of Chemistry, Jagiellonian University Gronostajowa 2 30-387 Kraków Poland pawlicki@chemia.uj.edu.pl; b Jagiellonian University, Doctoral School of Exact and Natural Sciences Prof. St. Łojasiewicza 11 Kraków Poland; c Faculty of Mathematics, Physics and Informatics, University of Gdansk ul. Wita Stwosza 57 80-308 Gdańsk Poland illia.serdiuk@ug.edu.pl; d Department of Chemistry, University of Wrocław F. Joliot-Curie 14 50383 Wrocław Poland; e Department of Chemistry, UMK Gagarina 7 87-100 Toruń Poland

## Abstract

Heteroatom-doped polycyclic aromatic hydrocarbons (PAHs) show substantially modified electron distribution caused by the presence of either an electron-rich or electron-poor element that significantly influences the optical properties of final molecules. A novel synthetic approach with a PBr_3_ coupling agent, applied to precisely designed monomers and followed by intramolecular fusion, incorporates a tetraaza-dibenzo[*g*,*p*]chrysene decorated with two 6.6.6 defects into a nanographene-like system. The final derivative shows an efficient green fluorescence emission (*λ*_PF_ = 530 nm) accompanied by phosphorescence (*λ*_phos_ = 565 nm). The emission is tuned with simple protonation and shifted bathochromically to *λ*_PF_ = 620 nm and *λ*_phos_ = 640 nm. Both forms show thermally activated delayed fluorescence (TADF) with PLQYs of 93% for the green band and 50% for the red one, showing a unique ability to sustain the TADF behaviour after protonation.

## Abstract 2

N-doped nanogaphene-like system decorated with 6.6.6 defects exhibits effective green TADF in neutral form. Its unique optical features allows sustaining the TADF character for bathochromically shifted emission recorded in a cationic mode.

## Graphical abstract

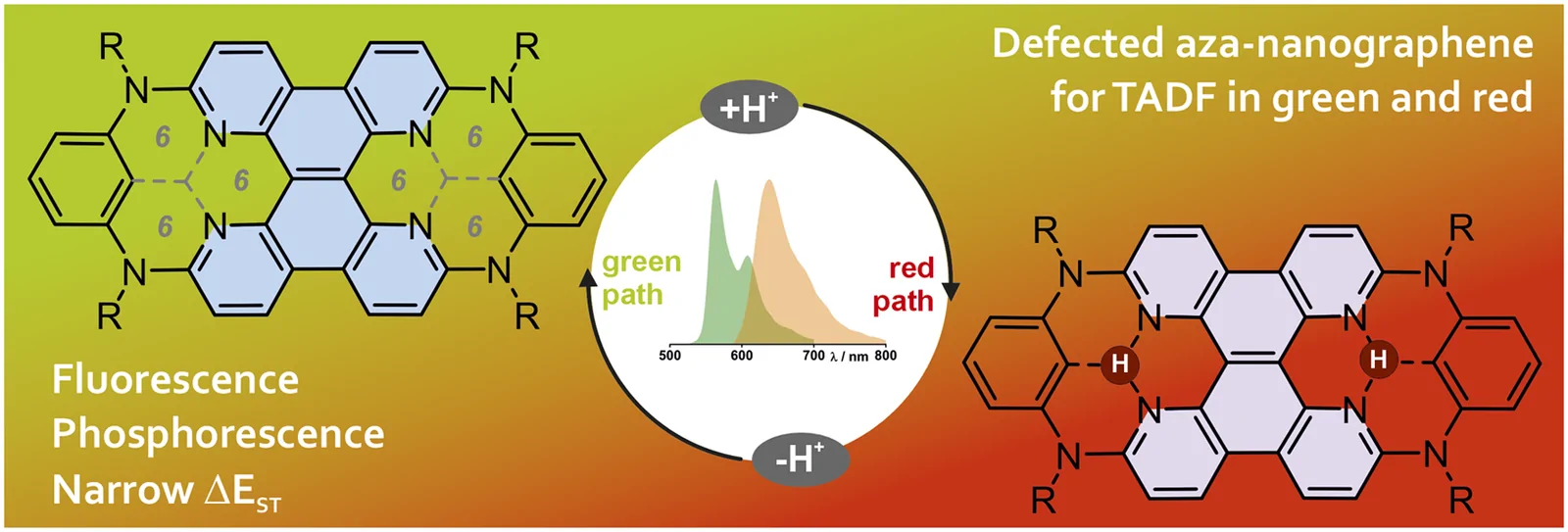

## Introduction

Structural modification of π-extended derivatives by incorporation of heteroatoms deeply modulates the optical response depending on the quality of the involved element. Polycyclic aromatic hydrocarbons (PAHs) are continuously explored to pursue the fundamental properties of optical materials, focusing on modulation of molecular orbitals and changing the absorption and emission wavelength.^1^ Either an electron-rich (*e.g.*, amine nitrogen)^2^ or electron-poor (*e.g.*, boron)^3,4^ element drastically influences the optical response, introducing significant distortion of the even distribution of π-electrons. Nitrogen-containing PAHs, reported as powerful tools for tuning the optical response utilizable in OLEDs or OFETs, *etc.*,^5,6^ rely on a subtle balance between imine and amine character, which directly correlates with electron-accepting and electron-donating character that modifies the HOMO–LUMO gap,^2^ influencing the optical properties. The donor–acceptor constructs of unsaturated and π-conjugated systems involving nitrogen as part of the main motif show a modulated spin–orbit coupling and the singlet-triplet energy gap (SOC, Δ*E*_ST_)^7,8^ crucial for efficient thermally activated delayed fluorescence (TADF). The original contribution of Adachi and co-workers demonstrated in 2012 the thermally activated fluorescence in a D–A system (Fig. 1), occurring *via* a long-range charge transfer (LR-CT) that rebuilds the population of S_1_*via* rISC, requiring a small Δ*E*_ST_ observed as a consequence of physical separation of HOMOs/LUMOs.^9^ The TADF properties of donor–acceptor systems have been extensively explored, eventually forming a set of derivatives with a linear construction and a significantly marked dipole moment.

**Fig. 1:**
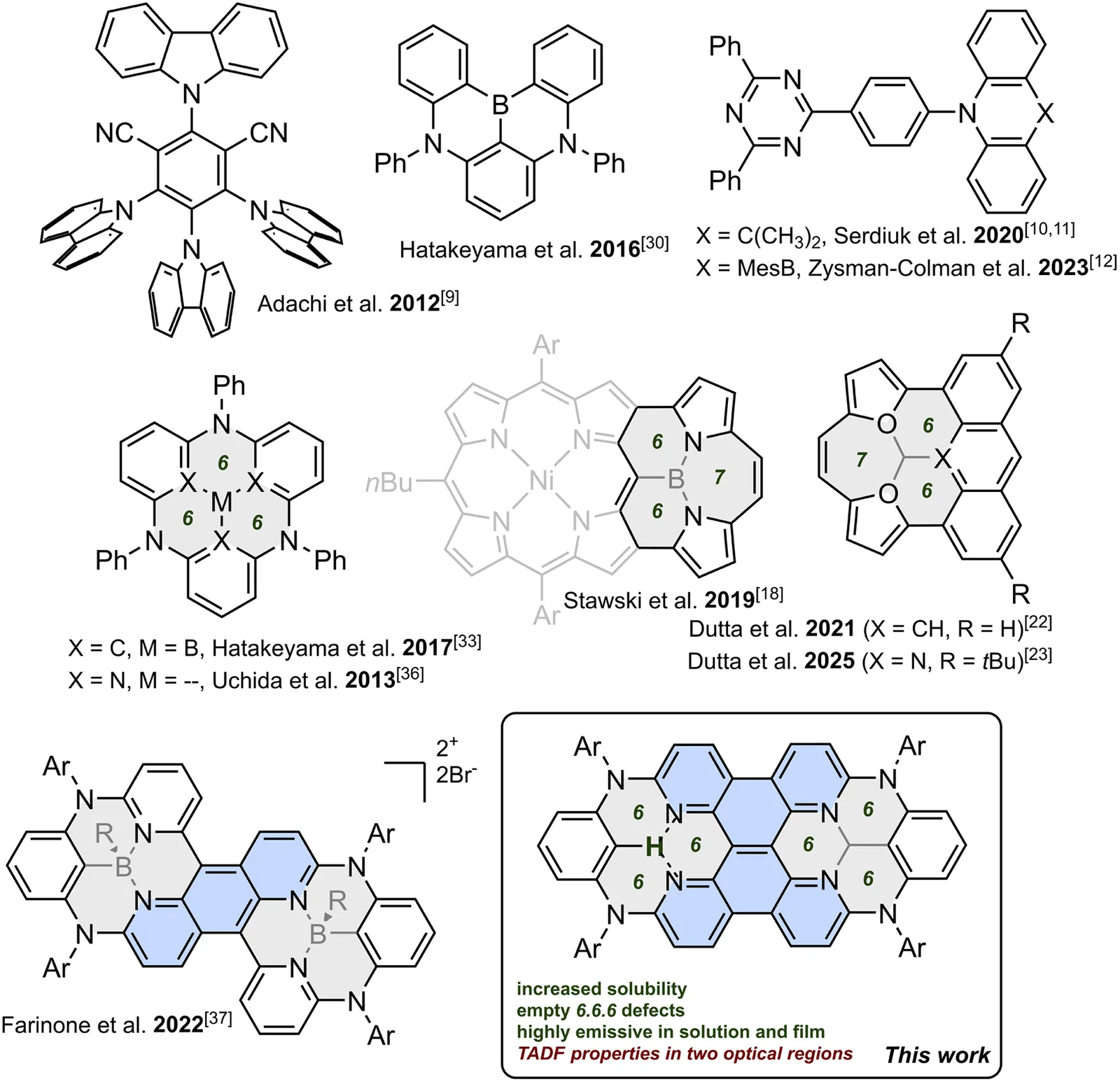
6.6.6 and 7.6.6 defects built on π-extended derivatives.

Tsai *et al.*,^10^ Serdiuk *et al.*^11,12^ and Zysman-Colman and co-workers^13^ reported a significant correlation between the dihedral angle observed between donor and acceptor subunits and registered (and theoretically anticipated) SOC/Δ*E*_ST_ parameters observed for D–A systems with the charge-transfer transition. The separation of the donor and acceptor in D–A systems sacrifices *k*_r_ and SOC to gain low Δ*E*_ST_.^10–12^ The magnitude of spin–orbit coupling (SOC) has usually been correlated with the presence of heavy atoms (*e.g.*, –Br),^13^ but fully organic units, *e.g.*, carbonyl, C=O, were also reported as elements improving the S/T spin–orbit coupling^14–17^ and directing design paths towards such skeletons.^5^ On the other hand, the presence of macrocyclic constraints opens several possibilities for controlling the delocalization activated by the molecule's dynamic^18,19^ or the redox impulse,^20,21^ also showing unorthodox conjugation paths, as can be found in our contributions (Fig. 1).^22–24^ The macrocyclic constraints allow forcing the formation of open-shell spin states with a dynamic process^25,26^ but also show a post-synthetic modification approach that drastically modifies the observed photophysical properties.^27,28^ Nevertheless, the TADF properties for the macrocyclic motif are rarely reported.^29^ Hatakeyama and co-workers reported an alternative mechanism of TADF, focusing on separation of frontier orbitals within a directly connected π-system with a donor (*e.g.*, N) or acceptor (*e.g.*, B), leading to short-range charge transfer (SR-CT) in the multi-resonance effect (MR-TADF). A precise construction of such systems enriched with the electron-poor character of boron(iii) in the 6.6.6 defect^4^ gave a narrow emission recorded in the blue^30–32^ and green regions.^33^ Till now, the TADF tests for defected systems lack post-synthetic modulation focusing on a fundamental change with a significant influence on the observed outcome. An additional and, to date, rarely observed TADF feature arises from the protolytic responsiveness of the chromophore. While protonation is a powerful way to increase acceptor strength and alter charge-transfer character, protonated/cationic TADF emitters remain extremely rare, as charged acceptors typically quench the emission completely. A few examples of acid/base-responsive TADF or delayed-emission systems have been reported, including pyridine-containing donor–acceptor TADF emitters,^34^ as well as protonation/deprotonation-controlled switching between TADF and room-temperature phosphorescence.^35^ Nevertheless, systems in which both the neutral and protonated molecular forms are emissive and display TADF-type behavior are still highly uncommon. Thus, decoration of a designed chromophore/fluorophore with a changeable motif, *e.g.*, macrocyclic, potentially introduces a controlling factor being able to change the observed outcome, in certain cases forcing/changing TADF properties of the final skeleton.^36^ The tetraaza-dibenzo[*g*,*p*]chrysene derivative reported here, containing two 6.6.6 defects, shows efficient green emission with a high PLQY of 93% in its neutral form, while protonation of the defect-containing framework gives a bathochromically shifted red-emissive form with a PLQY of 50%. Importantly, both forms show delayed fluorescence arising from rISC, demonstrating that protonation does not suppress TADF but instead creates a second, red-shifted TADF-active state. This neutral/protonated TADF pair represents a rare example of a protolytically switchable diazachrysene-based SR-CT emitter, in which simple post-synthetic protonation provides access to two efficient TADF-emissive states from a single molecular framework.

## Results and discussion

### Synthesis and characterization

The synthetic approach leading to the formation of 1 – the structural motif merging two 6.6.6 defects with an aza-analogue of dibenzo[*g*,*p*]chrysene – relies on the employment of precisely located carbonyl functionality in 2 (Scheme 1) and was obtained *via* the Buchwald–Hartwig reaction following the reported procedure (see the SI, Scheme S1).^21^ Its formation has been confirmed by scXRD analysis (Scheme 1, inset) and spectroscopic analysis (see the SI). In the next step, we activated the carbonyl unit with phosphorous tribromide, forming dimer 3 with 65% yield in a neutral form and empty 6.6.6 defects. The carbonyl unit in 2 had previously been activated towards dimerization with BBr_3_, leading to the expected reaction accompanied by boron(iii) binding, eventually resulting in a cationic molecule with significant problems with post-synthetic processability.^37,38^ Subsequent fusion performed for 3 with a single Mallory fusion (Scheme 1, path b) gave a semi-fused system 4 with 75% yield. Dimer 4 can be further fused in the next Mallory reaction (Scheme 1, path c), leading to the formation of 1; nevertheless, the efficiency of this transformation *ca.* 20% was not satisfactory. Alternatively, the same transformation could be achieved by Scholl-like fusion (Scheme 1, path d) that led to the desired product 1 with a yield of 96%.

**Scheme 1:**
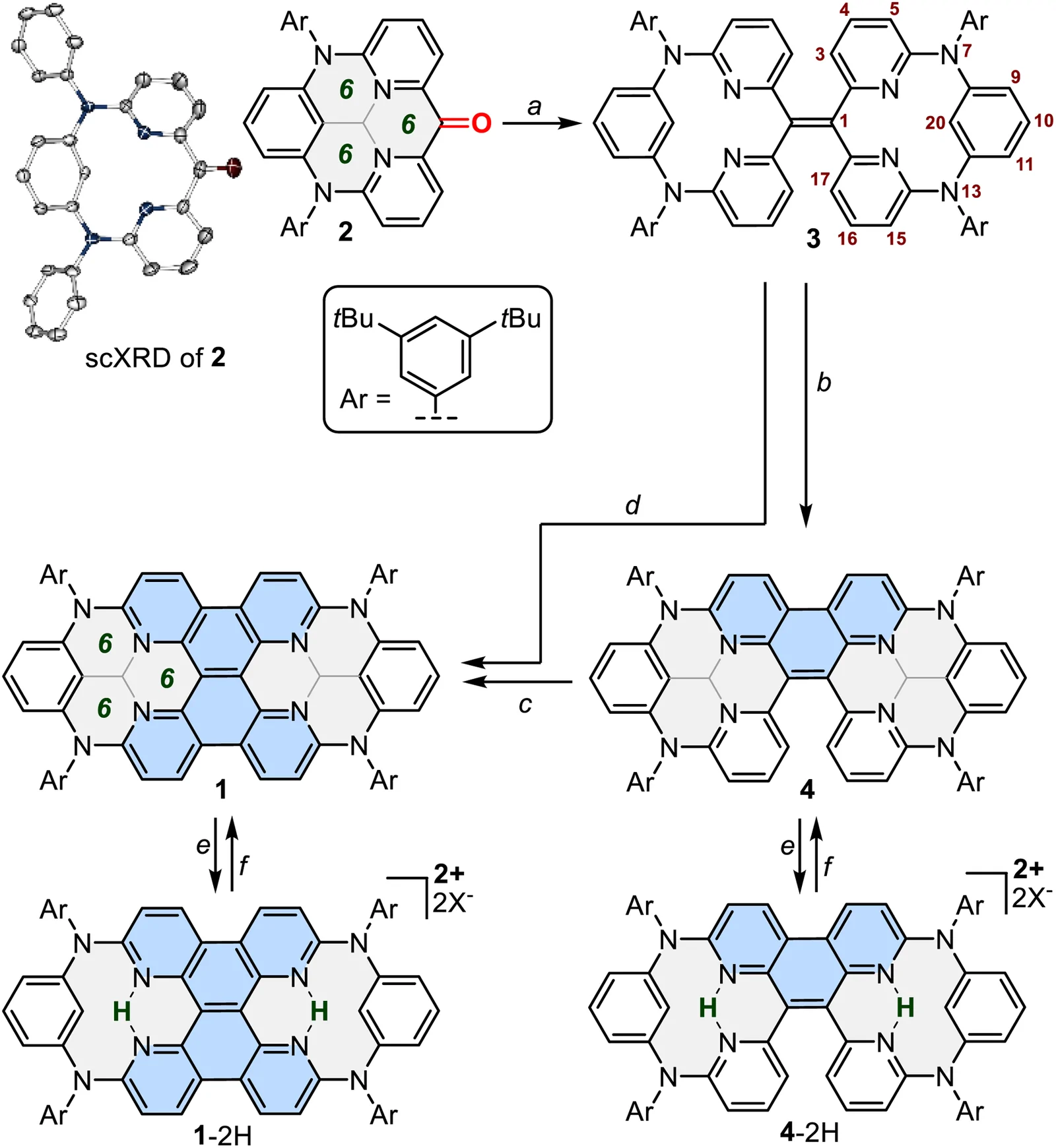
The applied synthetic approach. Conditions: (a) PBr_3_, *o*-dichlorobenzene, 180 °C, 14 h; (b) *hν* (370 nm), O_2_, EtOAc, 2 h; (c) *hν* (370 nm), O_2_, EtOAc, 14 h; (d) DDQ, MeSO_3_H, DCE, reflux, 24 h; (e) TFAH, CH(D)Cl_3_; (f) Et_3_N, CH(D)Cl_3_. The inset presents the sc-XRD structure of 2 (*t*-Bu substituents omitted for clarity; thermal ellipsoids represent 50% probability).

The HRMS experiments performed for 1, 3 and 4 showed the *m*/*z* signals at 647.4129 [M + 2H]^2+^ (1) (theor. calc. 647.4108 for C_90_H_100_N_8_ + 2H^+^), 1297.8384 [M + H]^+^ (3) (theor. calc. 1297.8462 for C_90_H_104_N_8_ + H^+^), and 1295.8213 [M + H]^+^ (4) (theor. calc. 1295.8300 for C_90_H_102_N_8_ + H^+^), consistent with the expected compositions and assigned to the anticipated skeleton. The ^1^H NMR data consistently supported the expected skeletons, with the spectroscopic picture observed for locally conjugated hydrocarbons.^39^ The gradual fusion in the series 3 → 4 → 1 influences the ^1^H NMR spectra (Fig. 2). The most noticeable difference observed was assigned to the change in the chemical shift of the resonance assigned to H(20) that gradually shifts to higher values of chemical shifts (*δ* = 7.67 ppm → *δ* = 8.79 ppm → *δ* = 10.00 ppm) confirming the increased efficiency of the de-shielding effect. The edge position present on phenanthroline units (H(4,16)) appears after the first fusion at *δ* = 8.30 ppm, correlating with a de-shielding effect observed for the N-heterocycle of pyridine-like subunit in 4 (Fig. 2B). The same position in 1 shows a similar chemical shift (*δ* = 8.32 ppm, Fig. 2C), consistent with noticeable influence of the imine character of the C–N interaction. The other resonance lines assigned to [4,7]phenanthrolino[5,6-*f*][4,7]phenanthroline or *m*-phenylenes did not show any significant change in chemical shifts that should be correlated with the absence of global diatropic current. Actually, the fully fused derivative 1 accommodates 46 π-electrons, suggesting the possibility of having a fully conjugated hydrocarbon with nitrogen bridges actively participating in electron conjugation and delocalisation within macrocyclic systems,^40,41^ because of its flexibility in the amine–imine couple.

**Fig. 2:**
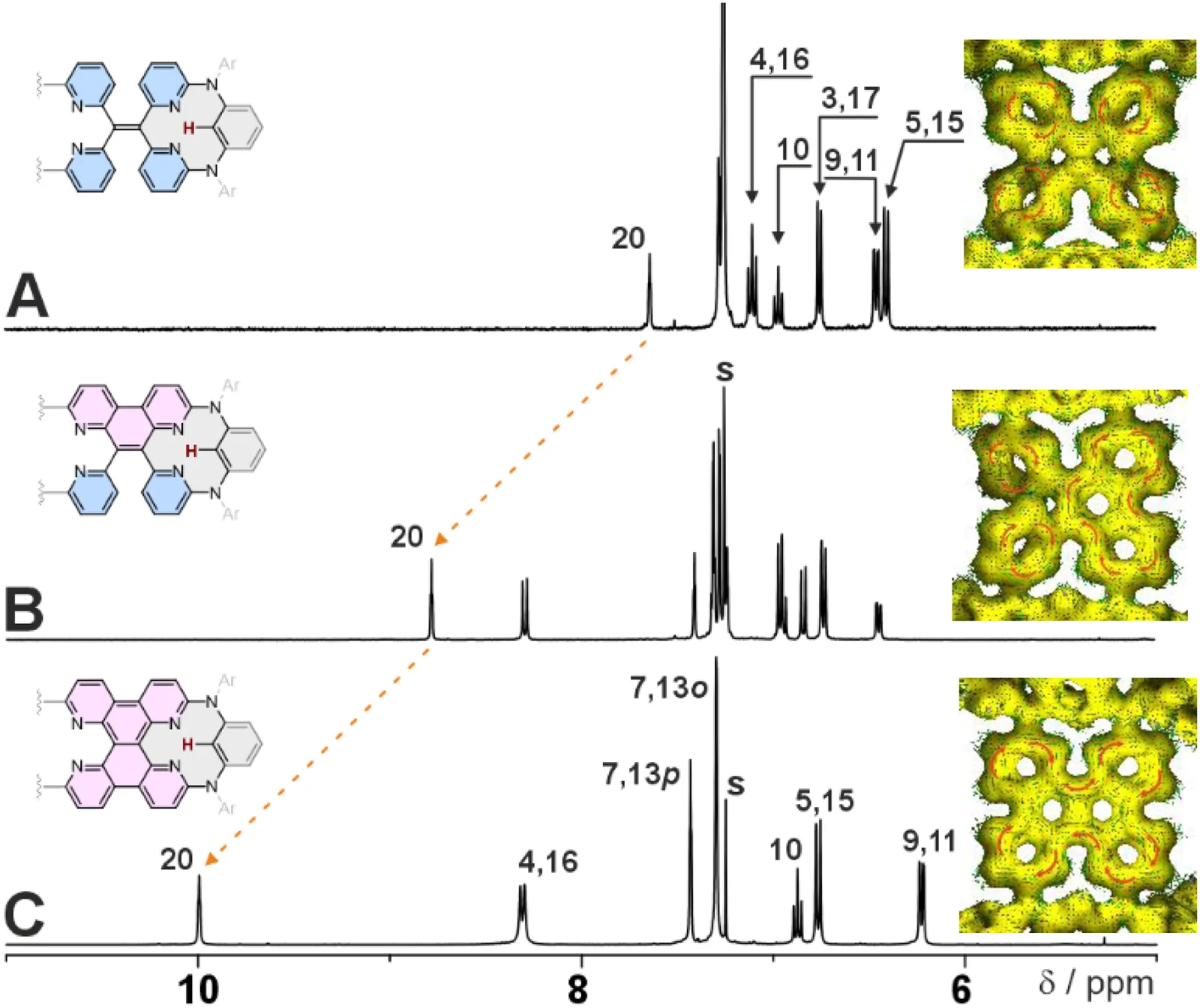
^1^H NMR spectra of 3 (A), 4 (B) and 1 (C). All samples were measured in CDCl_3_ at 298 K. The assignment follows the numbering system presented in Scheme 1. Insets represent the AICD plots showing the change in conjugation in the aza-nanographene unit.

The significantly limiting factor for global conjugation was assigned to the presence of *m*-phenylene that, in contrast to *o*-^42^ or *p*-substitution,^25,43,44^ breaks this possibility. Nevertheless, the formation of 4 and eventually 1 introduces two derivatives into the family of defected nanographene-like systems that incorporate 4,7-phenanthroline and [4,7]phenanthrolino[5,6-*f*][4,7]phenanthroline, respectively, creating a strongly electron-withdrawing core. The planned fusion and forced planarization of the 6.6.6 defect-decorated derivatives extend the conjugation, incorporating phenanthroline-like units that, in the fully fused derivative 1, increase the conjugation path to potential 26 π-electrons entrapped in the central unit of tetraaza-dibenzo[*g*,*p*]chrysene. The possible resonance structures present the anticipated possibility of competition between local and global conjugation operating within the central unit (see the SI, Scheme S2). To unequivocally determine these properties, the theoretical analysis (see the SI) was conducted to get the spectroscopic parameters using the GIAO, NICS^45^ and AICD^46,47^ approaches. The analysis of magnetic properties within all dimeric systems (GIAO and NICS) correlates with the spectroscopically observed behaviour (see the SI, Tables S9 and S10), also reproducing a gradual down-field relocation of H(20) in ^1^H NMR spectra. The AICD analysis for all three dimeric structures showed a picture consistent with gradually increased planarity and sequential extension of the conjugation from solely 6 π-electron conjugation paths characteristic for each carbo-/heterocyclic unit in 3 (Fig. 3A) to an extended conjugation of 26 π-electrons documented for 1 (Fig. 3C). The protonation experiment performed for 1 in the step-by-step approach and monitored by ^1^H NMR spectroscopy showed a gradual accommodation of hydrogen by both defects, eventually leading to 1-2H (Scheme 1 and Fig. S31). Accommodation of hydrogens in 6.6.6 defects changes the ^1^H NMR picture, very subtly shifting H(20) to *δ* = 9.28 ppm, suggesting a weaker C(H)⋯N interaction. This consistently supports the appearance of a new resonance line at *δ* = 19.87 ppm assigned to internal hydrogen(s) of cation(s) (Fig. S31).

**Fig. 3:**
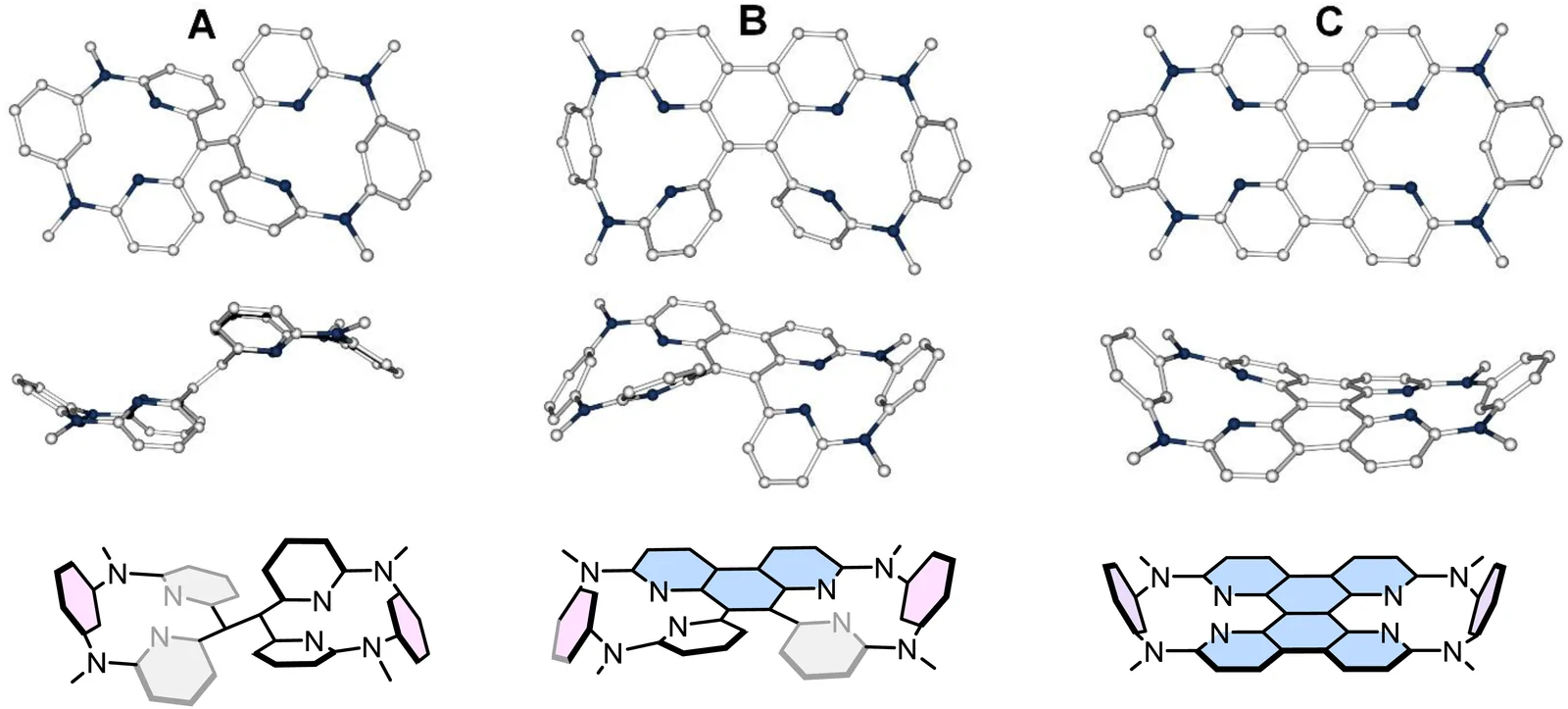
X-ray crystal structures of dimers 3 (A), 4 (B), and 1 (C).^62^ The bottom line presents a graphic representation of observed conformations in each crystal.

A significant de-shielding effect can be correlated with the strong N(H)⋯N interaction observed in the confined space of 6.6.6 defects.^37,38,48^ The protonation process is fully reversible, and the addition of a base (*e.g.*, Et_3_N) recovers unchanged 1.

The sc-X-ray analyses performed for 1, 3 and 4 confirmed a significant influence of gradual fusion on planarization (Fig. 3). The crystal structure recorded for 3 (Fig. 3A) shows a conformation characteristic for sterically repulsive systems with the so-called butterfly effect^37,49–51^ and anti-orientation of pyridines and *m*-phenylene in each 6.6.6 defect that reduces potential steric hindrance. The double bond linking both 6.6.6 defects in 3 was recorded with a typical length of 1.355 Å and a strong deviation from planarity caused by the steric repulsion between pyridine heterocyclic subunits (Fig. 3A). The steric hindrance is partially reduced in the 3 → 4 transition that introduces a 4,7-phenanthroline structural motif but retains the repulsion of two unfused pyridines that are oriented in opposite directions (Fig. 3B). Thus, it sustains the anti-orientation of both *m*-phenylenes observed for 3. The obtained crystal structures for non-fused (3) and semi-fused (4) chromophores show a packing arrangement that isolates each unit, which adequately correlates with the observed non-planarity. The crystal structure of 1 (Fig. 3C) unequivocally confirms the full planarization of the central unit but also shows slight twisting observed in the central unit, with a dihedral angle of ∼16° (Fig. 3C). scXRD analysis for 1-2H showed even more effective planarization (see the SI, Fig. S60).

### Optical and TADF properties

Gradual fusion changes the observed optical properties, gradually shifting the absorption bathochromically for 3, 4 and 1, with the most shifted transitions recorded at *λ*_max_ = 380 nm, *λ*_max_ = 450 nm and *λ*_max_ = 510 nm, respectively. The documented planarization also affects the spectrum shape that for 1 shows a set of well-developed transitions in the green region with increased oscillator strength and narrowed half-width (Fig. 4A1, olive). The solution-based experiments (Fig. 4, trace A2) performed in cyclohexane showed a significant change in the observed emission wavelength that correlates with increasing rigidity in the analysed molecule in the sequence of 3 → 4 → 1, eventually also changing the shape of the observed band. The registered wavelengths of fluorescence (*λ*_em_ = 459 nm (3), *λ*_em_ = 514 nm (4) and *λ*_em_ = 518 nm (1)) and the resulting substantially different Stokes shift (∼89 nm (3), ∼65 nm (4) and ∼8 nm (1)) along with the shape of observed transitions correlate with dissimilar overall rigidity and similarity of the excited and ground state^52^ present in 1. It finds its reflection in prompt fluorescence quantum yields (*Φ*_PF_) and decays (S_1_ lifetime, *t*_1_) eventually registered as *Φ*_PF_ = 0.08 (*t*_1_ = 4.7 ns (3)), *Φ*_PF_ = 0.16 (*t*_1_ = 5.4 ns (4)) and *Φ*_PF_ = 0.43 (*τ*_PF_ = 8.8 ns (1)). These parameters end up in significant difference in the contribution of radiative (*k*_r_ = 1.7 × 10^7^ s^−1^, 2.9 × 10^7^ s^−1^, and 4.8 × 10^7^ s^−1^) and non-radiative (*k*_nr_ = 1.9 × 10^8^ s^−1^, 1.6 × 10^8^ s^−1^, and 6.6 × 10^7^ s^−1^) constants. The registered fluorescence bands of all three dimeric motifs show a different shape that sharpens in the row 3 → 4 → 1, eventually showing a FWHM of 20 nm recorded for 1 (cyclohexane solution, *c* = 5 × 10^−5^ M) with respect to 70 nm (3) and 74 nm (4). The half-width increases to 51 nm while switching solvent to Me-THF (Fig. 4B3, green) but the wavelength remains similar (*λ*_em_ = 532 nm). Beside fluorescence, 1 also exhibits phosphorescence recorded at 77 K and manifested as the radiative transition recorded at *λ*_phos_ = 564 nm (*τ* = 710 ms). Based on *λ*_max_ for both radiative processes, the Δ*E*_ST_ value of 0.132 eV was calculated, fitting the expected range for observation of TADF.^6,7^ The compounds 3 and 4 also showed phosphorescence observed at *λ*_phos_ = 577 nm (*τ* = 96 ms) and *λ*_phos_ = 571 nm (*τ* = 223 ms). The room-temperature experiments for 1 (solution) showed a change in the emission wavelength, suggesting re-population of the singlet state (S_1_) and the presence of reverse intersystem crossing (rISC) responsible for thermally activated delayed fluorescence (TADF). The detailed analysis performed for cyclohexane solution showed an identical shape and position of the registered emission spectrum (Fig. 4C1 and C2) in the regime of ns (9.5 ns, olive) and µs (0.36 µs, grey), consistent with the presence of the rISC process and eventually thermally activated delayed fluorescence. The solution experiments performed for 3 and 4 (see the SI file) under similar conditions did not show any signs of TADF. The TADF properties were further explored in thin-film experiments performed in two matrices with significantly different polarity – non-polar ZNX™ (ZEONEX™) and polar PVP (polyvinylpyrrolidone). Under all studied conditions, fluorophore 1 exhibits not only prompt fluorescence (PF) but also a significant portion of delayed emission, appearing to be delayed fluorescence (DF), as confirmed by its spectral identity to PF (Fig. 4C3, olive). The analysis of thin-film behaviour started with the experiments performed for 0.1% (w/w) concentration of 1 in ZNX to understand the dynamic of the directly excited S_1_ state. The recorded green emission was registered at *λ*_max_ = 522 nm and the prompt fluorescence lifetime of *τ*_PF_ = 9.6 ns (Table 1). The estimated radiative rate constant (*k*_r_) reaches 3.5 × 10^7^ s^−1^, which is considerably higher than that of typical strongly twisted D–A TADF emitters and approaches the range commonly observed for more planar SR-CT/MR-type emitters. In ZNX, the non-radiative rate constant is much lower (*k*_nr_ < 0.2 × 10^7^ s^−1^), while intersystem crossing dominates the S_1_-state depopulation: *k*_ISC_ = 6.5 × 10^7^ s^−1^. Such efficient population of the excited triplet state results in the delayed emissions. Upon cooling, the native triplet state emission appears as a long-lived phosphorescence component, which starts to dominate below 110 K. The singlet–triplet energy gap, Δ*E*_ST_, was estimated at 10 K from the onsets of the prompt fluorescence and phosphorescence spectra, giving a value of 0.16 eV (see the SI, Fig. S45). Owing to such a small energy gap, the delayed fluorescence dominates completely at room temperature. Under ambient conditions, the DF lifetime (*τ*_DF_) is quite short, 14 µs, and the total PLQY is ∼40%, which most probably is caused by triplet quenching by oxygen. In a vacuum, *τ*_DF_ reaches 190 µs, indicating much larger DF contribution, which is accompanied by an increase in the total PLQY to an almost quantitative value. Such strong oxygen sensitivity further supports the involvement of the T_1_ state in the delayed emission process (see the SI, Fig. S44 and S46). To further verify the TADF character of 1, more detailed temperature-dependent time-resolved emission measurements were performed (see the SI, Fig. S47 and Tables S4, S5), unequivocally confirming the presence of delayed fluorescence. Upon cooling, the DF intensity and *k*_rISC_ decrease, while the DF lifetime increases, reaching the level of 0.056 × 10^3^ s^−1^ and 2.7 × 10^4^ µs, respectively. Analysis of the temperature dependence of the rISC rate using an Arrhenius plot (Fig. 4D1, see the SI, Table S8) gave an activation energy of *ca.* 130 meV, in good agreement with the spectroscopically determined Δ*E*_ST_ value (Table 1).

**Fig. 4:**
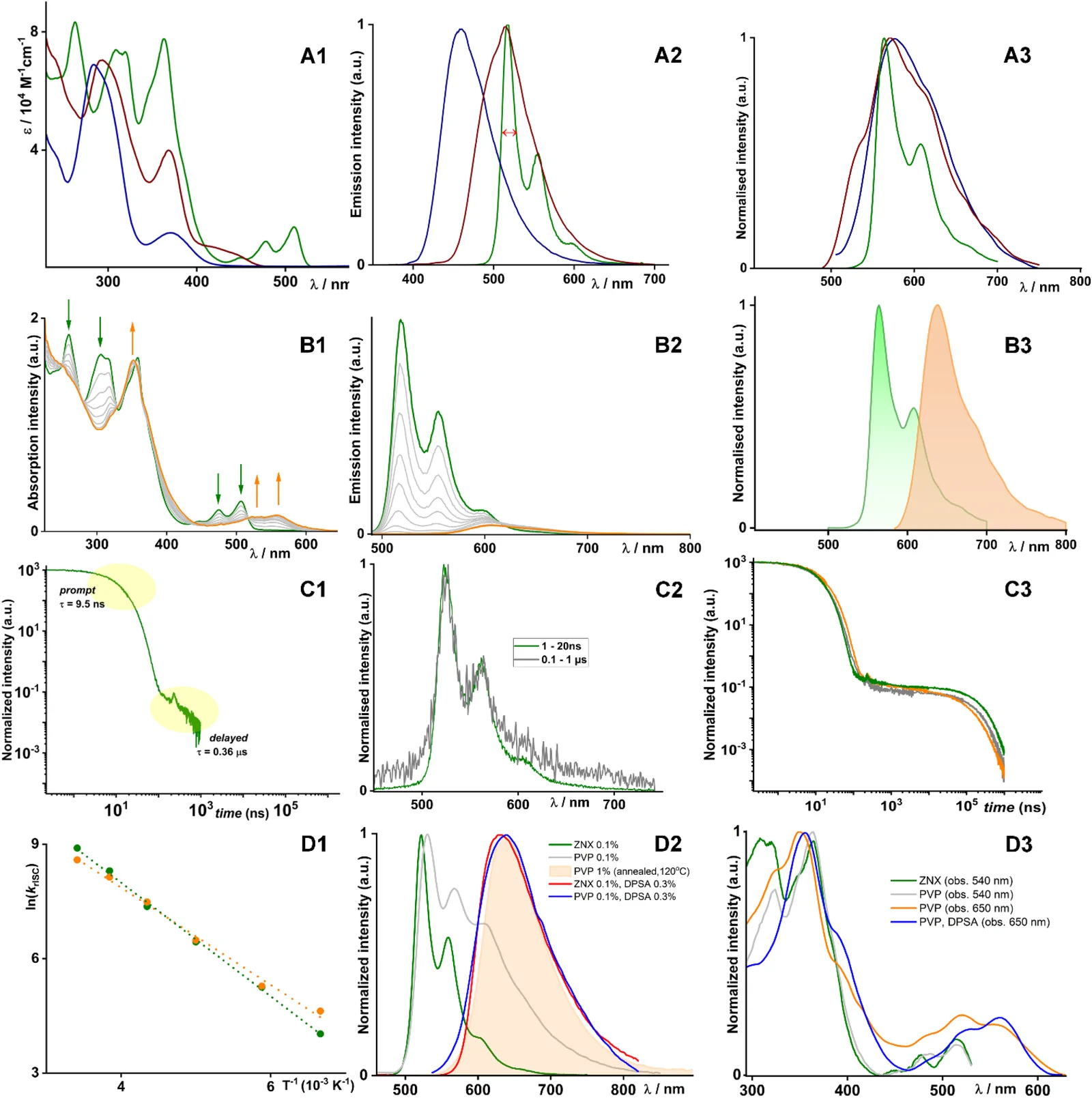
UV-vis absorption and emission experiments: trace (A1) absorption spectra of 1 (olive), 3 (navy), and 4 (wine) in cyclohexane, 298 K; (A2) fluorescence spectra of 1 (olive), 3 (navy), and 4 (wine) in cyclohexane, 298 K; (A3) phosphorescence spectra of 1 (olive), 3 (navy), and 4 (wine) in MeTHF, 77 K; (B1) absorption monitored TFAH titration of 1 (dichloromethane, 298 K) with formation of 1-2H; (B2) fluorescence monitored TFAH titration of 1 with formation of 1-2H (dichloromethane, 298 K); (B3) phosphorescence of 1 (olive, 298 K) and 1-2H (orange, 77 K) (MeTHF); (C1) delayed fluorescence profile for Ar-saturated solution of 1 (cyclohexane, RT); (C2) prompt (olive, 9.5 ns) and delayed fluorescence (grey, 0.36 µs) tested for the fluorescence decay from trace (C1) at the yellow highlight; (C3) delayed fluorescence profiles for films measured in a vacuum of 1 in ZNX (0.1%, olive, green band), PVP (0.1%, grey, green band), and PVP (1%, orange); Arrhenius plot (D1) showing the temperature dependence of the rISC rate constant of 1 (olive) and 1-2H (orange) measured in a ZNX film (for input data, see Tables S5 and S7; for Arrhenius fitting parameters, see Table S8); emission spectra (D2) and excitation spectra (D3) of 1 and 1-2H in polymer films (see the legend in each trace for a detailed description).

**Table 1:** TADF data obtained for 1/1-2H in thin film experiments (vacuum, 293 K)

Sample	*λ*_PF_ [nm]	*λ*_phos_ [nm]	Δ*E*_ST_ [eV]	*E*_a_ a	PLQY^b^	*τ*_PF_ [ns]	*τ*_DF_ [µs]	*k*_r_ [×10^7^ s^−1^]	*k*_ISC_ [×10^7^ s^−1^]	*k*_nr_ [×10^7^ s^−1^]	*k*_rISC_ [×10^4^ s^−1^]
1/ZNX	522	577	0.16	0.128 ± 0.003	93	9.6	190	3.5	6.5	0.2	1.4
1-2H/ZNX^d^	630	645	0.10	0.110 ± 0.004	29	6.8	195	4.1	0.7	9.0	0.54
1/PVP	533	—c	—	—	84	11.3	157	3.9	4.2	0.7	1.2
1-2H/PVP^e^	635	—c	—	—	50	11.5	114	2.6	3.5	2.6	1.5
1-2H/PVP^d^	635	—c	—	—	47	12.4	96	2.5	3.0	2.8	1.6

a Activation energy obtained from Arrhenius plots (Fig. 4D1 and Table S8).

b Values reconstructed for vacuum [%].

c Phosphorescence was not determined for the PVP films due to limited film stability and the difficulty of collecting a reliable signal.

d Obtained with additions of DPSA (0.3%).

e Annealed at 393 K for 5 min.

These results confirm that DF of 1 originates from thermally activated repopulation of the S_1_ state from T_1_. Both defects present in 1 create a basic environment ready to entrap a proton, eventually modulating the optical outcome. The ^1^H NMR monitored protonation (Fig. S31) showed a step-by-step process for a sequence of 1 → (1-H) → 1-2H interconversions. The UV-vis monitored trifluoroacetic acid (TFAH) titration of 1 (Fig. 4B1) showed an isosbestic transformation, with bathochromically shifted transitions eventually recorded at 568 nm. The presence of internal hydrogen in 6.6.6 or 7.6.6 defects^4^ has been reported as a factor that fully quenches photoluminescence or significantly reduces the efficiency of quantum yield. 1-2H belongs to the second group that sustains the fluorescence recorded at *λ*_max_ = ∼620 nm, but with a drastically reduced *Φ*_PF_ of 4%. In solutions, similarly to 1, the protonated form shows efficient phosphorescence at 77 K, recorded at *λ*_phos_ = 638 nm (*τ* = 42 ms, Me-THF). The extracted Δ*E*_ST_ gave a value of 0.046 eV (Me-THF), also consistent with potential TADF behaviour. The low-temperature experiments (liquid nitrogen) performed for the 1-2H sample (Me-THF) showed a change in the emission wavelength, suggesting re-population of the singlet state (S_1_) and the presence of reverse intersystem crossing (rISC) responsible for thermally activated delayed fluorescence (TADF).

Next, we investigated the effect of protonation on the excited-state dynamics and TADF properties in films. Upon doping the ZNX film with dodecyl sulfonic acid (DPSA), the protonated form 1-2H is formed, as evidenced by the appearance of a broad, structureless emission band red-shifted to 630 nm (Fig. 4D2). Under these conditions, the PLQY of 29% is quite high for the protonated TADF emitter, though it is still lower than that of neutral 1. This properly correlates with the postulated mechanism of increased contribution of the non-radiative depopulation channel, correlating with the dynamics of hydrogen-bond-like interactions and their possible tautomerism.^23^ This is mainly due to a strong increase in the non-radiative deactivation rate from the S_1_ state, which becomes the dominant deactivation pathway (Table 1). The photophysical parameters remain relatively comparable to those of the neutral species, but protonation suppresses ISC (from 6.5 × 10^7^ to 0.7 × 10^7^ s^−1^) and noticeably reduces the rISC rate (from 1.4 × 10^4^ to 0.54 × 10^4^ s^−1^). The protonated form 1-2H also clearly shows temperature-dependent delayed fluorescence. As in the case of neutral 1, cooling the 1-2H sample leads to a decrease in delayed fluorescence intensity and an increase in its lifetime, confirming the thermally activated character of the delayed emission (Fig. S47 and Tables S6, S7). In addition, an almost twofold increase in the prompt fluorescence lifetime is observed at lower temperature, indicating that the non-radiative deactivation of 1-2H is also thermally assisted. The Δ*E*_ST_ value estimated from the onsets of prompt fluorescence and low-temperature phosphorescence is 0.10 eV (Table 1). Arrhenius analysis of the rISC rate (Fig. 4D1, see the SI, Table S8) gives an activation energy of *ca.* 110 meV, which agrees well with the spectroscopically determined Δ*E*_ST_. Notably, the preexponential factor, which carries the information on S_1_–T_1_ SOC, is reduced for 1-2H,^54^ indicating weaker coupling between the lowest excited singlet and triplet states compared to 1 (see the SI, Table S8). Thus, protonation documented in films does not suppress TADF but generates a distinct red-emitting TADF-active form with a smaller singlet–triplet gap and *E*_a_.

We also investigated the behavior of 1 in the more polar and hydrophilic PVP polymer matrix. In this medium, the emission band corresponding to neutral 1 is slightly red-shifted to 533 nm (Fig. 4D2). When monitored in this spectral region, the excitation spectrum (Fig. 4D3) shows no significant changes compared with that of neutral 1 in ZNX, confirming that the same emissive species is observed. In PVP, this band shows only a slight decrease in PLQY to 84%, while *τ*_PF_ is elongated to 11.3 ns, mainly due to a decrease in the ISC rate. Consequently, owing to the reduced ISC quantum yield (*F*_ISC_ = 48%), *t*_DF_ decreased to ∼160 µs, while the rISC rate remains within the same range (Fig. 4C3, grey). Interestingly, even freshly prepared acid-free PVP films containing a low concentration of 1 (0.1% w/w) show spectral broadening in the red region, indicating the presence of an additional emission band. In contrast, when monitored at 650 nm, the excitation spectrum (Fig. 4D3) appears as a superposition of the neutral form and an additional band (Fig. 4D2), indicating that these species are already present in the ground state. To understand the origin of the latter, we examined the fluorescence of 1-2H in PVP films doped with 0.3% DPSA (Fig. 4D2). In this case, a single broad emission band appears at around 635 nm, while the excitation spectrum (Fig. 4D3), with a long-wavelength maximum at approximately 560 nm, matches the additional bands observed in acid-free PVP films. This confirms that a significant fraction of 1 is already protonated in the acid-free PVP matrix. Moreover, annealing the neutral PVP film at 120 °C changes the emission from a combination of green and red bands to a spectrum completely dominated by the red band. Both this transformation and the dual-band emission observed in neutral PVP films can be attributed to protonation of 1 by trace amounts of water retained in the polymer matrix or diffusing from air; this process is facilitated by heating and the resulting increase in diffusion rate. Compared with the DPSA-doped ZNX film, the protonated form 1-2H shows improved photophysical parameters in PVP: its PLQY reaches 50%, mainly due to a reduced *k*_nr_, which becomes comparable to *k*_r_. In addition, *k*_rISC_ is increased, resulting in a shortened delayed fluorescence lifetime of 110 µs. These results indicate that the PVP matrix is more favorable for the protonated form than ZNX, enabling more efficient triplet harvesting and reducing energy losses. As a result, the protonated TADF form becomes photophysically comparable to the neutral emitter. Overall, the relatively high *k*_rISC_ values, reaching up to 1.5 × 10^4^ s^−1^ for both 1 and 1-2H, place them in a range comparable to typical MR-type TADF systems.^55^

### Theoretical analysis

A deeper understanding of observed behaviour requested a broad theoretical analysis of ground and excited states for available variants of 1 and 1-2H defected nanographene motifs. As originally postulated at the level of the structural design, the incorporation of nitrogen introduces a subtle balance between the donating and accepting character depending on the structural scheme.^2,3,36,37^ The original construction with the novel idea of N-doped nanographene with a balance between electron-donating (amine) and electron-accepting (imine) groups imprinted in 1 shows an organization where the donor groups (diarylamines) effectively surround the acceptor unit of tetraaza-dibenzo[*g*,*p*]chrysene, eventually forming a symmetrical chromophore/fluorophore of quadrupolar construction. These constructions do not correlate either with LR-CT nor canonical characteristics for MR-TADF even if donor/acceptor parts can be easily distinguished. Nevertheless, the recent studies have demonstrated that CT-ES can be stabilized within rigid, planar π-conjugated frameworks, as exemplified by the planar ICT-TADF systems reported by Kulia, Monkman and co-workers,^56^ and the SR-CT chromophores described by Zou *et al.*^57^

The optimization of structures for 1 and 1-2H with B3LYP/cc-pVDZ level^58^ theory started from a GaussView created model that eventually showed geometries reproducing the one observed in the crystal and proving the correctness of theoretical approach's choice. Both geometries showed significant planarity that deepens with proton binding in 1-2H (Fig. 5, trace A and B). The LUMOs in both derivatives are located solely on the central unit of tetraaza-dibenzo[*g*,*p*]chrysene as the most electron deficient subunit (Fig. 5, traces A and B), while the HOMOs in both 6.6.6 defected systems, *i.e.*1 and 1-2H, correlate with substantial contribution of the electron-donating character of amine substituents (Fig. 5). It adequately correlates with the postulated influence of two types of nitrogen present in the nanographene unit, *i.e.* amine donor and imine acceptor. The distribution of both orbitals shows a substantial mutual overlap (Fig. 5A and B) that differs from MR-TADF but keeps a possibility of SR-CT important for rISC. The anticipated energies show substantial reduction of the HOMO–LUMO gap from 2.88 eV for 1 to 2.36 eV for 1-2H, correlating with the observed bathochromic shift upon protonation. The observed energies show a drastic stabilisation of both the HOMO and LUMO while passing from 1 to 1-2H by 5.33 eV and 4.89 eV, respectively. The adiabatically optimized S_1_ and T_1_ states are predicted at energy levels of 2.25 and 1.97 eV for 1 and 1.63 and 1.43 eV for 1-2H, leading to Δ*E*_ST_ of 0.277 and 0.200 eV, respectively, for B3LYP/cc-pVDZ.^58,59^ The size and structural complexity of 1/1-2H precludes the use of more accurate functionals (*e.g.* double hybrid B_2_PLYP) reported as more accurate, and the application of other functionals, but as noticed, the theoretically predicted Δ*E*_ST_ for both analysed systems shows a good correlation with experimental values (0.16 and 0.10 eV, respectively), and we can infer that these values will be fully reproduced with the application of a higher level of theory.

**Fig. 5:**
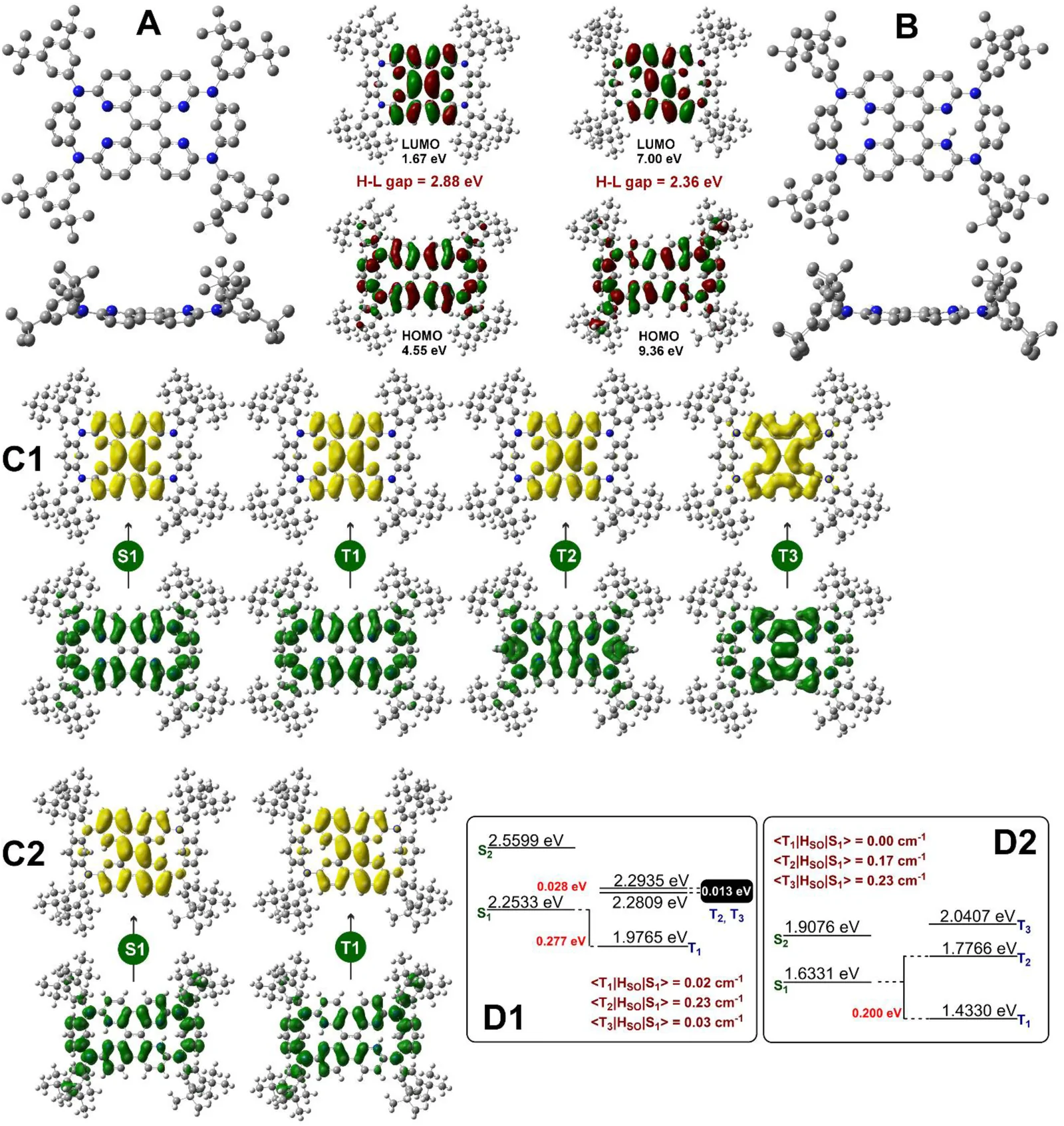
Theoretical results for 1 and 1-2H. DFT-optimized geometries of ground states (B3LYP/cc-pVDZ) and HOMOs/LUMOs (iso-value: 0.02, wine – positive, olive – negative) for 1 (trace (A)) and 1-2H (trace (B)) obtained for the gas phase. Trace (C and D) show excitation transitions (iso-value: 0.02, yellow – electron, green – hole) and the energy levels, along with spin–orbit coupling values, calculated for optimized geometries of the T_1_ state for 1 (S_1_ and T_1_, T_2_, T_3_) and 1-2H (S_1_ and T_1_), respectively.

The TD-DFT (TDA-DFT) analysis at the same level of theory showed a set of transitions assigned either to singlet or triplet states. The most bathochromically shifted transition, assigned to S_0_ → S_1_, corresponds to electron transfer between HOMOs and LUMOs for neutral 1 (97.43%) and protonated 1-2H (98.6%), as documented by the obtained NTOs (Fig. 5, trace C). Similarly to S_1_, the T_1_ NTOs obtained for the optimized geometries are also connected with HOMOs and LUMOs (Fig. 5, trace C), defining the hole and electron, respectively, for 1 and 1-2H (Fig. 5, trace C). The distribution of state energies for 1 shows the S_1_–T_1_ adequately corresponding with experimental data (Fig. 5, trace D). The T_2_ and T_3_ states obtained for 1 lie slightly higher (+0.028 eV (T_3_) and +0.040 eV (T_2_)) than S_1_ and show almost similar energies differed by 0.012 eV, favouring T_3_. Doubly protonated form 1-2H reorganizes the energy levels, eventually substantially stabilizing T_2_ with respect to T_3_ and increasing the energy of the latter by ∼0.3 eV with respect to T_2_. Both triplet states (T_2_ and T_3_) remain at higher energies than S_1_ (Fig. 5, traces D1 and D2), also increasing the S_1_–T_2_/T_3_ energy gaps compared with those of 1. This suggests that S_1_ and T_1_ states have a partial CT nature, with a significant change in the transition dipole moment, and are stabilized by protonation of the acceptor. In contrast, T_2_ has a pure locally excited (^3^LE) character that is less affected by protonation; thus, its energy does not change substantially. The stability of the T_2_ state in 1 plays an important role while looking at SOC matrices (calculated for pre-optimized T_1_ geometries of 1 and 1-2H (Fig. 5C and D)).^53–59^ For the neutral form, a small energy difference between S_1_ and almost degenerate T_2_/T_3_ (*i.e.* 0.028 eV (T_3_) and 0.040 eV (T_2_)) suggests their involvement in the rISC process. The T_1_–S_1_ spin–orbit coupling obtained for the pre-optimized T_1_ geometry shows values of 0.02 cm^−1^ (1) and 0.00 cm^−1^ (1-2H), which is consistent with SR-CT behaviour, as strong T_1_–S_1_ coupling characteristic for LR-CT fluorophores usually does not occur in SR-CT systems. The SOC values documented for higher triplet states of 1 show a high value for T_2_ (0.23 cm^−1^) and negligible for T_3_ (0.03 cm^−1^). It additionally suggests a significant involvement of T_2_ in ISC/rISC processes, potentially *via* spin–vibronic coupling.^35^ In contrast to that, the protonated 1-2H shows a SOC value comparable to 1 of 0.17 cm^−1^ for T_2_–S_1_ and a noticeably higher one for T_3_–S_1_ (0.23 cm^−1^). The geometries obtained for T_3_ states of 1 and 1-2H show significant differences that correlate with changes of parameters. The presence of internal hydrogens in 1-2H prevents the skeleton from twisting documented for the T_3_ state in 1 and leads to a more planar geometry forced by protonation of both 6.6.6 defects, causing strong H-bonding with both imine nitrogens. Following these observations, it can be concluded that in both variants of 1, either neutral or dicationic, the efficient TADF follows the SR-CT that intensifies with a significant rigidity and planarity of the fluorophore that stabilizes the S_1_(T_1_) states, promoting both ISC/rISC non-radiative processes and lowered exchange energy and reduced Δ*E*_ST_.^60^

## Conclusions

The modulation of emission properties supported by the possibility of observing the change of the emission wavelength and moving toward the red region constantly remains an important aspect of contemporary research because of several practical aspects.^61^ Significant attention is given to the behaviour that can be revealed under specific conditions in response to external factors.^26,27^ This contribution brings an insight into the construction of nanographene-like derivatives additionally decorated with 6.6.6 defect(s). The newly designed chromophore/fluorophore shows green fluorescence that can be modulated with a fundamental transformation, *i.e.* protonation eventually leading to red emission. As documented with the support of experimental and theoretical analyses, the new structural design has an efficient and narrow (FWHM = 20 nm) fluorescence observed in solution but also shows efficient *k*_ISC_, populating the T_1_ state documented with phosphorescence. The effective rISC re-populates the S_1_ state that leads to the TADF behaviour following the SR-CT and increased PLQY to 93% recorded for thin-films in the green region (*λ* ∼ 520 nm). The reversible protonation shifts the emission to the red region (*λ* ∼ 620 nm) with decreased *Φ*, but efficient rISC is observed in polymer films, boosting the PLQY to 50% in the red region. Thus, the obtained defected nanographene introduces a new molecular design of a highly symmetric fluorophore showing TADF behaviour, with charge redistribution occurring within a rigid, symmetric molecular framework with a strong overlap of frontier orbitals following the SR-CT character of the excited state. The final molecule creates a unique system showing efficient thermally activated delayed fluorescence in two chemically different stages of the same derivative, as documented for the first time in neutral and cationic forms. Thus, the subtle balance between the two stages that efficiently emit in two optical windows, switching to the very desired red region,^62,63^ creates an efficient TADF system that occupies an intermediate position between conventional CT-TADF and MR-TADF families, eventually opening further possibilities for designing and obtaining highly symmetrical structures with modulated and efficient thermally activated delayed fluorescence that are currently underway in our laboratory.

## Author contributions

Conceptualization: MP; methodology: RVS, IES, KD, ŁO, MK, MP. Investigation: RVS, IES, KD, ŁO, TMM, MK, MS. Visualization: MP, RVS. Supervision: MP. Writing – original draft: MP. Writing – review & editing: RVS, IES, KD, ŁO, MK, TMM, MS, MP.

## Conflicts of interest

There are no conflicts to declare.

## Supplementary Material

SC-OLF-D6SC06038B-s001

SC-OLF-D6SC06038B-s002

SC-OLF-D6SC06038B-s003

SC-OLF-D6SC06038B-s004

SC-OLF-D6SC06038B-s005

SC-OLF-D6SC06038B-s006

SC-OLF-D6SC06038B-s007

SC-OLF-D6SC06038B-s008

SC-OLF-D6SC06038B-s009

SC-OLF-D6SC06038B-s010

SC-OLF-D6SC06038B-s011

SC-OLF-D6SC06038B-s012

SC-OLF-D6SC06038B-s013

## Data Availability

CCDC 2533581 (4) and 2540680–2540682 (2, 3 and 1) contain the supplementary crystallographic data for this paper.^64*a*–*d*^ Supplementary information (SI): experimental procedures, NMR spectra, HRMS data, photophysical measurements, computational details, optimized geometries and TD-DFT calculations. See DOI: https://doi.org/10.1039/d6sc06038b.
