# Supplementary material for: Thermally activated delayed fluorescence in neutral and cationic stages of aza-nanographene decorated with 6.6.6 defects
Source: Chem Sci. 2026 Oct 9. Online ahead of print. doi: 10.1039/d6sc06038b (PMC13678670; doi:10.1039/d6sc06038b)
Supplement: Supplementary file 1 [file SC-OLF-D6SC06038B-s001.pdf]

Supplementary Information (SI) for Chemical Science.
This journal is © The Royal Society of Chemistry

Contents

1. General information ... 2
2. Experimental section ... 4
  2.1 Experimental procedures ... 4
3. NMR spectra ... 6
4. UV-VIS spectra ... 22
5. Emission spectra ... 23
6. Lifetime Measurement ... 26
7. TADF experiments ... 29
8. Cyclic voltammograms ... 37
9. ESI-MS spectra ... 39
10. Theoretical calculations ... 42
  10.1 NICS calculations ... 42
  10.2 GIAO calculations ... 42
  10.3 RMSD ... 43
  10.4 Reorganizational Energy ... 43
11. Cartesian Coordinates. ... 44
12. X-Ray Analysis ... 45

1. General information

NMR Spectroscopy. ^{1}H NMR spectra were recorded on a high-field spectrometer (^{1}H 600.15 MHz, 400 MHz and 300 MHz, ^{13}C 151 MHz and 100 MHz), equipped with a broadband inverse gradient probe head. Spectra were referenced to the residual solvent signal (chloroform-d, 7.26 ppm and dichloromethane-d_2 5.32 ppm in ^{1}H spectra and chloroform-*d*, 76.16 ppm and dichloromethane-d_2, 53.00 ppm in ^{13}C spectra). Two-dimensional NMR spectra were recorded with 2048 data points in the t_2 domain and up to 1024 points in the t_1 domain, with a 1s recovery delay.

Mass Spectrometry. The ESI-MS analysis was performed on Bruker qTOF (Bremen, Germany) equipped with a standard ESI source. Spectra were recorded using DCM or MeOH. The instruments parameters were as follows: positive-ion mode, calibration with the Tunemix™ mixture (Agilent Technologies, Palo Alto, CA, USA), mass accuracy was better than 5 ppm, scan range: 50 - 1600 m/z; drying gas: nitrogen; flow rate: 3.0 L/min, temperature: 200 ^{o}C; potential between the spray needle and the orifice: 4.5 kV, analyte was introduced to the ESI source by pump (New Era Pump Systems, Inc., USA) with a flow rate: 3 µL/min.

UV-Vis Spectroscopy. Electronic spectra were recorded on a Varian Carry-50 Bio spectrophotometer with a 1 cm optical path in solutions in HPLC grade CH_2Cl_2 in 298 K using standard quartz cuvettes.

Fluorescence. Steady state fluorescence spectra were recorded with a Hitachi F-7000 Spectro-fluorometer apparatus. Time-dependent experiments (life-time measurements) was conducted with a picosecond Horiba Jobin Yvon spectrofluorometer (UV-Vis-NIR). The fluorescence quantum yield was established using a 9, 10 diphenyl anthracene as a reference (QY = 0.96 in cyclohexane) by a relative approach.

TADF Experiments.

Materials. Zeonex® (ZNX, Zeonex480R, density = 1.01 g/cm^3) or polyvinylpyrrolidone (PVP, 40 000 Mw) were purchased from Zeon Europe GmbH and Sigma-Aldrich, respectively.

Sample Preparation. The typical concentration of dye in the measured solutions was 0.01 mg/mL. Photoluminescence (PL) experiments were conducted in 1x1 cm quartz cuvettes under both air and argon atmospheres. Studies on the conversion of the green to red emission band involved irradiation with laser light at selected wavelengths (beam diameter of 3 mm), with an output power of 50 µW at the sample surface. Polymer films doped with the dye were prepared using drop-casting or spin-coating methods under ambient air conditions, from solutions containing mixtures of the polymer and dye in appropriate ratios. ZNX was dissolved in toluene or chlorobenzene, PVP was dissolved n chloroform. For drop-cast films, the samples were annealed on a hot plate at 80-120 °C to remove residual solvent. PL experiments on the films were performed under both air and vacuum conditions.

Apparatus. UV-Vis absorption spectra were recorded using a Shimadzu UV-1900 spectrophotometer. Steady-state PL spectra were recorded on the FS5 spectrofluorometer (Edinburgh Instruments, UK) using L-configuration (solutions) or front-face (films) excitation geometry with a 1 nm spectral resolution. Absolute PL quantum yields (PLQYs) were measured using an integrating sphere. Time-resolved measurements were performed using a customised system consisting of a pulsed YAG:Nd laser (PL2251A, EKSPLA) coupled with an optical parametric generator (PG 401/SH) as the excitation light source and 2501S grating spectrometer (Bruker Optics) combined with a streak camera system (C4334-01 Hamamatsu) as the detection unit).[1] The system was equipped with a double-stage high vacuum pump (T-Station 85 Edwards) for measurements under vacuum conditions. Streak camera images were integrated over a constant specified wavelength interval to build PL intensity decay profiles. Phosphorescence measurements in films were recorded using a closed-cycle helium cryostat (APD DE-202) and a temperature controller (LakeShore 336). Origin[2] was used for multiexponential fitting and plotting of emission decay curves.

X-Ray Analysis. X-Ray quality crystals were prepared by diffusion method from HPLC grade DCM / methanol solvent combination (**3**, **4**) and slow evaporation method with DCM for (**2**) and ethyl acetate for (**1**). The measurements for **2** and **3** were performed using Bruker D8 Quest Eco diffractometer at 100K equipped with

[1] A. A. Kubicki, P. Bojarski, M. Grinberg, M. Sadownik and B. Kukliński, *Opt. Commun*. **2006**, *269*, 275−280.
[2] Origin. 2003. Origin 7.5. OriginLab Corp., Northampton, MA.

Photon II CPAD detector, MoKα (λ = 0.71073 Å) sealed tube radiation source and Triumph® optics. The measurements for **1** was performed using a Rigaku Oxford Diffraction XtaLAB Synergy-R DW diffractometer equipped with a HyPix ARC 150° Hybrid Photon Counting (HPC) detector using CuKα (λ = 1.54184 Å) at 100 K. Data was processed using the CrystAlisPro software. The structures were solved by intrinsic phasing with SHELXT (2015 release) and refined by full-matrix least-squares methods based F2 using SHELXL[3] with support of the Olex interface.[4]For all structures, H atoms bound to C atoms were placed in the geometrically idealized positions and treated in riding mode, with C-H = 0.95Å and U_{iso}(H) = 1.2Ueq(C) for C-H groups, and C-H = 0.98Å and U_{iso}(H) = 1.5Ueq(C) for CH_3 groups. The measurement for **4** was performed at 100 K using Rigaku XtaLAB Synergy (Dualflex) diffractometer with HyPix detector and monochromated $^{Cu}K_{\alpha}$ X-ray source (λ = 1.54184 Å). Graphics were prepared with

Theoretical calculations. Geometry optimization for all analysed structures was carried out with the Gaussian 09[5] and Gaussian 16 software package within unconstrained C1 symmetry, with starting coordinates derived from semi-empirical calculations. Becke's three-parameter exchange functional with the gradient-corrected correlation formula of Lee, Yang and Parr (DFT-B3LYP)[6] and M06-2x[7] were used with the 6-31G(d,p)[8] and cc-pVDZ[9] basis set. The polarizable continuum model of solvation was used (PCM, standard chloroform and dichlormethane for parametrization) for prediction of spectroscopic properties.. Harmonic vibrational frequencies were calculated using analytical second derivatives as a verification of local minimum achievement with no negative frequencies observed. The excited states geometries (S1,S2, T1, T2, T3) for **1** and **1**_2H were optimized for adiabatic analysis of energy level by applying the TD-DFT calculation with 'opt' option and by changing the 'root' command accordingly. The electronic spectra were simulated by means of time-dependent density functional theory (TD-DFT) using the Tamm-Dancoff approximation and TDA-DFT for 20 states and 6-31G(d,p) basis set for all the derivatives. The electronic transitions and UV/Vis were analyzed by means of the GaussSum program. The transitions were convoluted by Gaussian curves with 2000 cm^{-1} half-line width for all the derivatives. GIAO predicted chemical shifts for all structures were calculated for fully optimized geometries. The SCF GIAO NMR predictions were determined starting from the optimized geometries and using a shielding value of 31.75 ppm when calculated in $CDCl_3$ and CD_2Cl_2. Shielding values were calculated on the TMS (tetramethyl silane) optimized structure. The analysis of diatropic currents present in analysed systems were performed via calculation of NICS value and AICD currents calculated with Gaussian 09 software and visualized with the software provided by the Authors.[10] The charge distribution has been performed with NBO calculations and visualized with Gauss View 5.0 package. Spin-orbit couplings were calculated with ORCA 6.0 software package for optimised T_1 state.[11]

---

[3] G. M. Sheldrick, Acta Crystallogr. 2015, A71, 3–8; G. M. Sheldrick, Acta Crystallogr. 2015, C71, 3–8.

[4] O. V. Dolomonov, L. J. Boutjis, R. J. Gildea, J. A. K. Howard, H. Puschmann, J. Appl. Crystallogr. 2009, 42, 339–341

[5] Gaussian 16, Revision C.01, M. J. Frisch, G. W. Trucks, H. B. Schlegel, G. E. Scuseria, M. A. Robb, J. R. Cheeseman, G. Scalmani, V. Barone, G. A. Petersson, H. Nakatsuji, X. Li, M. Caricato, A. V. Marenich, J. Bloino, B. G. Janesko, R. Gomperts, B. Mennucci, H. P. Hratchian, J. V. Ortiz, A. F. Izmaylov, J. L. Sonnenberg, D. Williams-Young, F. Ding, F. Lipparini, F. Egidi, J. Goings, B. Peng, A. Petrone, T. Henderson, D. Ranasinghe, V. G. Zakrzewski, J. Gao, N. Rega, G. Zheng, W. Liang, M. Hada, M. Ehara, K. Toyota, R. Fukuda, J. Hasegawa, M. Ishida, T. Nakajima, Y. Honda, O. Kitao, H. Nakai, T. Vreven, K. Throssell, J. A. Montgomery, Jr., J. E. Peralta, F. Ogliaro, M. J. Bearpark, J. J. Heyd, E. N. Brothers, K. N. Kudin, V. N. Staroverov, T. A. Keith, R. Kobayashi, J. Normand, K. Raghavachari, A. P. Rendell, J. C. Burant, S. S. Iyengar, J. Tomasi, M. Cossi, J. M. Millam, M. Klene, C. Adamo, R. Cammi, J. W. Ochterski, R. L. Martin, K. Morokuma, O. Farkas, J. B. Foresman, and D. J. Fox, Gaussian, Inc., Wallingford CT, 2016.

[6] a) C. T. Lee, W. T. Yang, R. G. Parr, Phys. Rev. B, 1988, 37, 785-789. b) A. D. Becke, Phys. Rev. A, 1988, 38, 3098-3100

[7] Y. Zhao and D. G. Truhlar, "The M06 suite of density functionals for main group thermochemistry, thermochemical kinetics, noncovalent interactions, excited states, and transition elements: two new functionals and systematic testing of four M06-class functionals and 12 other functionals," *Theor. Chem. Acc.*, **120** (2008) 215-41. DOI: 10.1007/s00214-007-0310-x

[8] R. Ditchfield, W. J. Hehre, and J. A. Pople, "Self-Consistent Molecular Orbital Methods. 9. Extended Gaussian-type basis for molecular-orbital studies of organic molecules," *J. Chem. Phys.*, **54** (1971) 724. DOI: 10.1063/1.1674902

[9] T. H. Dunning Jr., "Gaussian basis sets for use in correlated molecular calculations. I. The atoms boron through neon and hydrogen," *J. Chem. Phys.*, **90** (1989) 1007-23. DOI: 10.1063/1.456153

[10] D. Geuenich, K. Hess, F. Köhler, R. Herges, Chem. Rev. 2005, 105, 3758–3772

[11] Neese, F. *The ORCA program system Wiley Interdiscip. Rev.: Comput. Mol. Sci.*, **2012**, *2*, *1*, 73–78 (DOI: 10.1002/wcms.81)

2. Experimental section

All solvents (MeOH, Ethyl Acetate, $CHCl_3$, n-hexane, toluene, acetone, water) if not indicated differently were used without purification. CH_2Cl_2 was distilled over CaH_2. Chloroform-*d* was prepared directly before using by passing through a basic alumina column. All reactions were performed under an inert atmosphere unless mentioned otherwise.

2.1 Experimental procedures

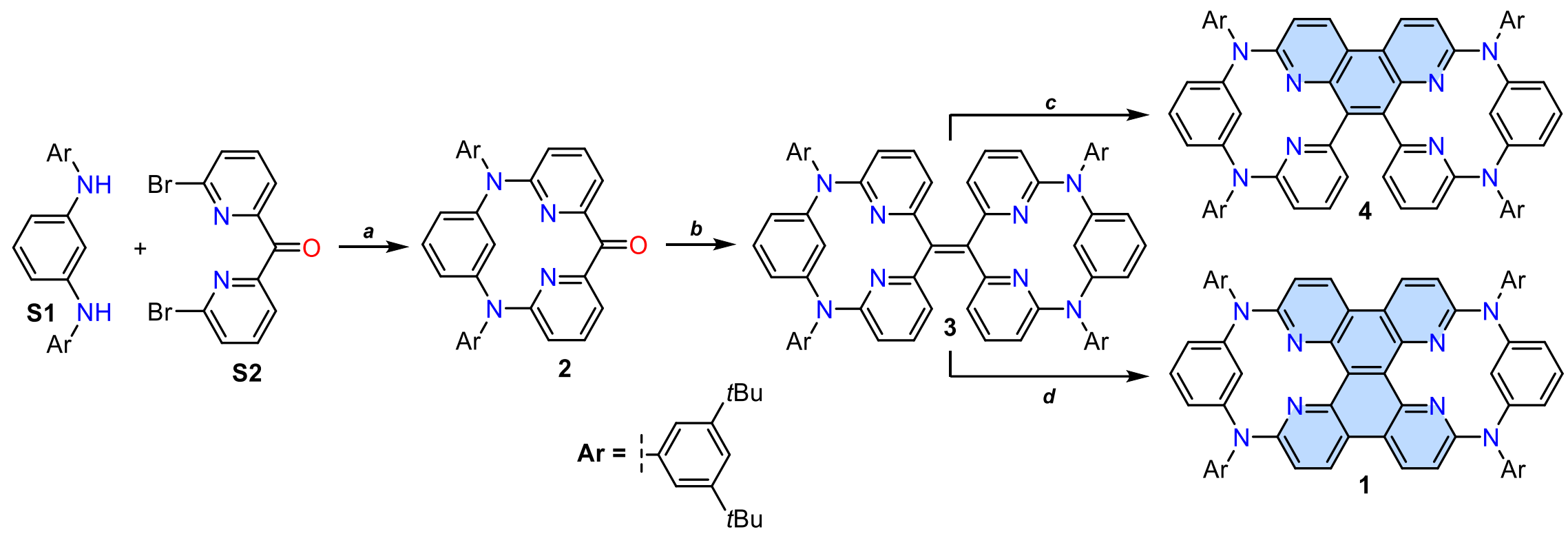

Scheme S1. Synthetic approach applied. *Conditions:* a) $Pd_2(dba)_3$, RuPhos, K_3PO_4, Toluene, reflux, 24 h; b) PBr_3, *o*-dichlorobenzene, reflux, 14 h ; c) *hv* (370 nm), O_2, EtOAc, 2 h; d) DDQ, Me_3SO_3H, DCE, reflux, 24 h.

Compound S2 was prepared according to Mayor et al. procedure.[12]

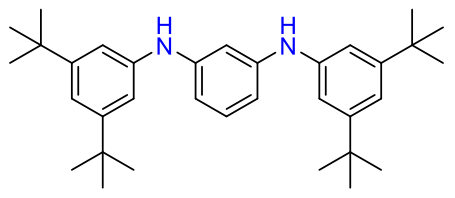

Compound S1. An oven dried Schlenk tube was loaded with benzene-1,3-diamine (1eq, 3.71 mmol), $Pd_2(dba)_3$ (0.02 eq, 74.3 µmol), *rac*BINAP (0.04 eq, 149 µmol), bromo-3,5-di-tert-butylbenzene (2.0 eq, 7.43 mmol) and NaO*t*Bu (2 eq, 7.43 mmol) and kept in vacuum overnight to dry. Degassed dry toluene 75 ml was added to a Schlenk tube and was refluxed for 24 hours with a heat-on block. After reaction completion, it was allowed to cool down and filtered through a silica plug and washed using ethyl acetate. The solution collected was concentrated under reduced pressure and purified by column chromatography in silica using hexane and ethyl acetate (5 %). Compound **2** was obtained as an off-white solid with 50 % yield. **^{1}H NMR (400 MHz, CDCl₃)** δ 7.11 (t, *J* = 8.0 Hz, 1H), 7.02 (t, *J* = 1.7 Hz, 2H), 6.96 (d, *J* = 1.7 Hz, 4H), 6.67 (d, *J* = 2.2 Hz, 1H), 6.58 (dd, *J* = 8.0, 2.2 Hz, 2H), 1.28 (s, 36H). **^{13}C NMR (101 MHz, $CDCl_3$)** δ 151.9, 145.6, 141.6, 130.2, 116.2, 114.5, 108.3, 104.0, 35.0, 31.6. **HRMS (m/z)**: 485.3870 $[M+H]^+$ (theor. calc. 485.3890 for $C_{34}H_{48}N_2$+H).

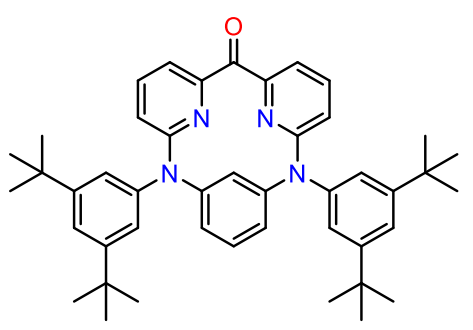

Compound 2. An oven dried Schlenk tube was loaded with **S1** (0.15 mmol), $Pd_2(dba)_3$ (0.15 eq., 22 µmol), RuPhos (0.60 eq., 90 µmol), **S2** (0.15 mmol) and K_3PO_4 (10 eq., 1.5 mmol) and kept in vacuum overnight to dry. Degassed dry toluene 15 ml was added to a Schlenk tube and was refluxed for 24 hours with a heat-on block. After reaction completion, it was allowed to cool down and filtered through a silica plug and washed using ethyl acetate. The solution collected was concentrated under reduced pressure and purified by column chromatography in silica using hexane and ethyl acetate (5 %). Compound **4** was obtained as a yellow solid with 47 % yield. **^{1}H NMR (400 MHz, $CDCl_3$)** δ 8.59 (t, *J* = 2.2 Hz, 1H), 7.55 – 7.48 (m, 4H), 7.40 (t, *J* = 1.7 Hz, 2H), 7.26 (d, J = 1.7 Hz, 4H), 6.95 (t, *J* = 8.1 Hz, 1H), 6.68 (dd, *J* = 5.5, 3.7 Hz, 2H), 6.37 (dd, *J* = 8.1, 2.2 Hz, 2H), 1.33 (s, 36H). **^{13}C NMR (101 MHz, $CDCl_3$)** δ 189.7, 157.5, 153.1, 152.8, 143.0, 142.9, 137.9, 127.0, 125.4, 123.0, 120.8, 117.1, 116.1, 114.2, 35.2, 31.6. **HRMS (m/z)**: 665.4209 $[M+H]^+$ (theor. calc. 665.4219 for $C_{45}H_{52}N_4O$+H **UV-Vis (cyclohexane)**: 276, 402 nm, **Extinction (cyclohexane)**: $3.7 \cdot 10^4$ $M^{-1}cm^{-1}$ (276 nm), **Emission (cyclohexane)**: 614 nm.

[12] M. Lindner, M. Valàsek, J. Homberg, K. Edelmann, L. Gerhard, W. Wulfhekel, O. Fuhr, T. Wächter, M. Zharnikov, V. Kolivoska, L. Pospìsil, G. Mészàros, M. Hromadovà, and M. Mayor, Chem. Eur J., 2016, 22, 1321813235.

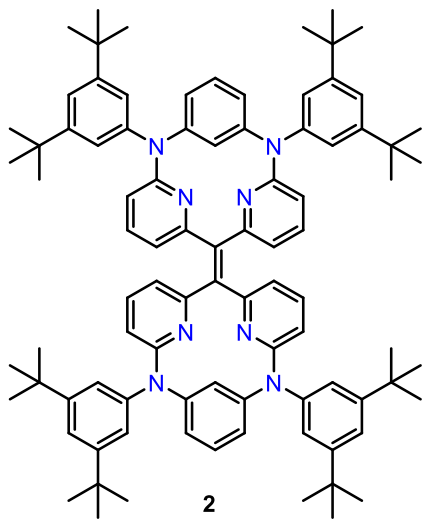

2

Compound 3. An oven dried Schlenk tube was charged with macrocycle **1** (1 eq, 30 µmol) and dried overnight. Dry degassed *o*-dichlorobenzene (3 ml) was added to the schlenk tube. Then PBr_3 (10 eq, 0.30 mmol) was added dropwise into the Schlenk tube at room temperature. The solution was refluxed for 14h at 180 °C with a heat-on block. The reaction was allowed to cool down and quenched by adding water (5 ml). The organic layer was extracted with dichloromethane and dried with Na_2SO_4. It was evaporated under reduced pressure. The crude was purified by column chromatography in alumina using hexane and dichloromethane (50 %). Compound **5** was obtained as a white solid with 65 % yield. **^{1}H NMR (300 MHz, $CDCl_3$)** δ 7.67 (t, J = 2.2 Hz, 1H), 7.31 – 7.26 (m, 6H), 7.11 (dd, *J* = 8.3, 7.5 Hz, 2H), 6.97 (t, J = 8.1 Hz, 1H), 6.78 (dd, J = 7.4, 0.6 Hz, 2H), 6.46 (dd, J = 8.1, 2.2 Hz, 2H), 6.41 (d, J = 8.3 Hz, 2H), 1.33 (s, 36H). **^{13}C NMR (101 MHz, $CDCl_3$)** δ 158.4, 156.0, 152.4, 144.3, 143.8, 140.5, 136.7, 127.8, 124.2, 122.3, 119.6, 119.3, 116.4, 110.5, 35.1, 31.6. **HRMS (m/z)**: 1297.8384 $[M+H]^+$ (theor. calc. 1297.8462 for $C_{90}H_{104}N_8$+H), **UV-Vis (cyclohexane)**: 284, 370 nm, **Extinction (cyclohexane)**: 6.8·10^4 $M^{-1}cm^{-1}$ (284 nm), **Emission (cyclohexane)**: 459 nm.

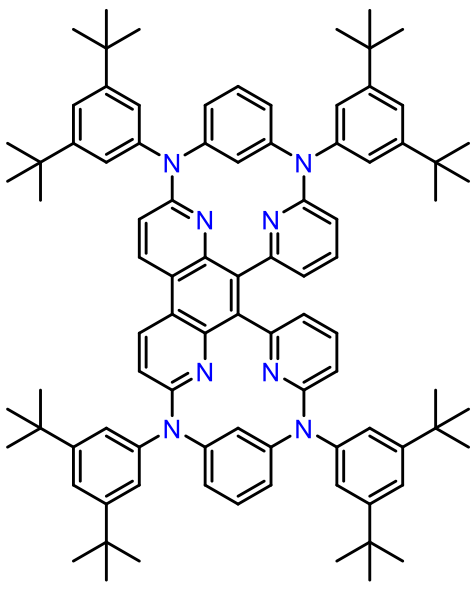

Compound 4. In an oven-dried round bottom flask was charged with macrocycle **2** (1 eq., 15 µmol) was dissolved in ethyl acetate (30 ml, not degassed). The solution was stirred for 2 hours under UV 370 nm Kessil lamp generation 2 (25% power). The solution was concentrated under reduced pressure. The crude was purified by column chromatography in alumina using hexane and dichloromethane (20-50 %). Compound **6** was obtained as a yellow solid with 75 % yield. **^{1}H NMR (600 MHz,CD_2Cl_2)** δ 8.54 (t, *J* = 2.2 Hz, 1H), 8.38 (d, *J* = 9.2 Hz, 1H), 7.46 (t, *J* = 1.8 Hz, 1H), 7.36 – 7.31 (m, 5H), 7.29 (t, *J* = 1.7 Hz, 1H), 7.00 (t, *J* = 8.0 Hz, 1H), 6.95 – 6.88 (m, 2H), 6.78 – 6.70 (m, 2H), 6.53 (ddd, *J* = 8.0, 2.2, 0.8 Hz, 1H), 1.36 (s, 18H), 1.34 (s, 18H).**^{13}C NMR (151 MHz, CD_2Cl_2)** δ 159.3, 157.8, 155.4, 153.2, 152.7, 145.7, 144.9, 144.4, 143.6, 142.8, 138.8, 137.5, 130.8, 128.6, 127.1, 124.4, 122.6, 121.5, 120.9, 120.4, 119.1, 118.2, 118.0, 113.6, 112.2, 35.4, 35.3, 31.6. **HRMS (m/z)**: 1295.8213 $[M+H]^+$ (theor. calc. 1295.8300 for $C_{90}H_{102}N_8$+H), **UV-Vis (cyclohexane)**: 293, 367 nm, **Extinction (cyclohexane)**: 7.0·10^4 $M^{-1}cm^{-1}$ (293 nm), **Emission (cyclohexane)**: 514 nm.

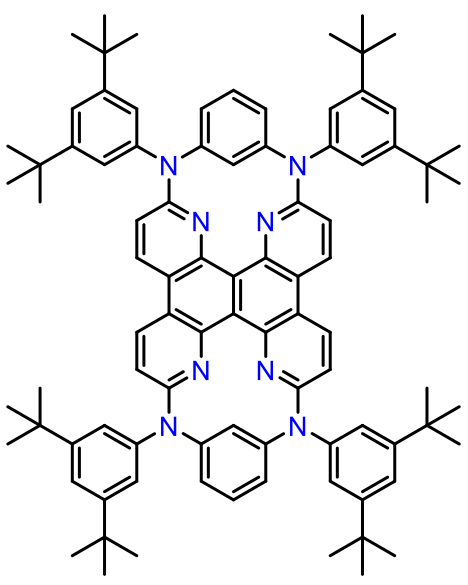

Compound 1. An oven dried Schlenk tube was charged with macrocycle **2** (1 eq, 30 µmol) and DDQ (2.5 eq. 75 µmol) were dried overnight. Dry degassed Dichloroethane (2 ml) was added to the Schlenk tube. Methanesulfonic acid (10 eq) was added drop-wise and the reaction was refluxed for 36h with a heat-on block. The completion of the reaction was monitored by TLC. The reaction was quenched by 2M NaOH solution and the organic layer was extracted with dichloromethane and dried with Na_2SO_4. It was evaporated under reduced pressure. The crude was purified by column chromatography in alumina using hexane and ethyl acetate (5 %). Compound **1** was obtained as an orange solid with 96 % yield. **^{1}H NMR (400 MHz, 10ul Et_3N in 0.6ml of $CDCl_3$)** δ 10.01 (t, *J* = 2.2 Hz, 1H), 8.32 (d, *J* = 9.2 Hz, 2H), 7.44 (t, *J* = 1.8 Hz, 2H), 7.31 (d, *J* = 1.8 Hz, 4H), 6.89 (t, *J* = 8.1 Hz, 1H), 6.78 (d, *J* = 9.1 Hz, 2H), 6.24 (dd, *J* = 8.1, 2.2 Hz, 2H), 1.34 (s, 36H). **^{13}C NMR (101 MHz, 10ul Et_3N in 0.6ml of $CDCl_3$) δ** 155.8, 153.2, 144.1, 143.4, 143.0, 131.7, 130.5, 127.0, 126.4, 124.0, 121.0, 120.9, 115.4, 112.1, 35.2, 31.6. **HRMS (m/z)**: 647.4129 $[M+2H]^{2+}$ (theor. calc. 647.4108 for $C_{90}H_{100}N_8$+2H), **UV-Vis (cyclohexane)**: 262, 308, 318, 362, 449, 478, 509, 513 nm, **Extinction (cyclohexane)**: 8.3·10^4 $M^{-1}cm^{-1}$ (262 nm), **Emission (cyclohexane)**: 517 nm.

3. NMR spectra

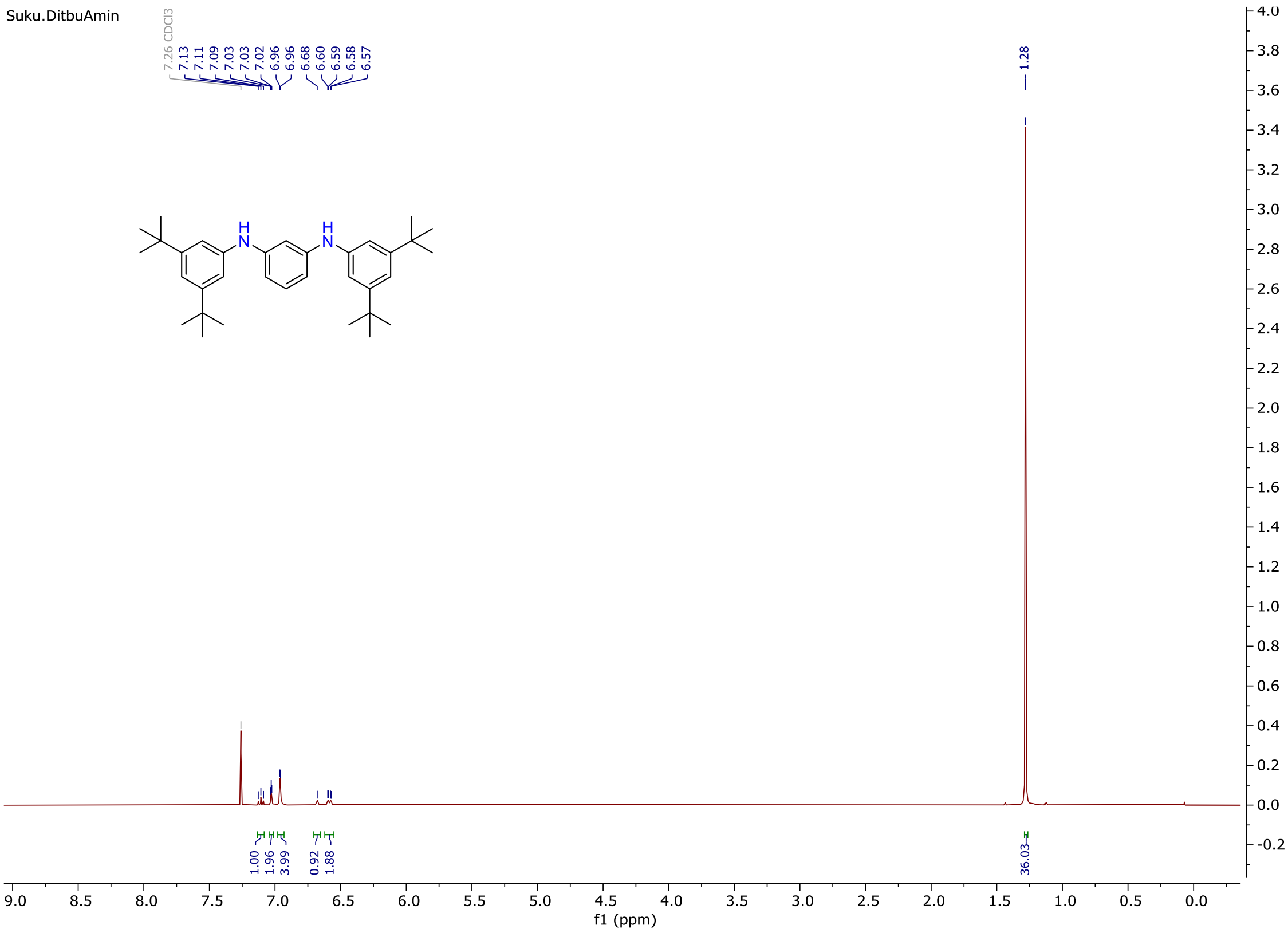

Figure S1. [1] H NMR spectrum of **S1** ($CDCl_3$, 298K, 400 MHz).

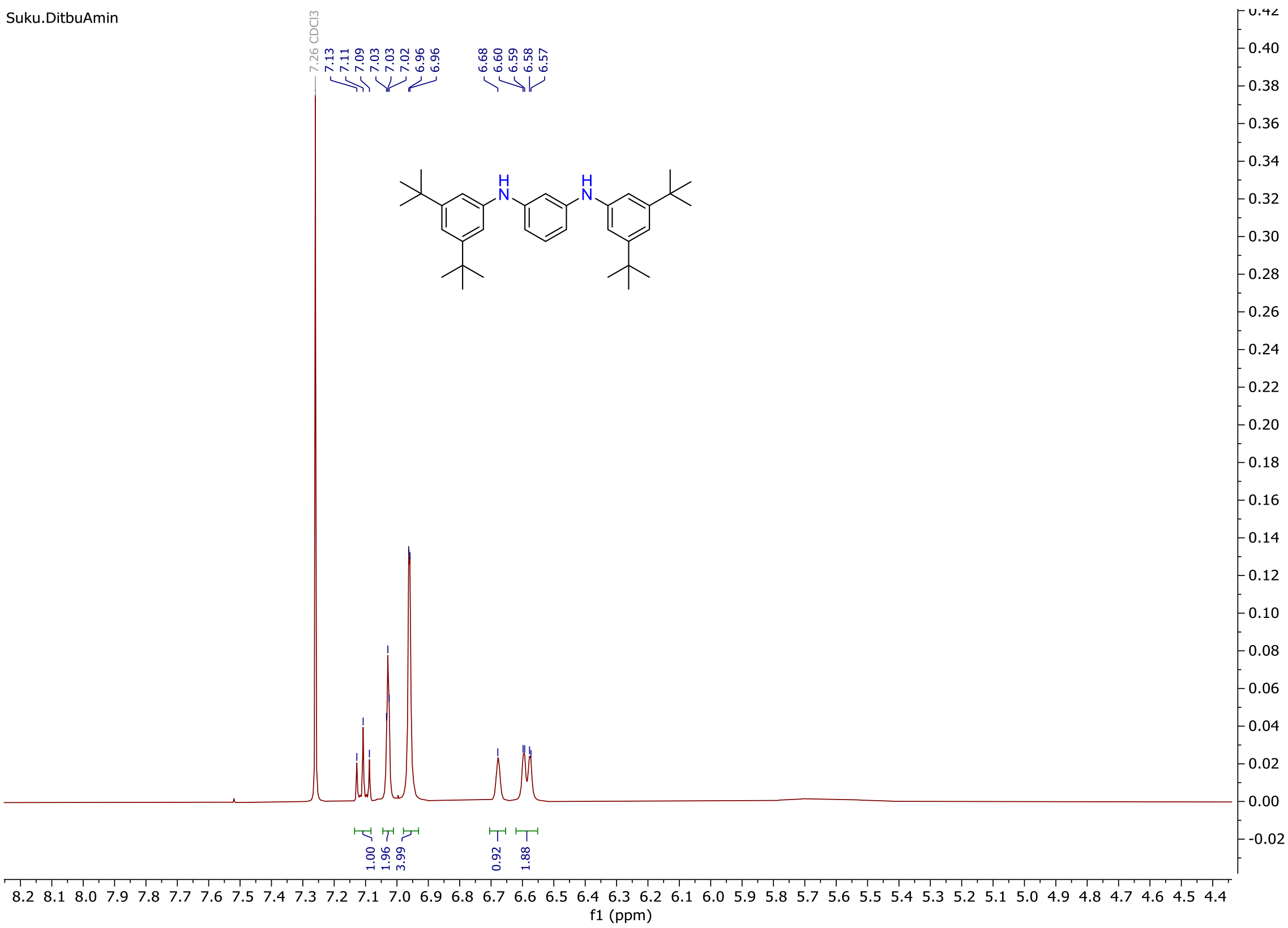

Figure S2. [1] H NMR spectrum (zoomed aromatic region) of **S1** ($CDCl_3$, 298K, 400 MHz).

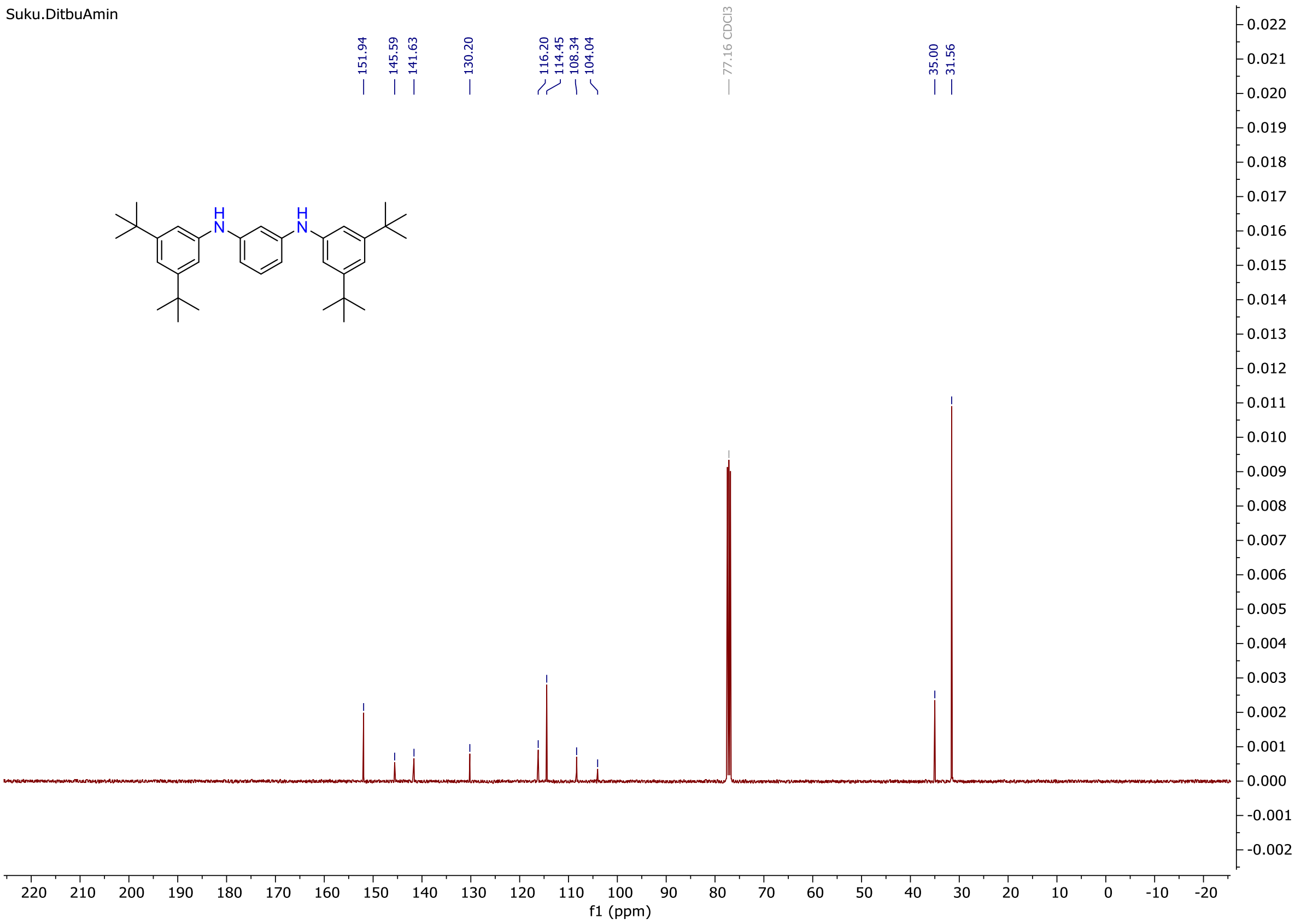

Figure S3. ^{13}C NMR spectrum of **S1** ($CDCl_3$, 298K, 101 MHz).

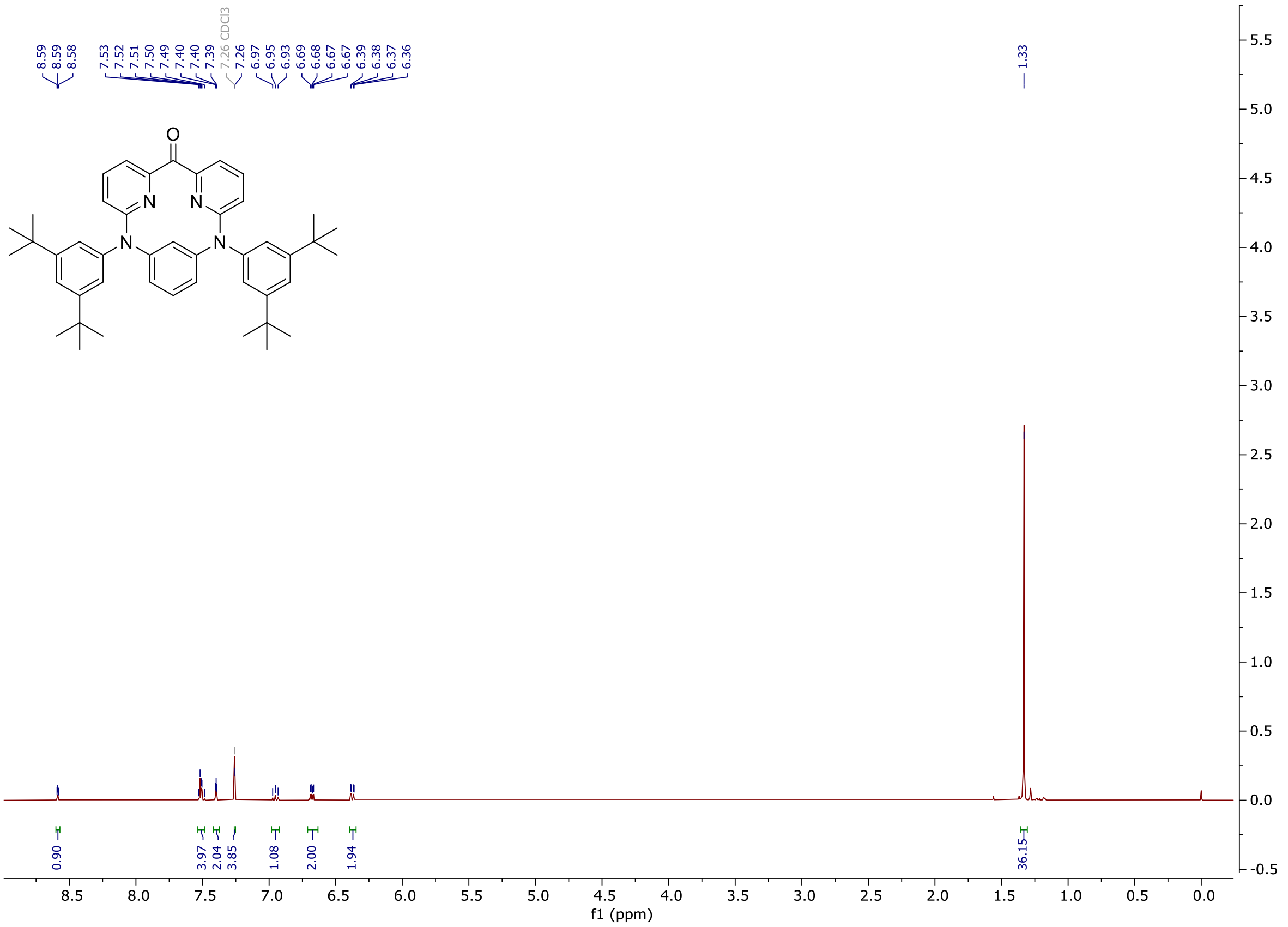

Figure S4. ^{1}H NMR spectrum of **2** ($CDCl_3$, 298K, 400 MHz).

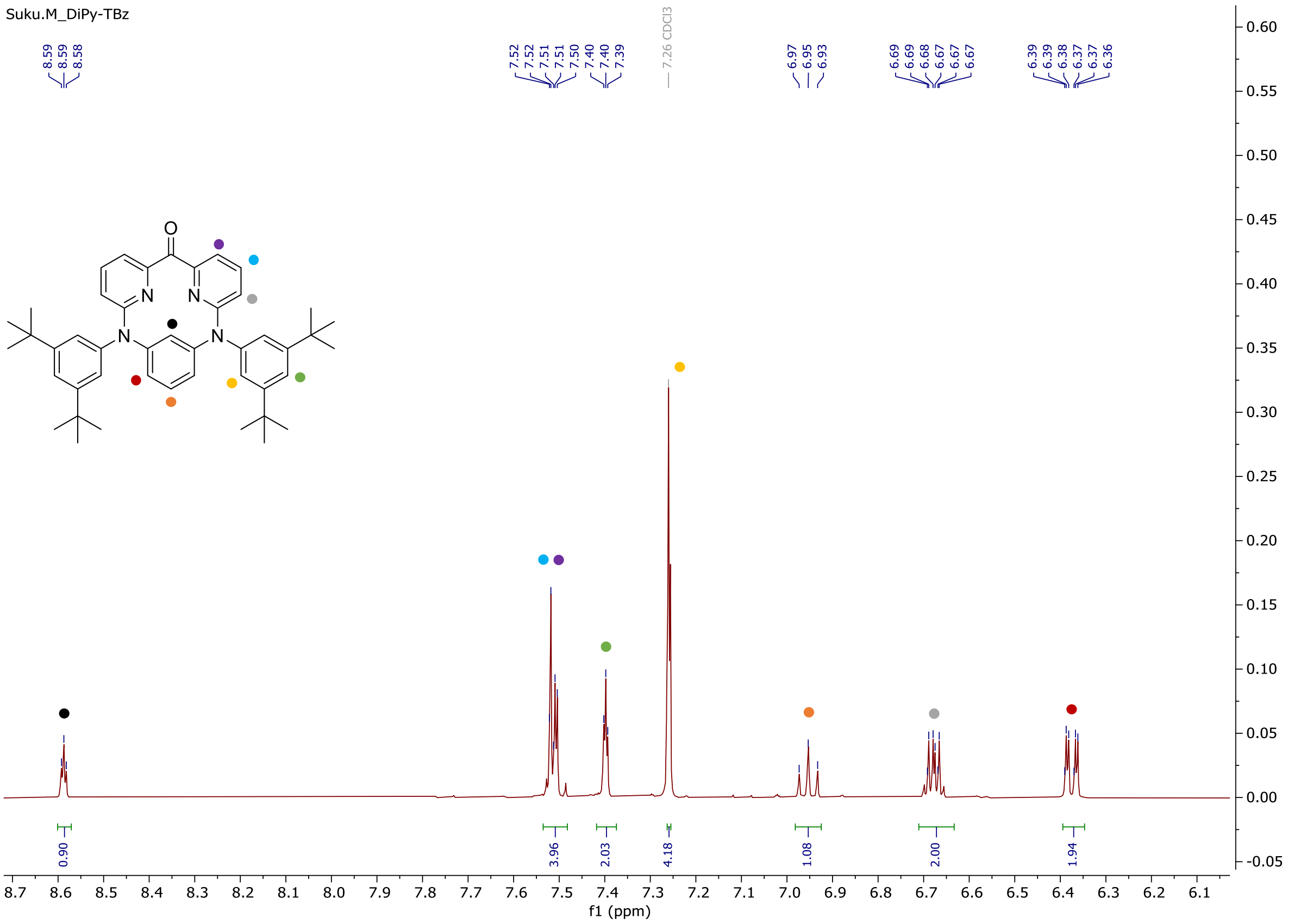

Figure S5. ^{1}H NMR spectrum (zoomed aromatic region) of **2** ($CDCl_3$, 298K, 400 MHz).

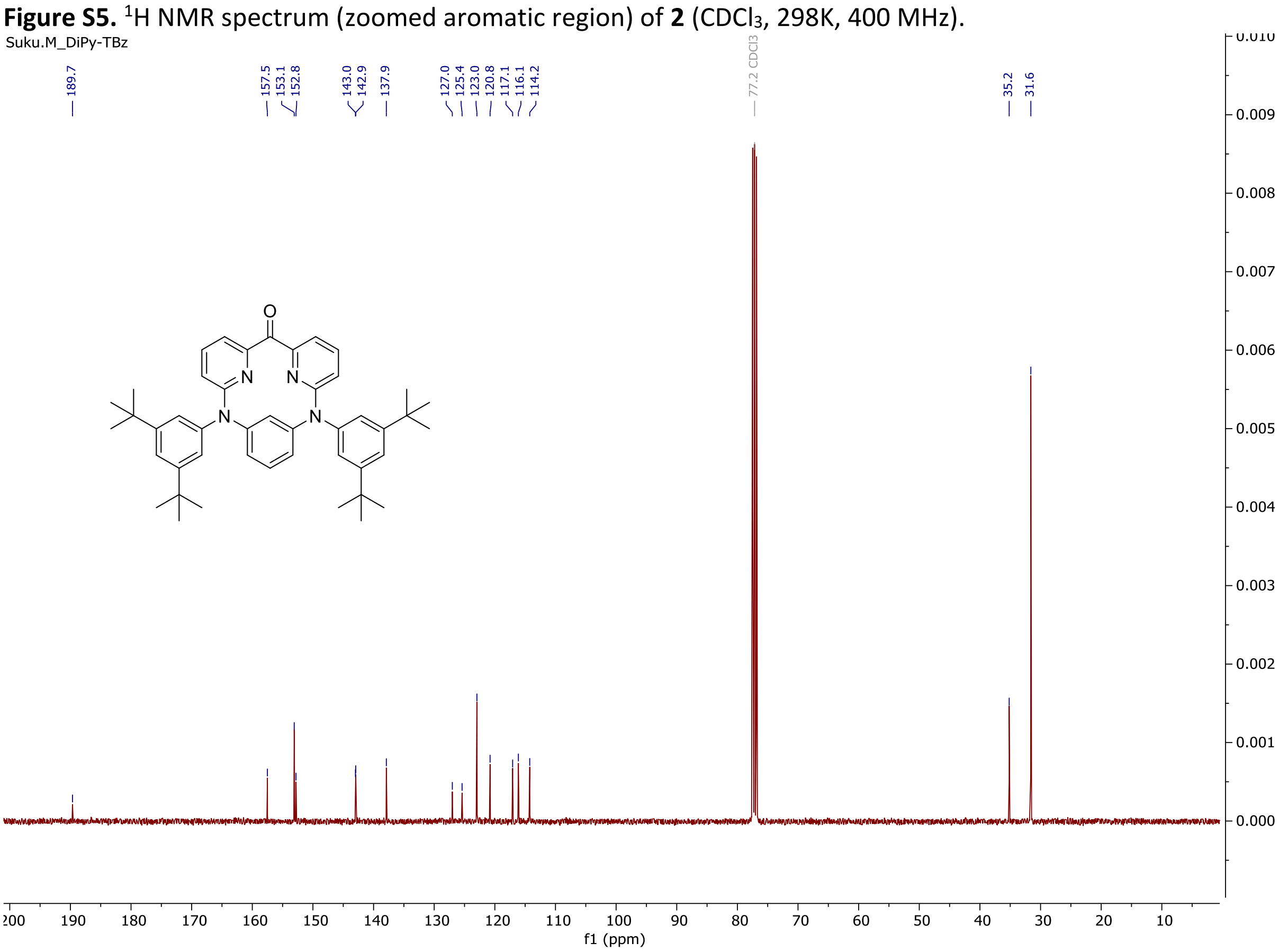

Figure S6. ^{13}C NMR spectrum of **2** ($CDCl_3$, 298K, 101MHz).

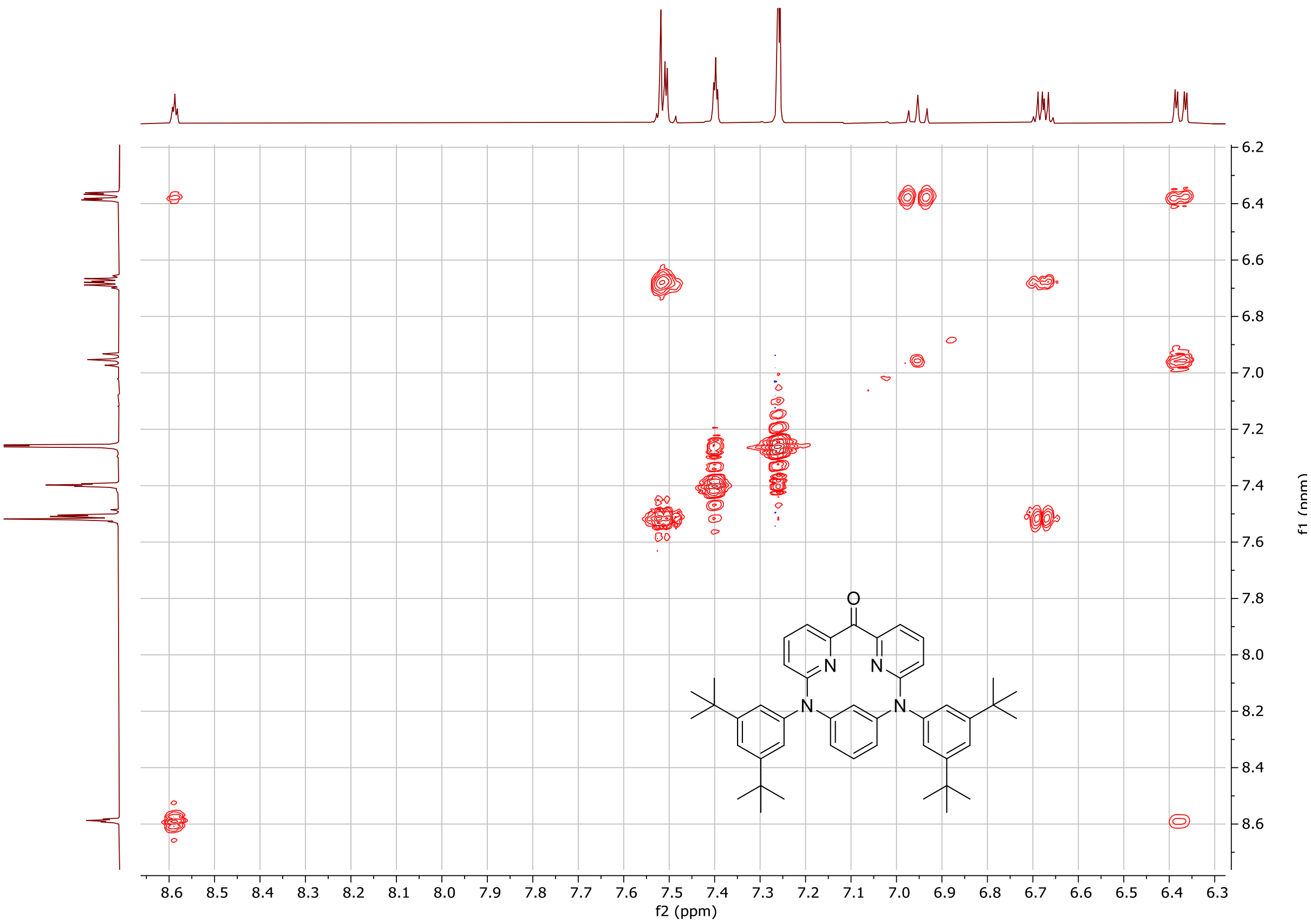

Figure S7. COSY NMR spectrum (zoomed aromatic region) of **2** ($CDCl_3$, 298K, 400 MHz).

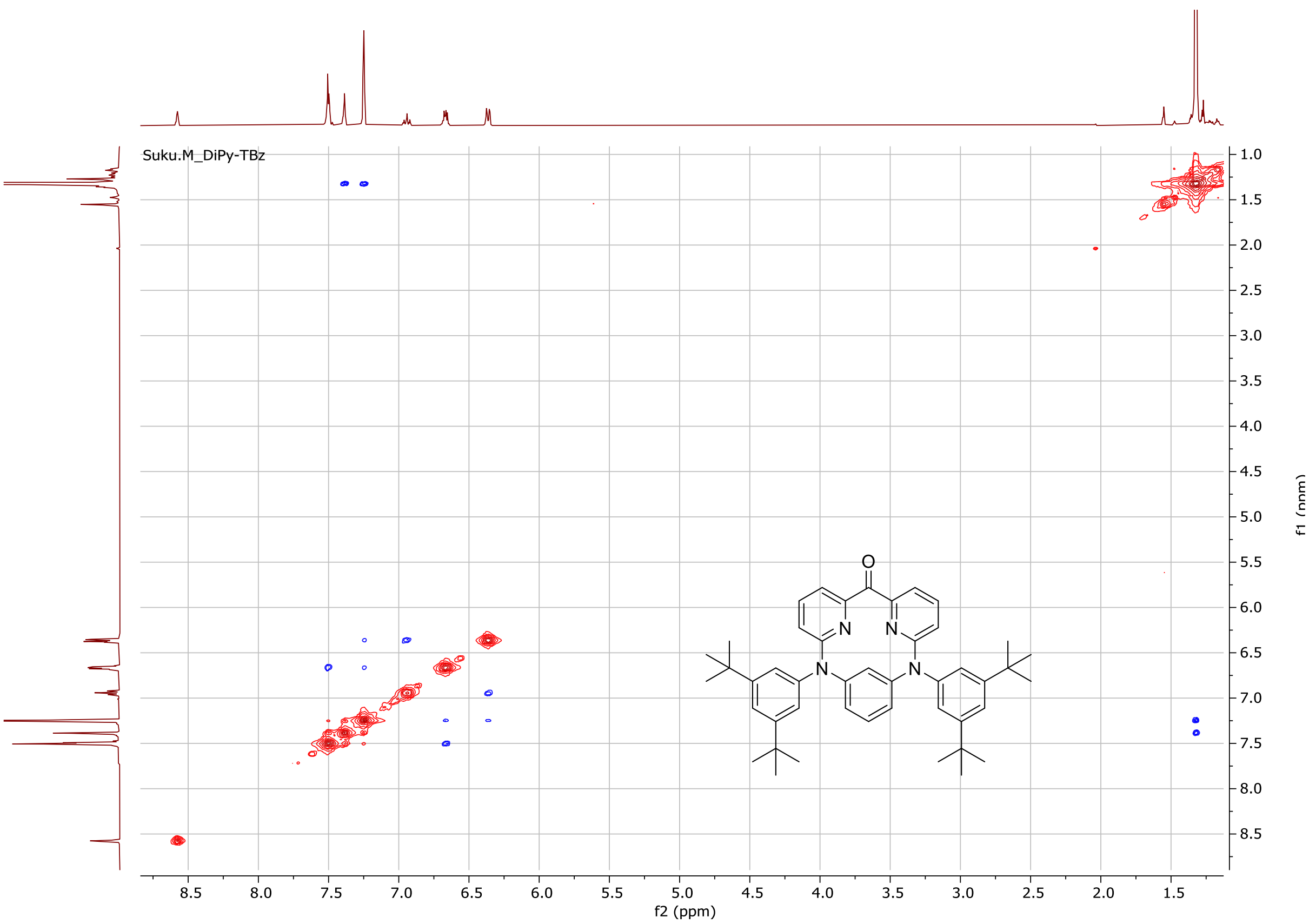

Figure S8. NOSEY NMR spectrum of **2** ($CDCl_3$, 298K, 400 MHz).

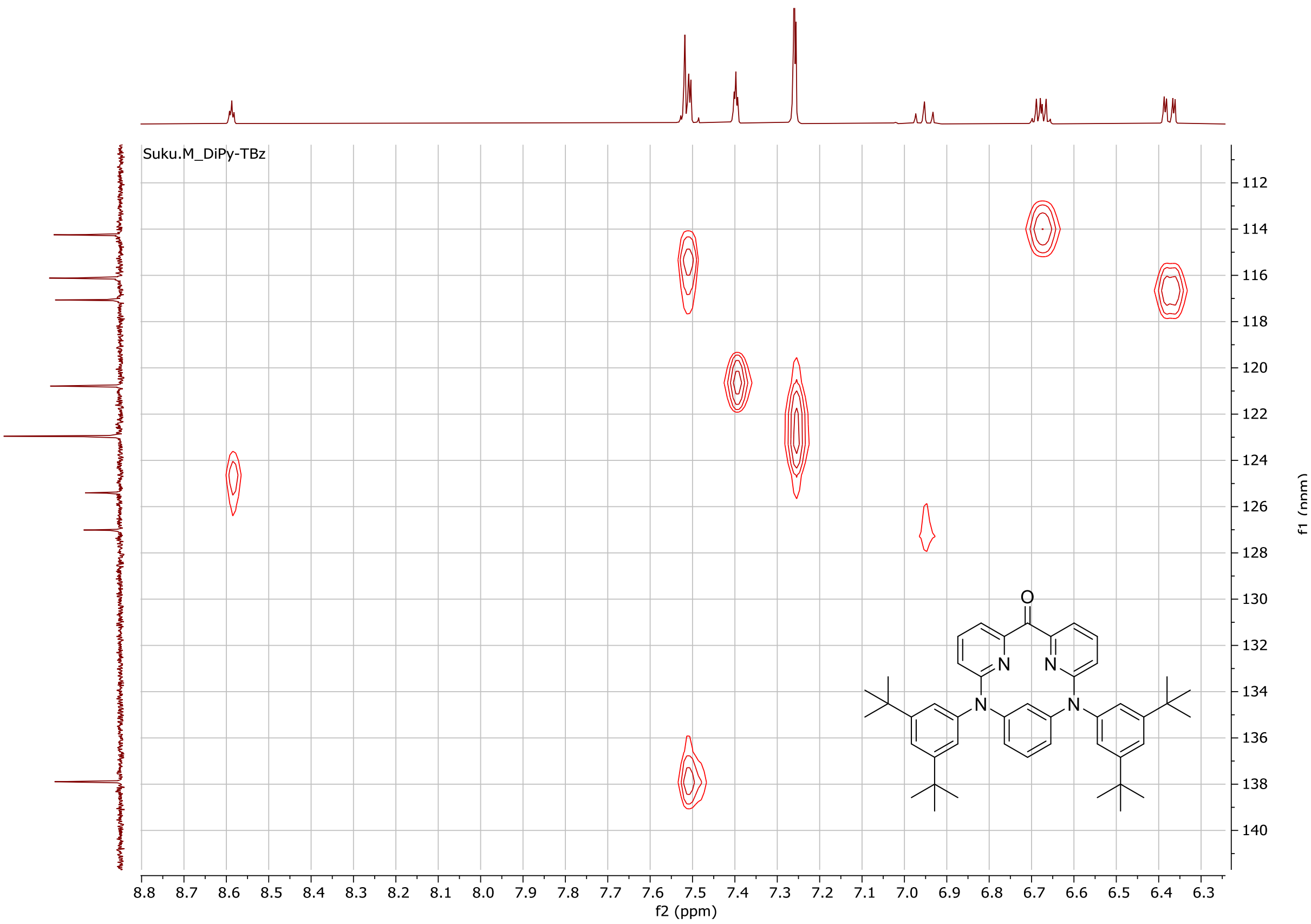

Figure S9. HSQC NMR spectrum (zoomed aromatic region) of **2** ($CDCl_3$, 298K, 400, 101 MHz).

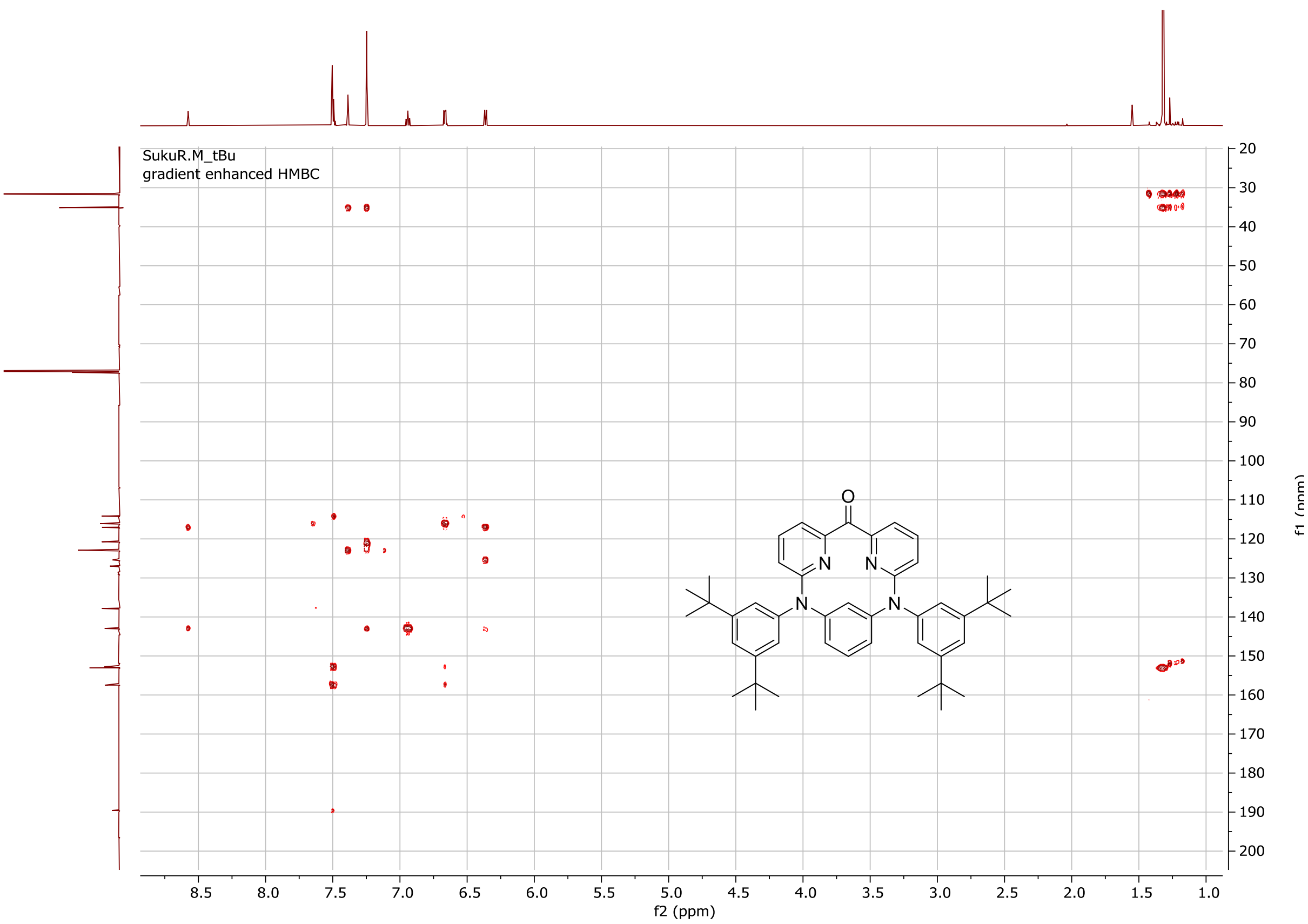

Figure S10. HMBC NMR spectrum of **2** ($CDCl_3$, 298K, 400, 101 MHz).

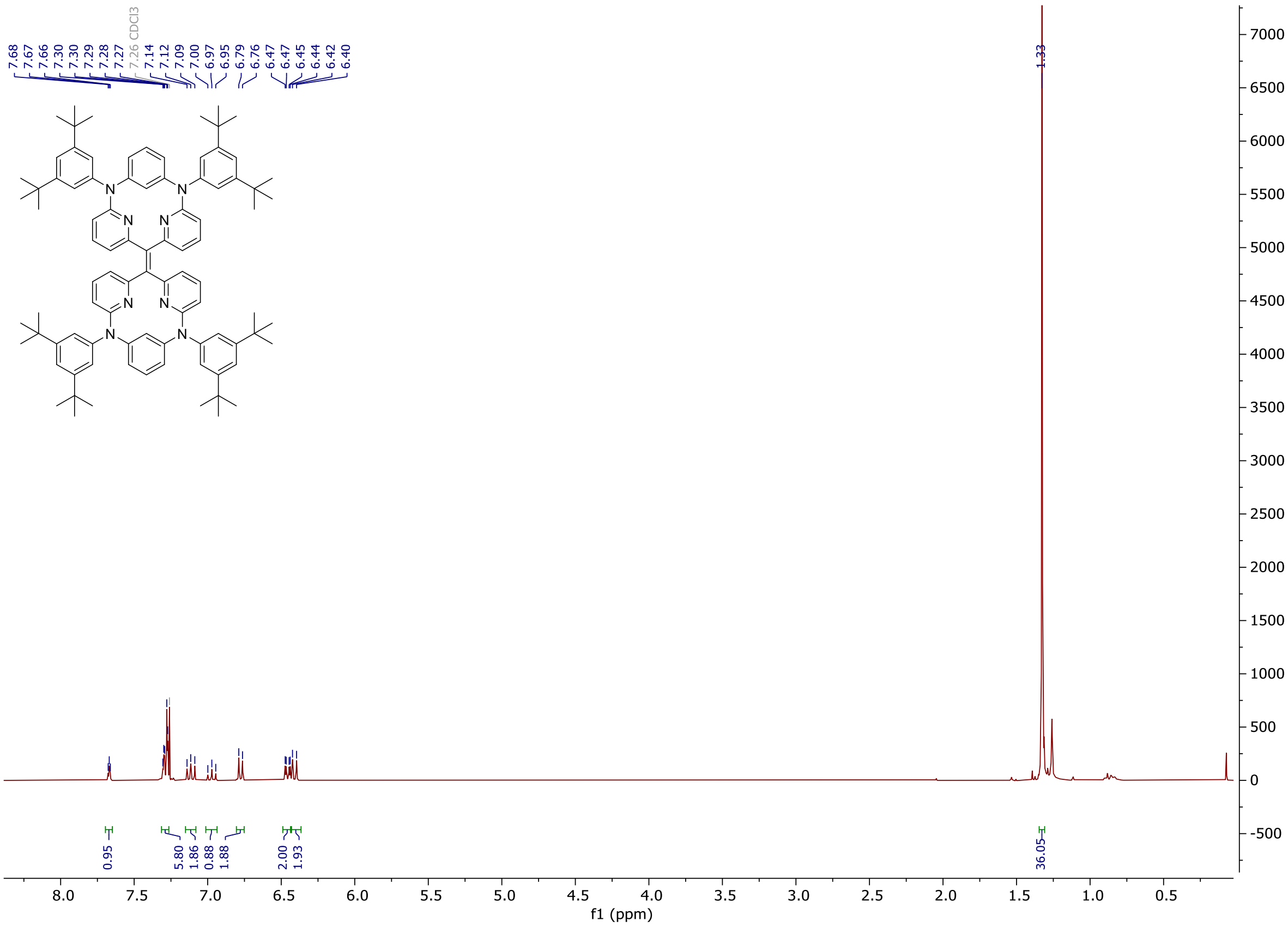

Figure S11. ^{1}H NMR spectrum of **3** ($CDCl_3$, 298K, 300 MHz).

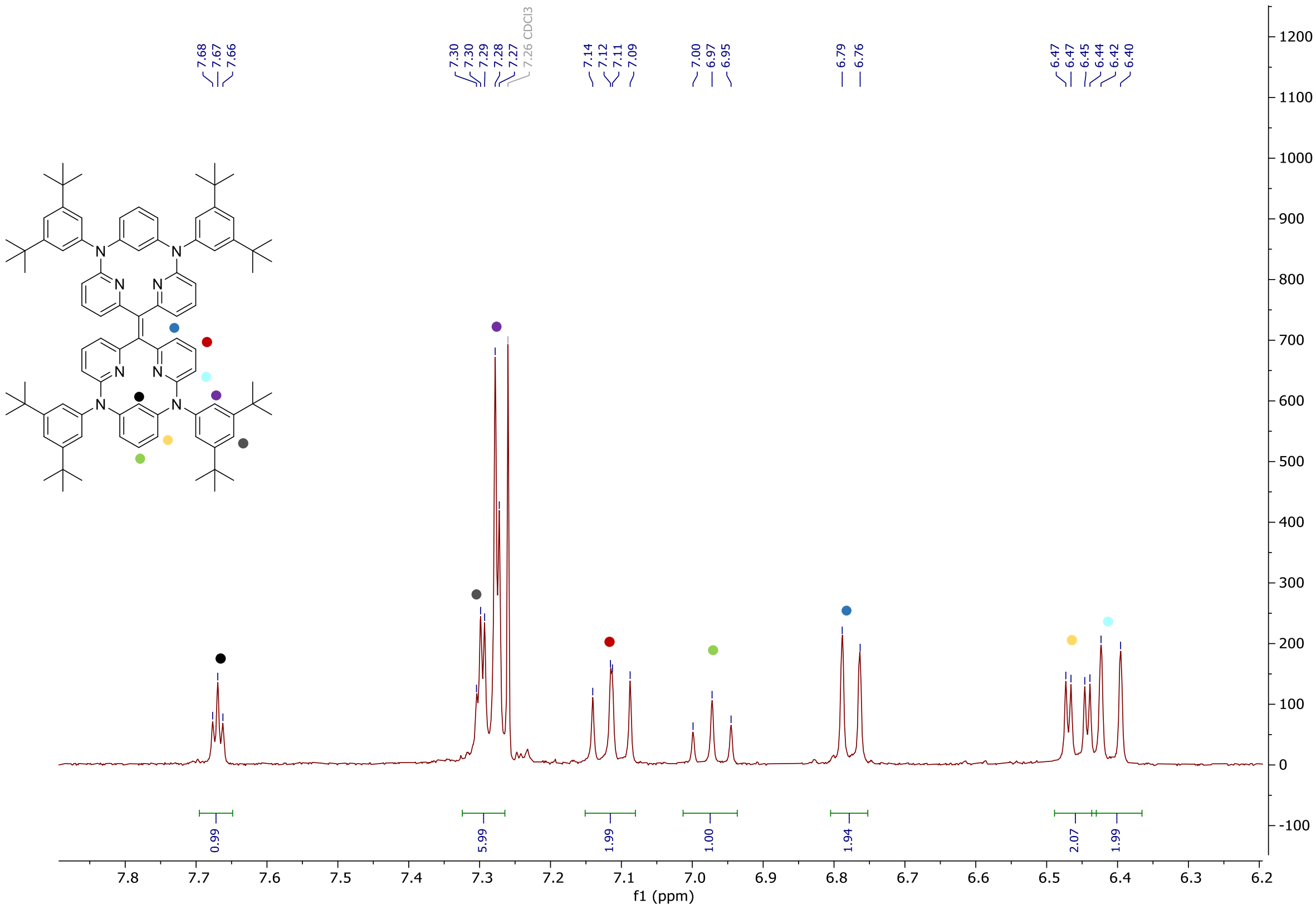

Figure S11_1. ^{1}H NMR spectrum (zoomed aromatic region) of **3** ($CDCl_3$, 298K, 300 MHz).

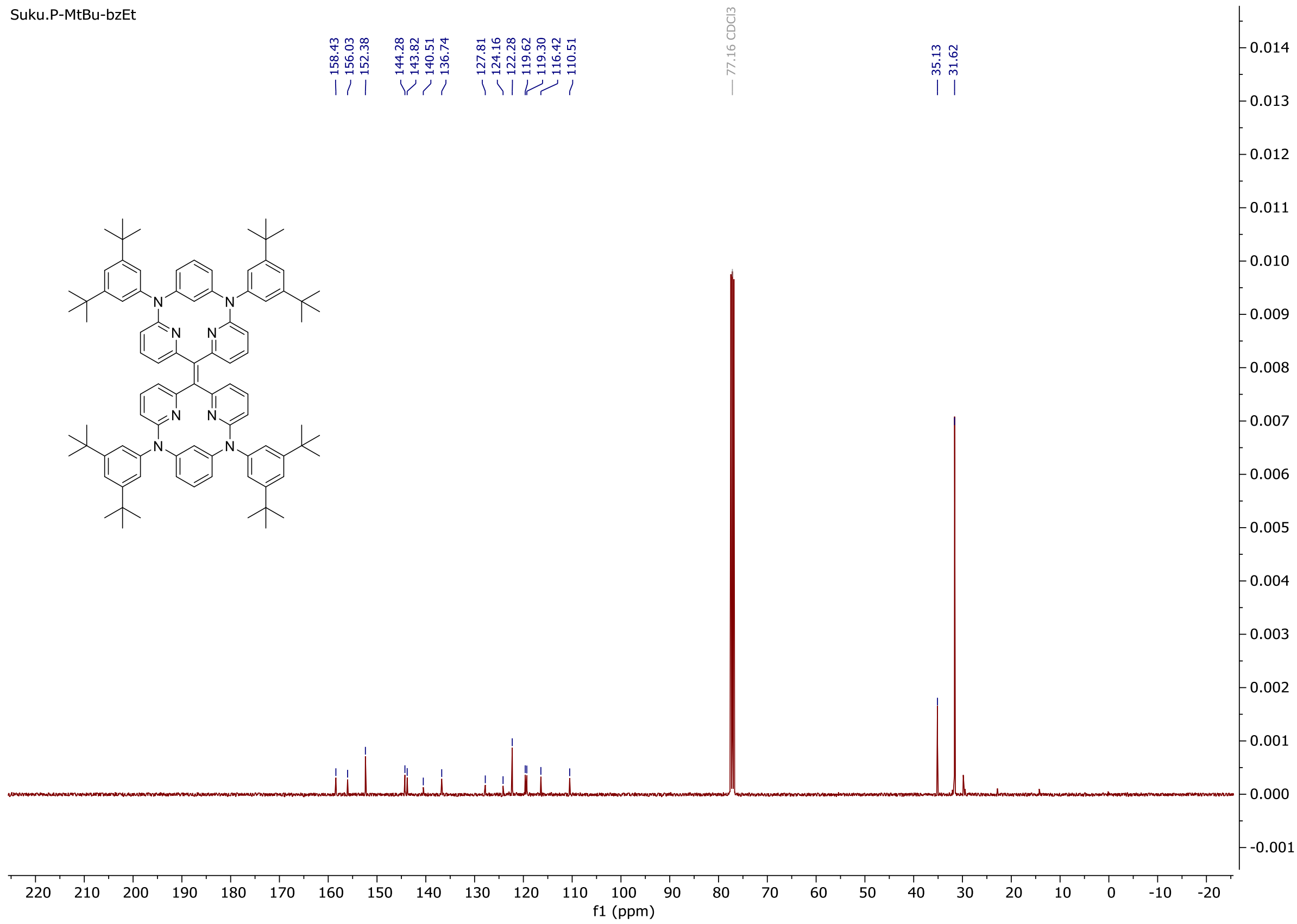

Figure S12. ^{13}C NMR spectrum of **3** ($CDCl_3$, 298K, 101MHz).

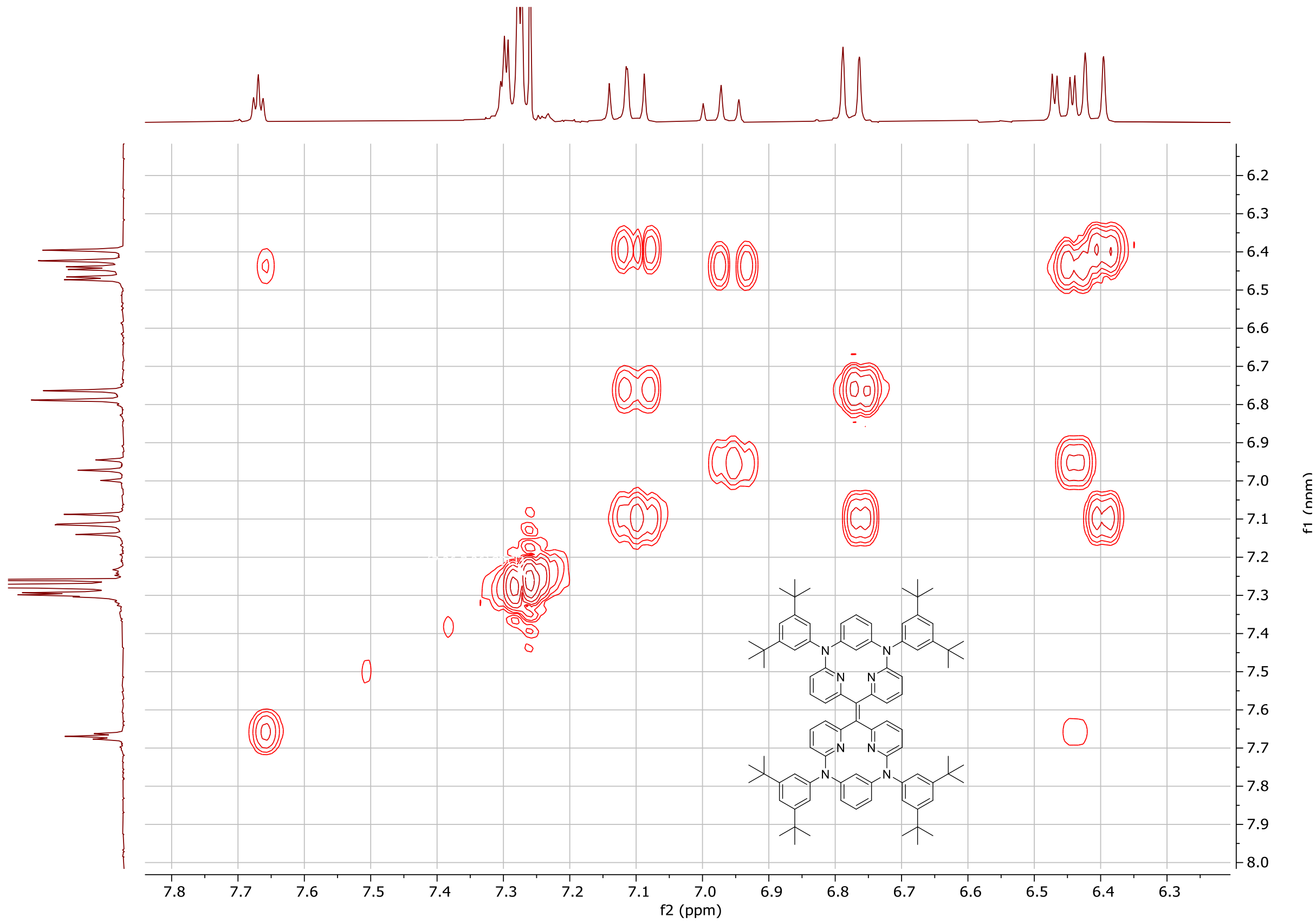

Figure S13. COSY NMR spectrum (zoomed aromatic region) of **3** ($CDCl_3$, 298K, 400 MHz).

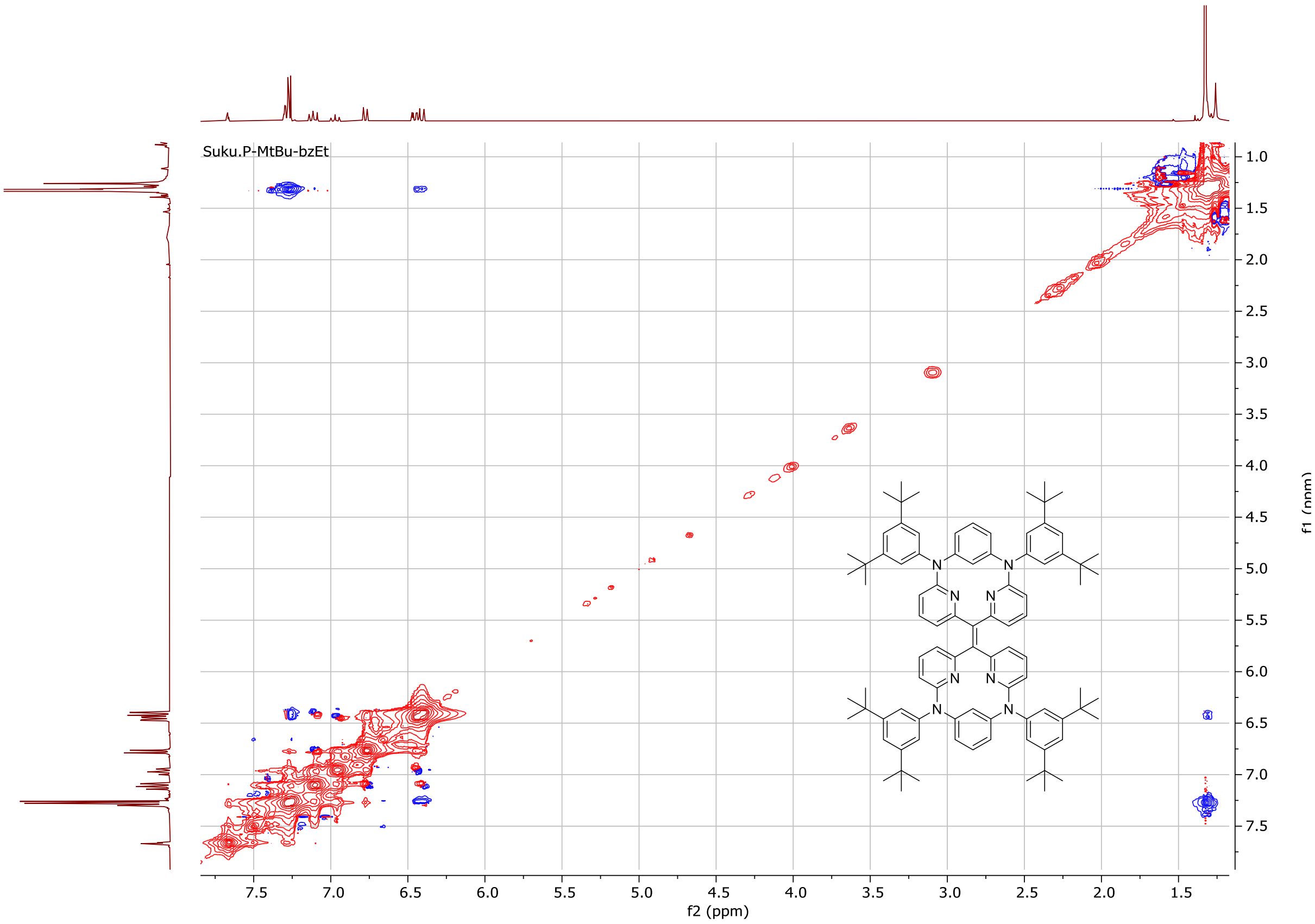

Figure S14. NOSEY NMR spectrum of **3** ($CDCl_3$, 298K, 400 MHz).

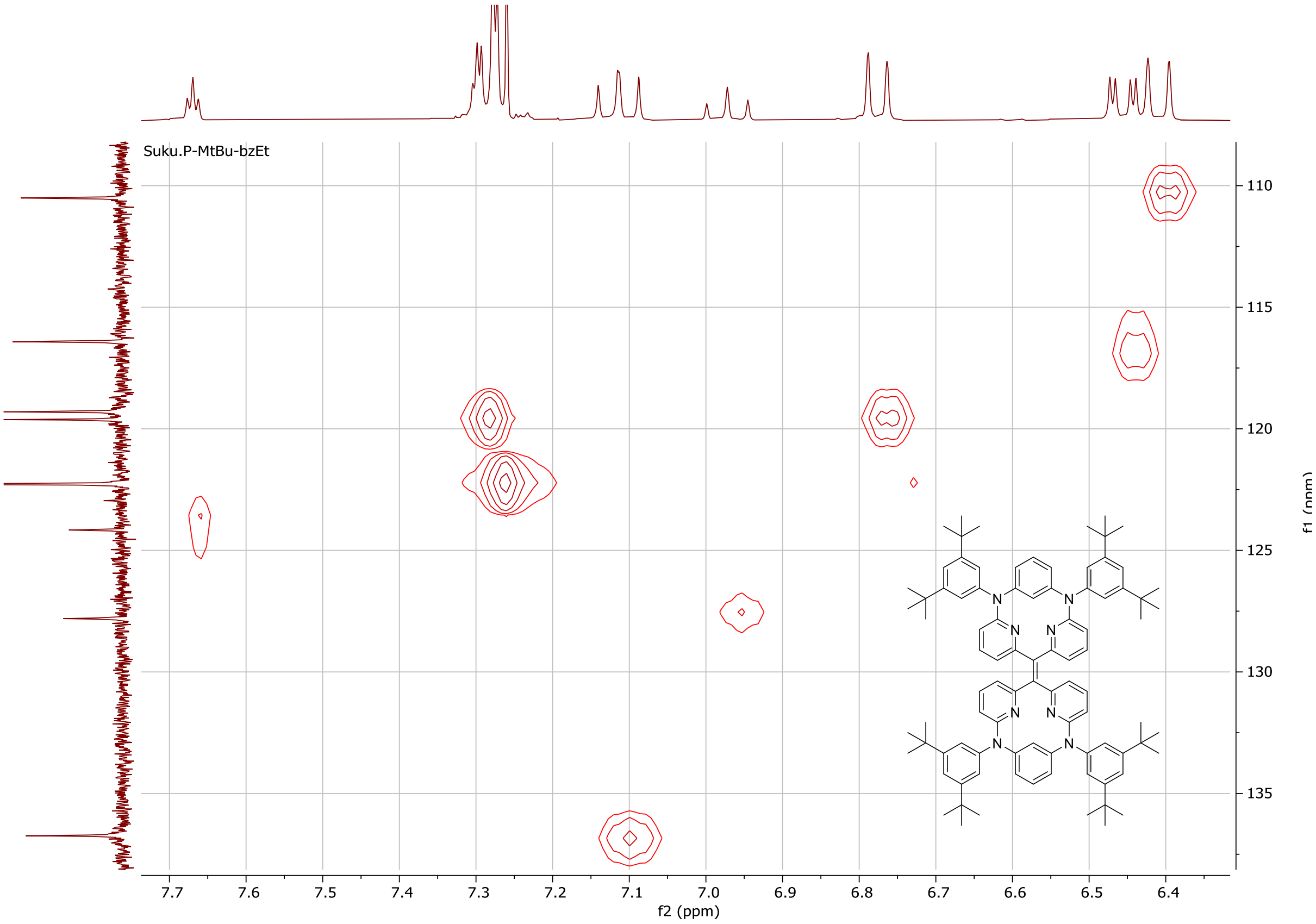

Figure S15. HSQC NMR spectrum (zoomed aromatic region) of **3** ($CDCl_3$, 298K, 400, 101 MHz).

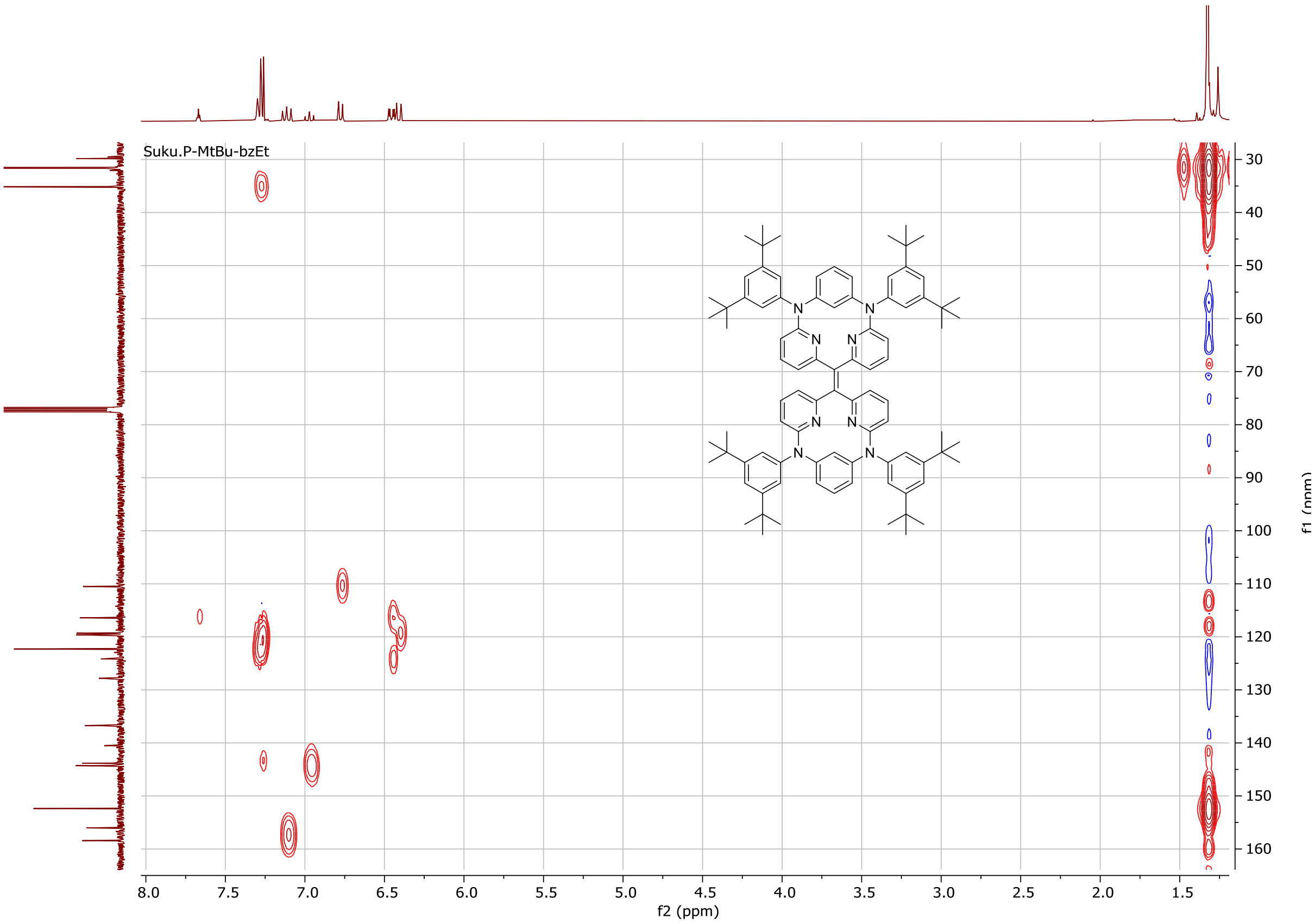

Figure S16. HMBC NMR spectrum of **3** ($CDCl_3$, 298K, 400, 101 MHz).

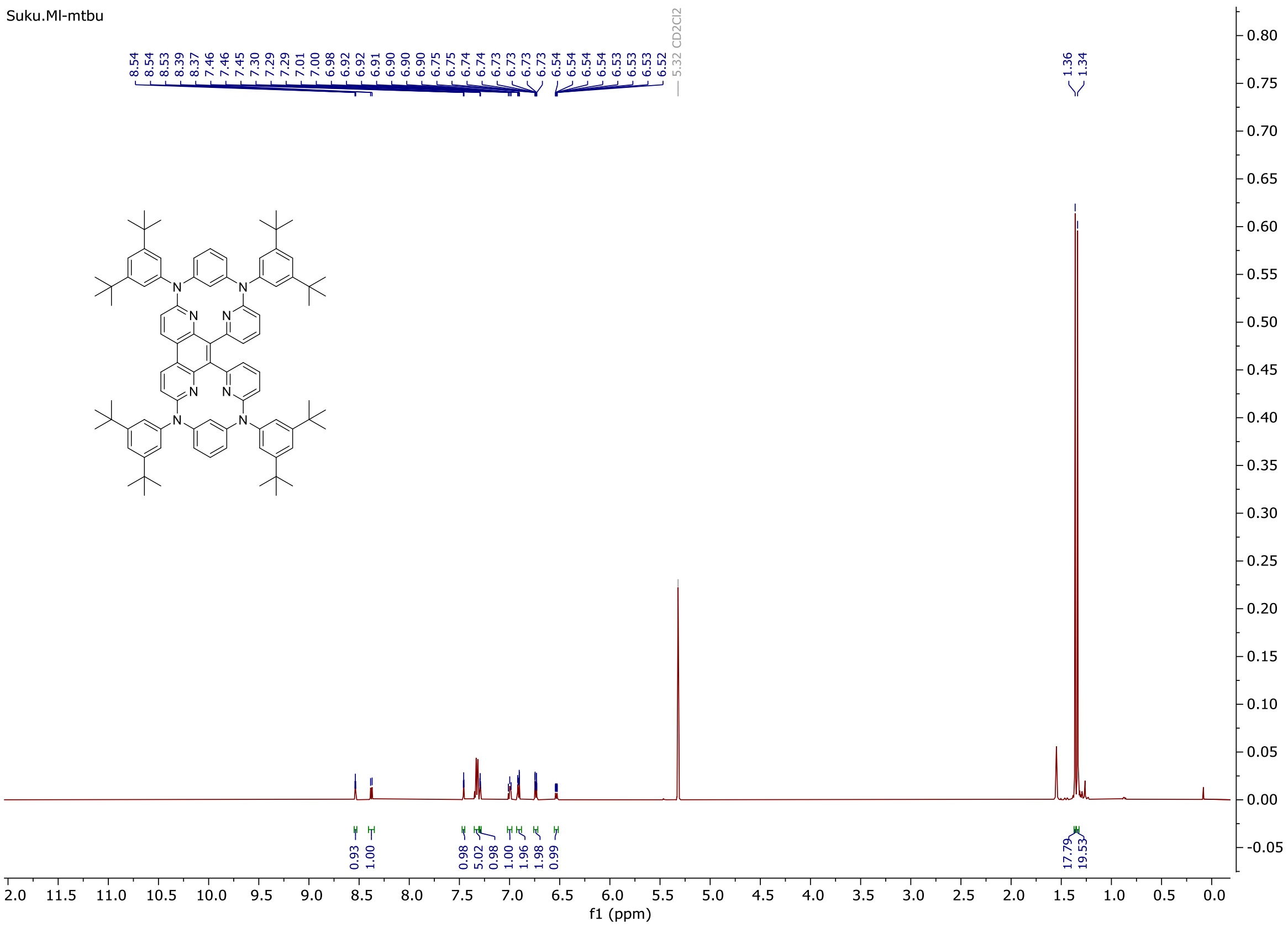

Figure S17. ^{1}H NMR spectrum of **4** (CD_2Cl_2, 298K, 600 MHz).

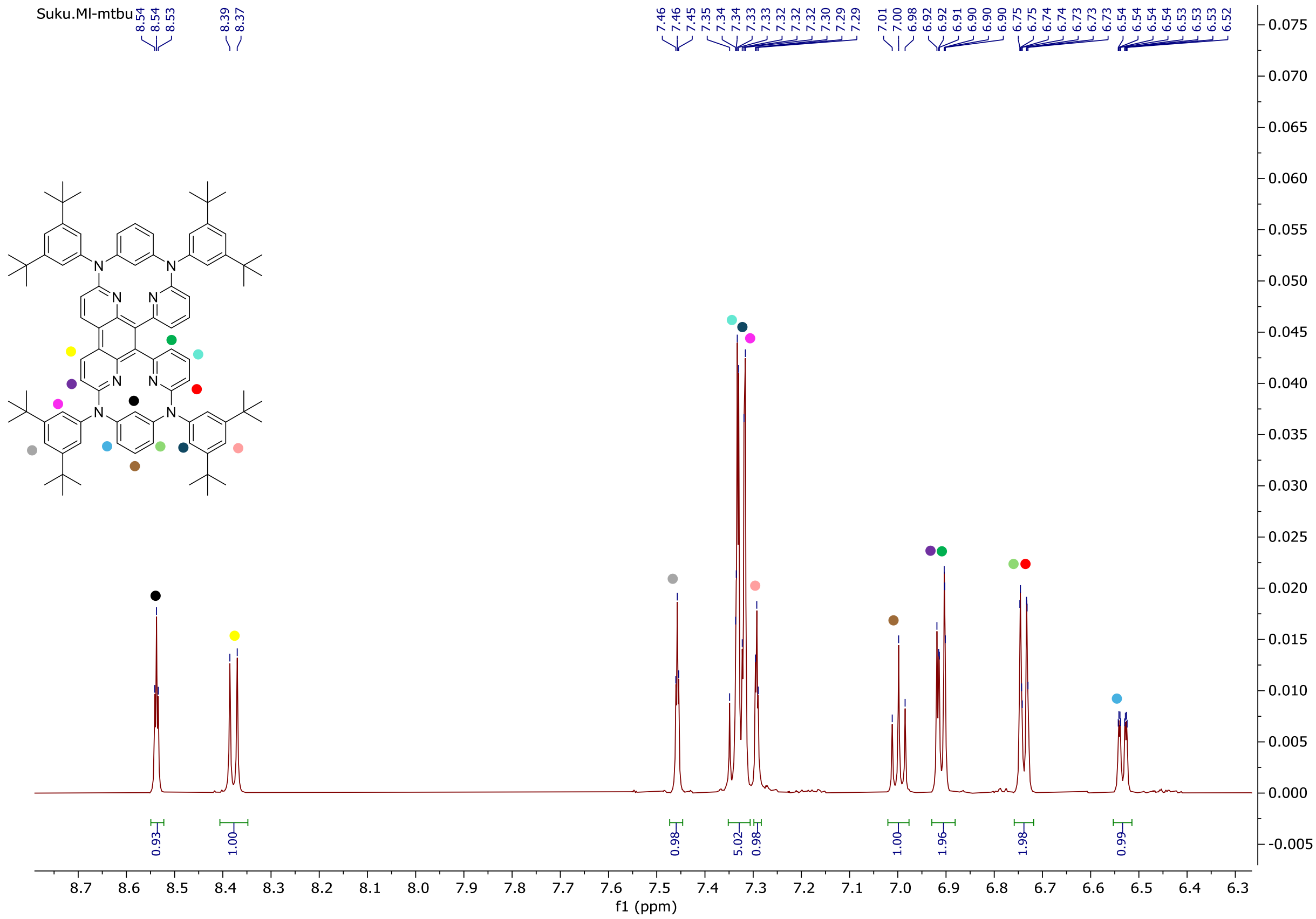

Figure S18. ^{1}H NMR spectrum (zoomed aromatic region) of **4** (CD_2Cl_2, 298K, 600 MHz).

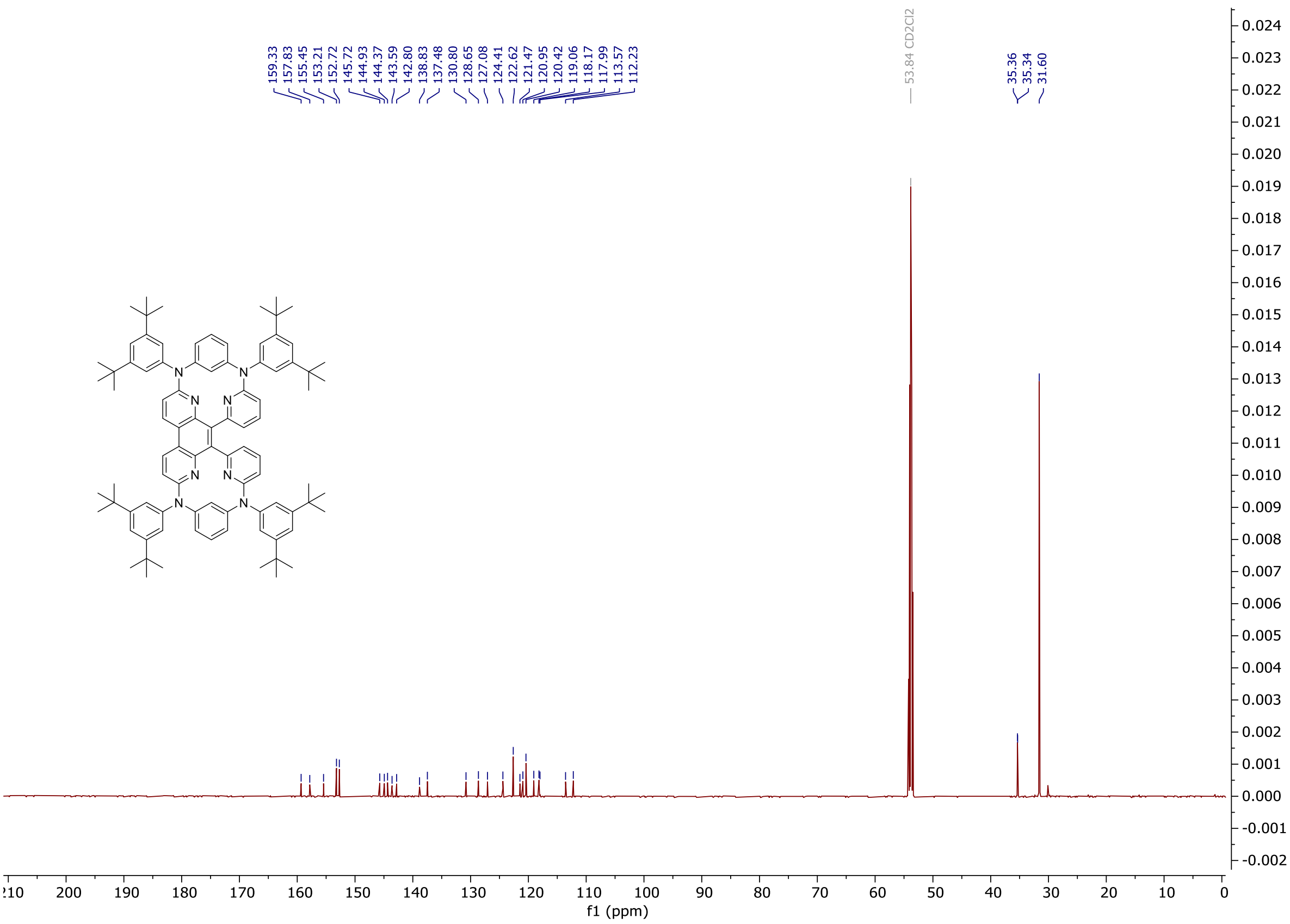

Figure S19. ^{13}C NMR spectrum of **4** (CD_2Cl_2, 298K, 151MHz).

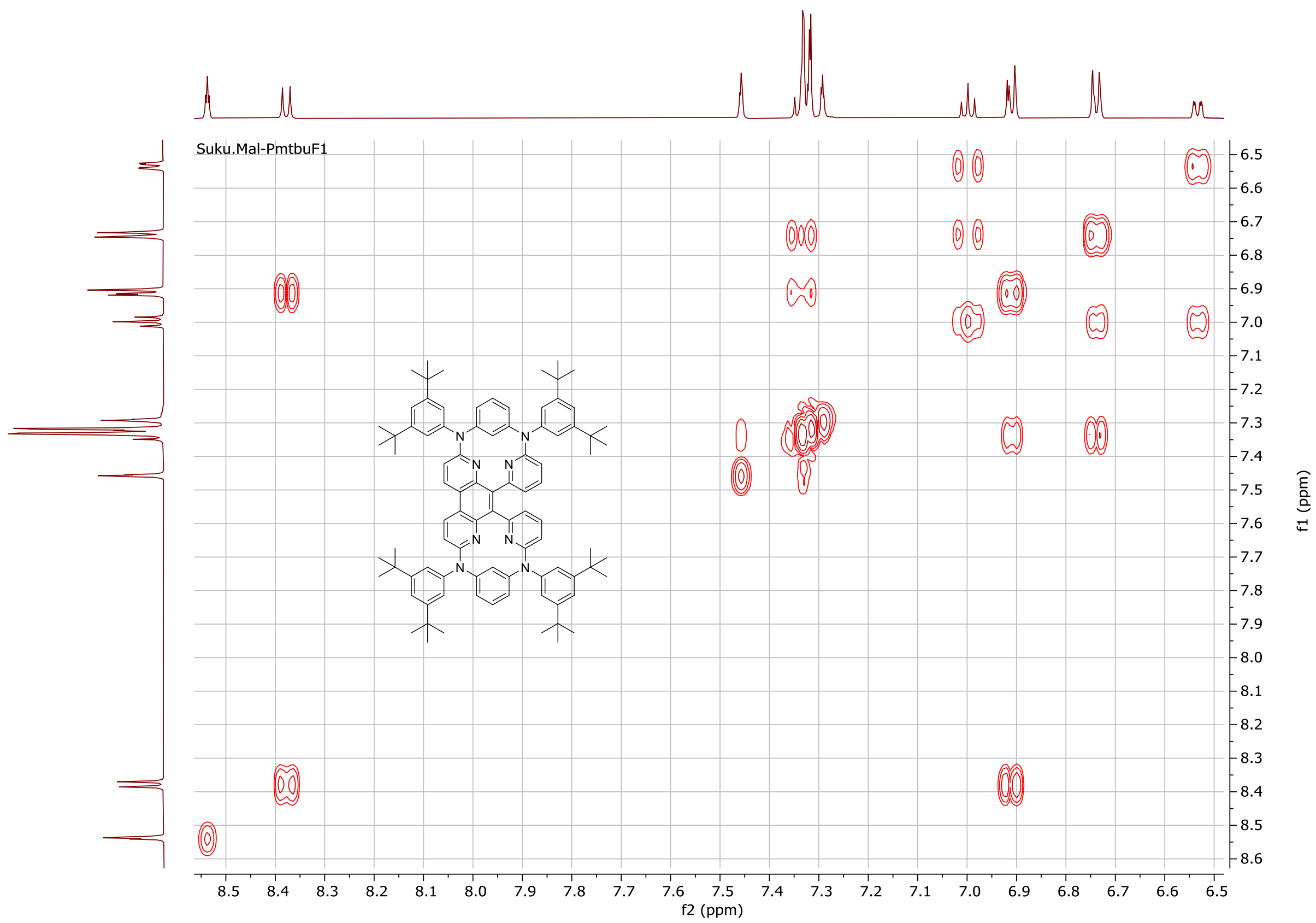

Figure S20. COSY NMR spectrum (zoomed aromatic region) of **4** (CD_2Cl_2, 298K, 400 MHz).

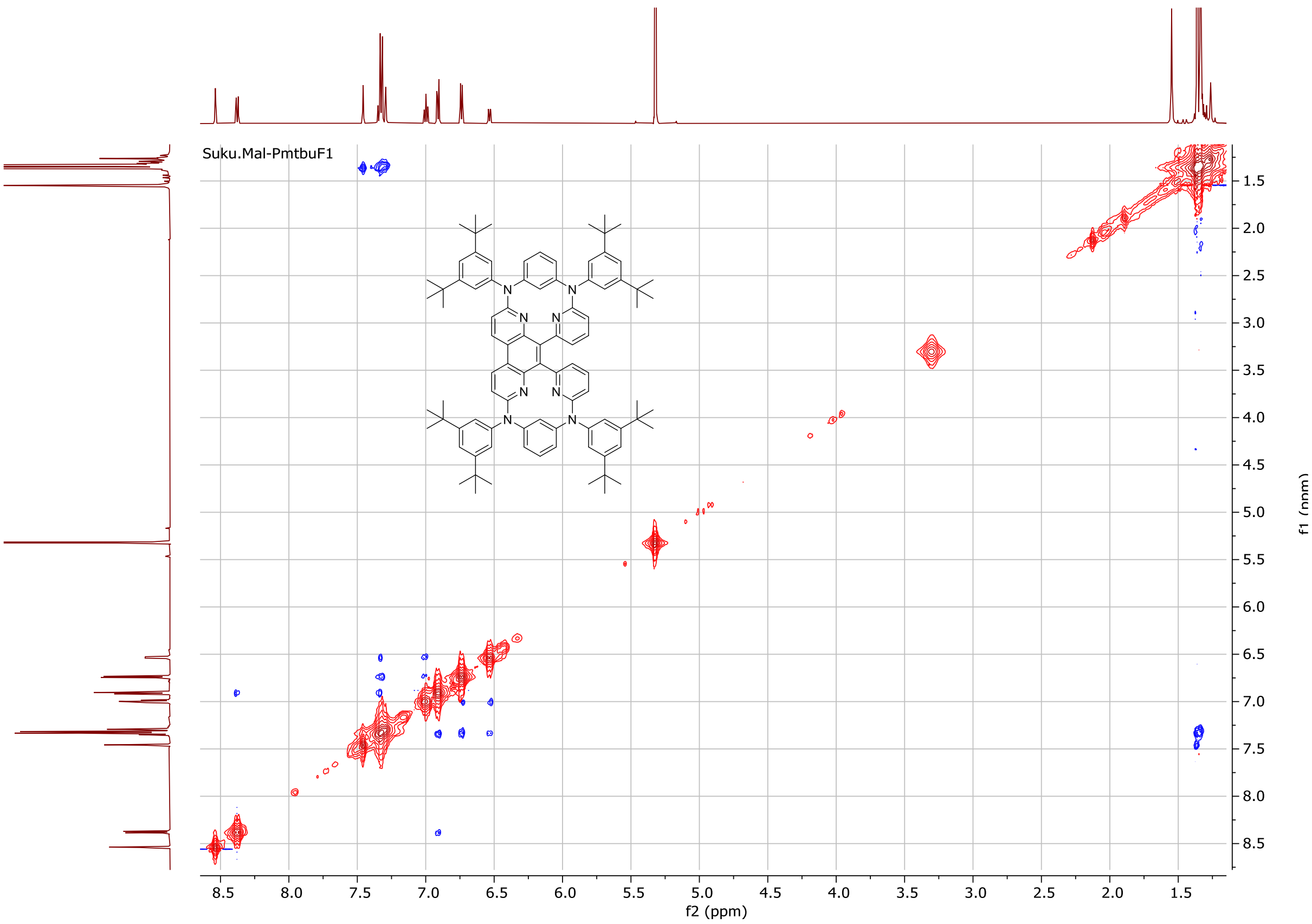

Figure S21. NOSEY NMR spectrum of **4** (CD_2Cl_2, 298K, 400 MHz).

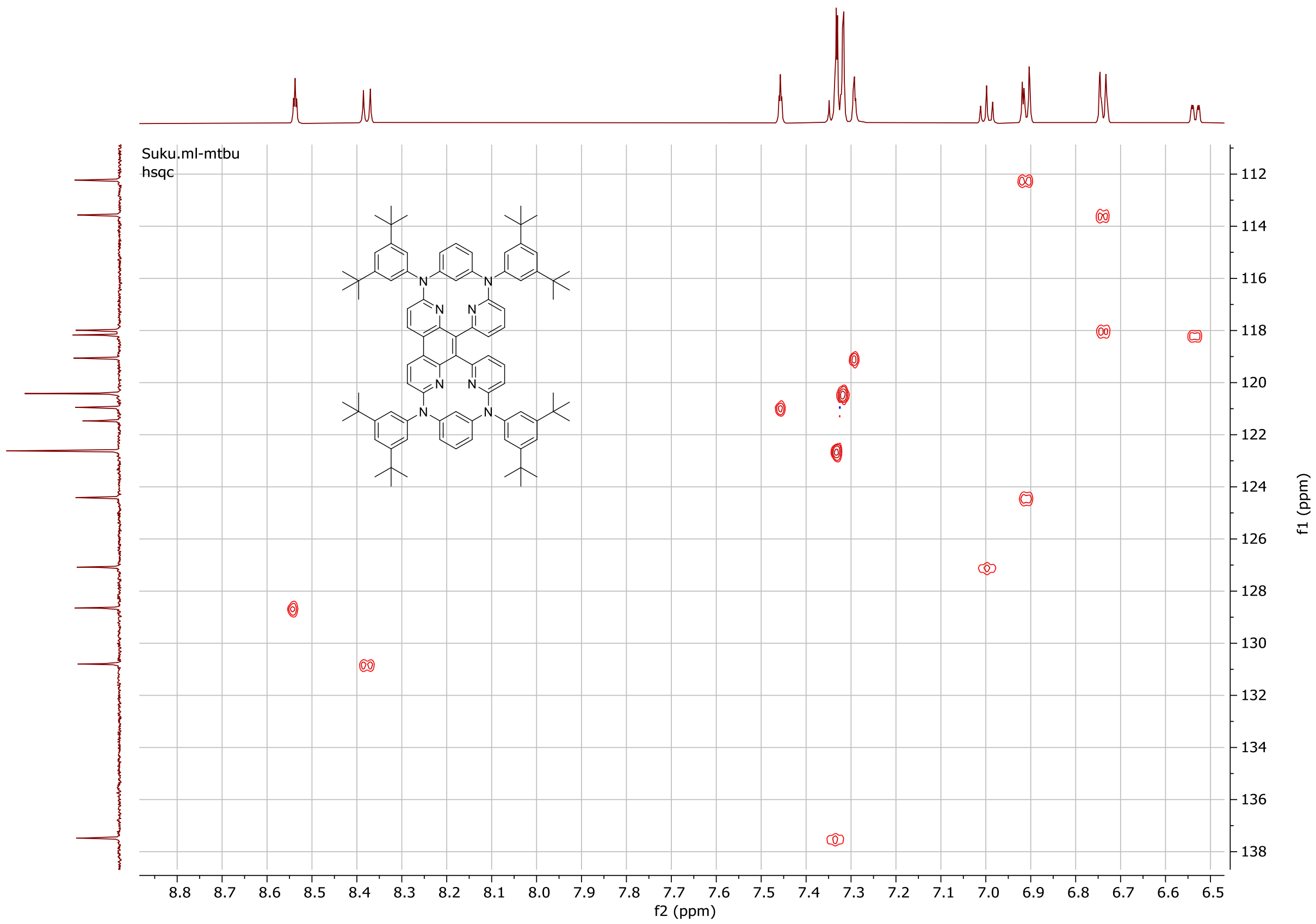

Figure S22. HSQC NMR spectrum (zoomed aromatic region) of **4** (CD_2Cl_2, 298K, 600, 151 MHz)

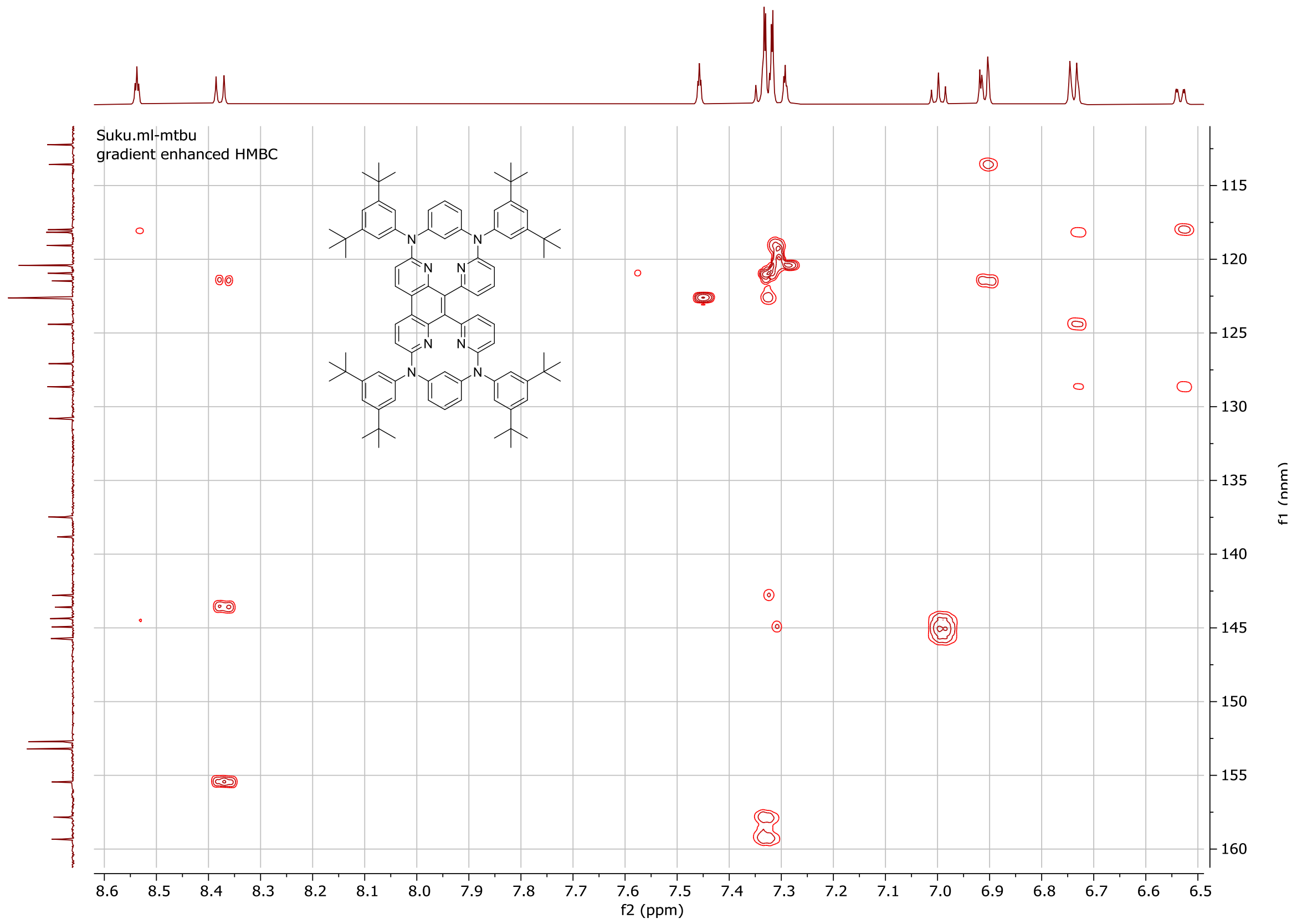

Figure S23. HMBC NMR spectrum (zoomed aromatic region) of **6** (CD_2Cl_2, 298K, 600, 151 MHz).

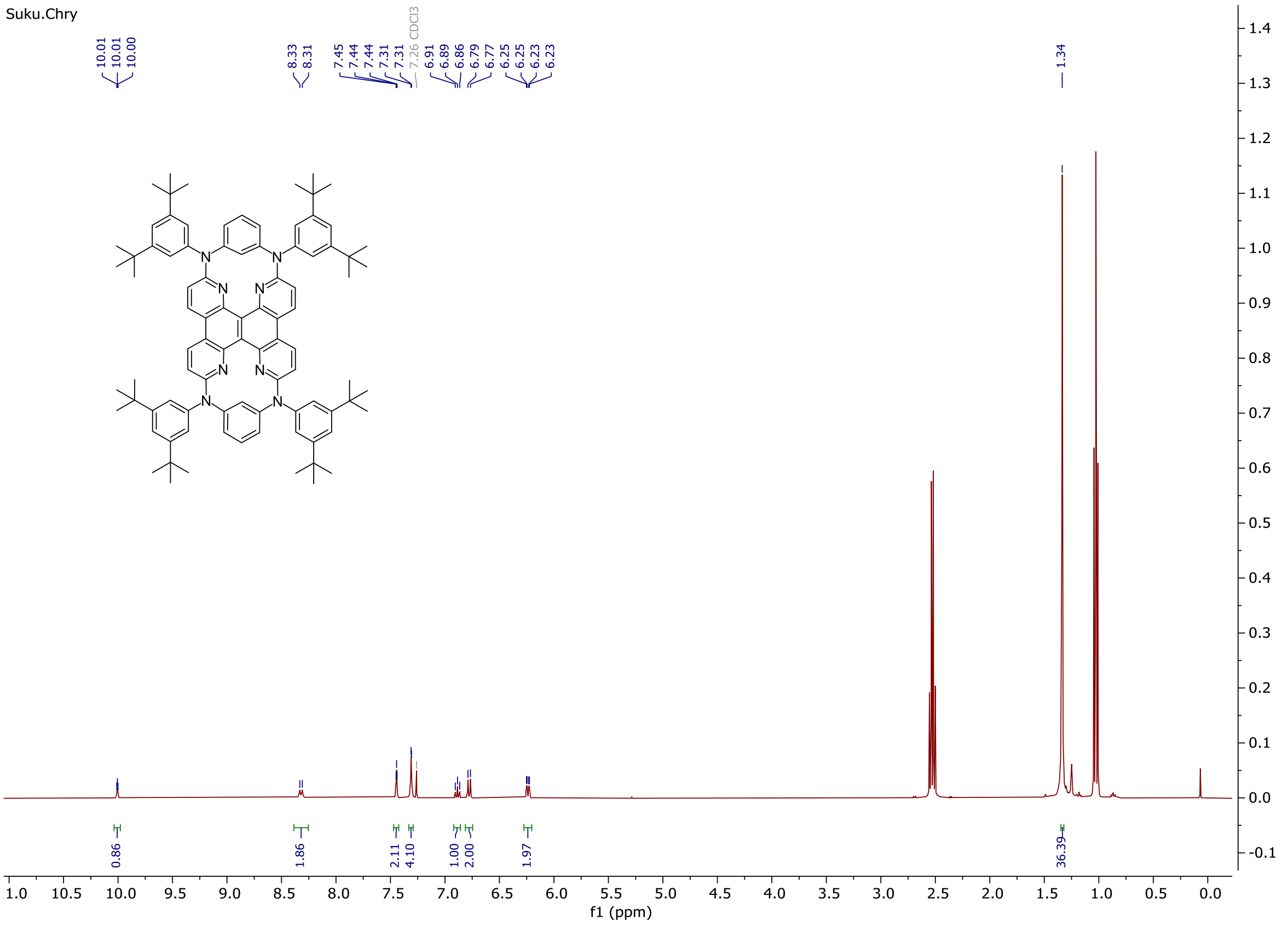

Figure S24. [1] H NMR spectrum of **1** (10ul Et_3N in 0.6ml of $CDCl_3$) 298K, 400 MHz).

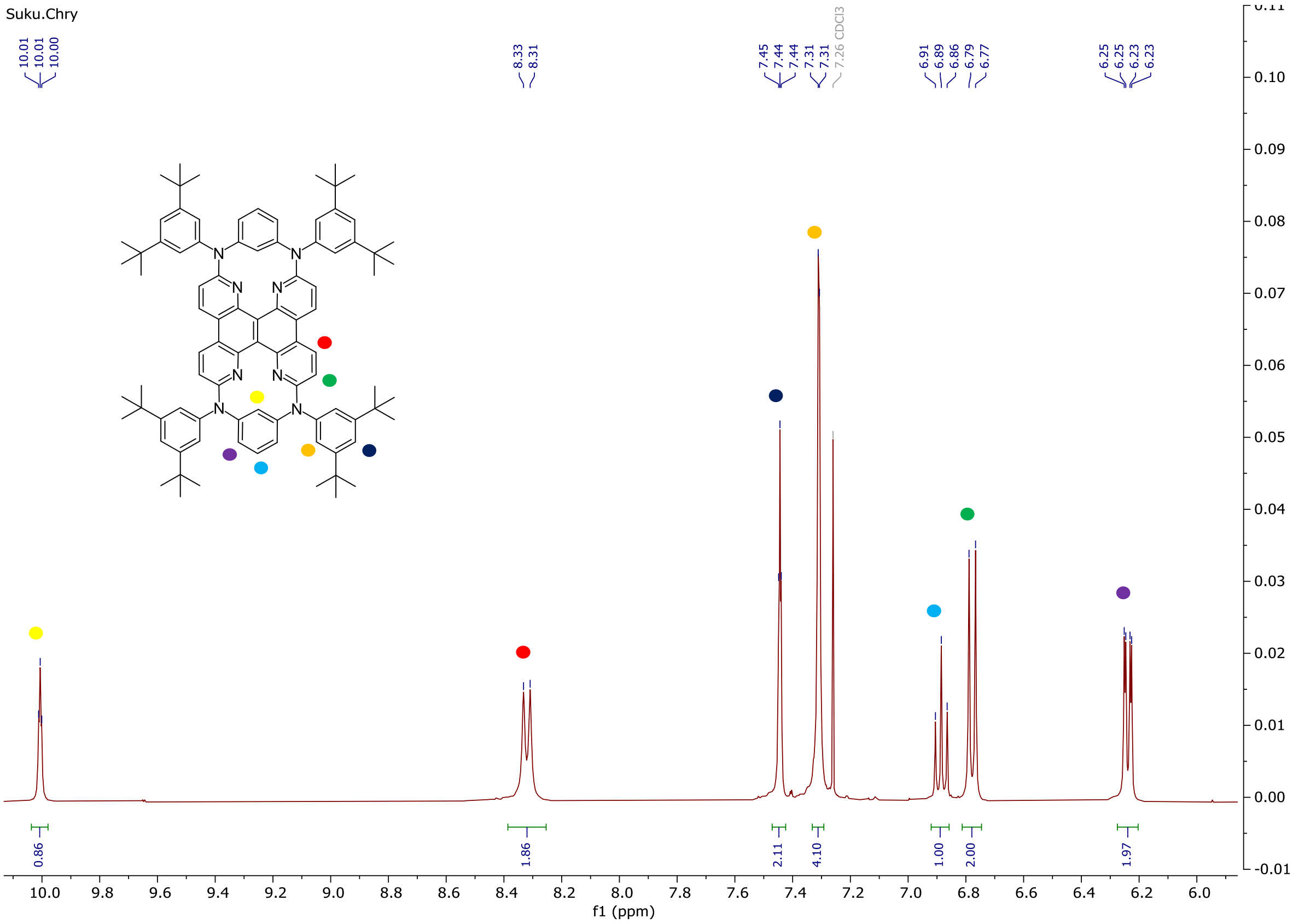

Figure S25. ^{1}H NMR spectrum (zoomed aromatic region) of **1** (CD_2Cl_2, 298K, 400 MHz).

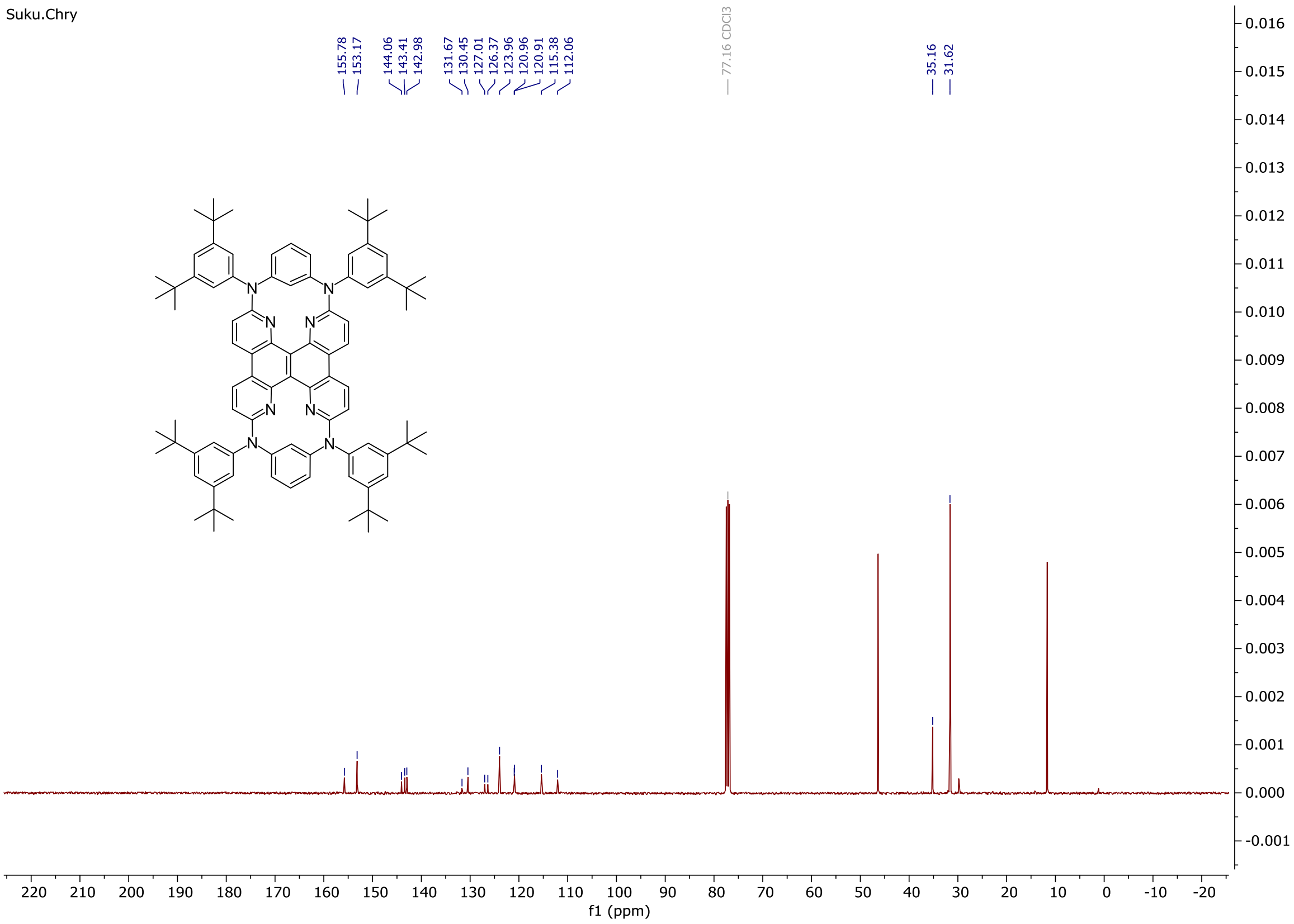

Figure S26. ^{13}C NMR spectrum of **1** 10ul Et_3N in 0.6ml of $CDCl_3$) 298K, 101MHz).

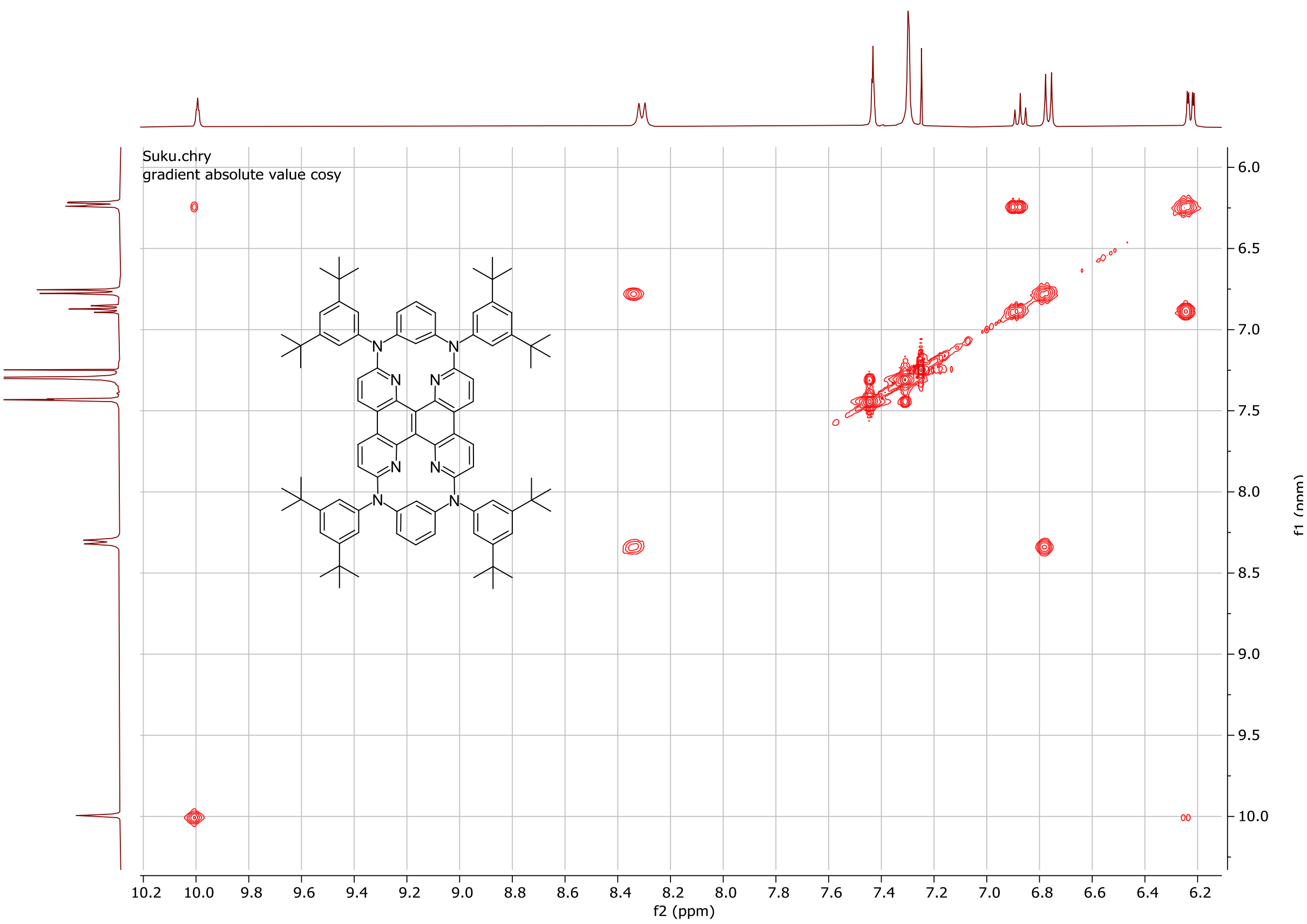

Figure S27. COSY NMR spectrum of **1** (10ul Et_3N in 0.6ml of $CDCl_3$) 298K, 400MHz).

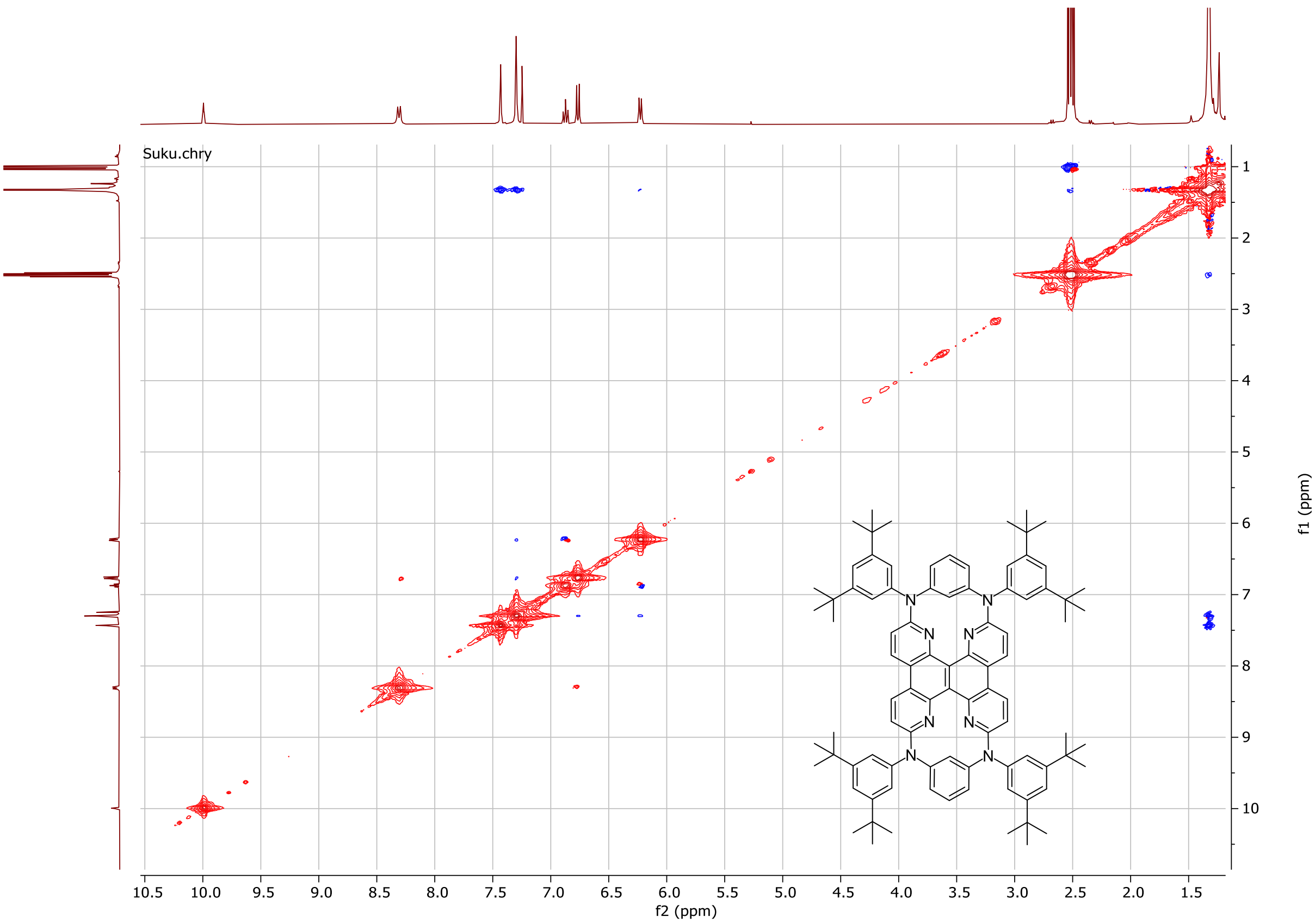

Figure S28. NOESY NMR spectrum of **1** (10ul Et_3N in 0.6ml of $CDCl_3$) 298K, 400MHz).

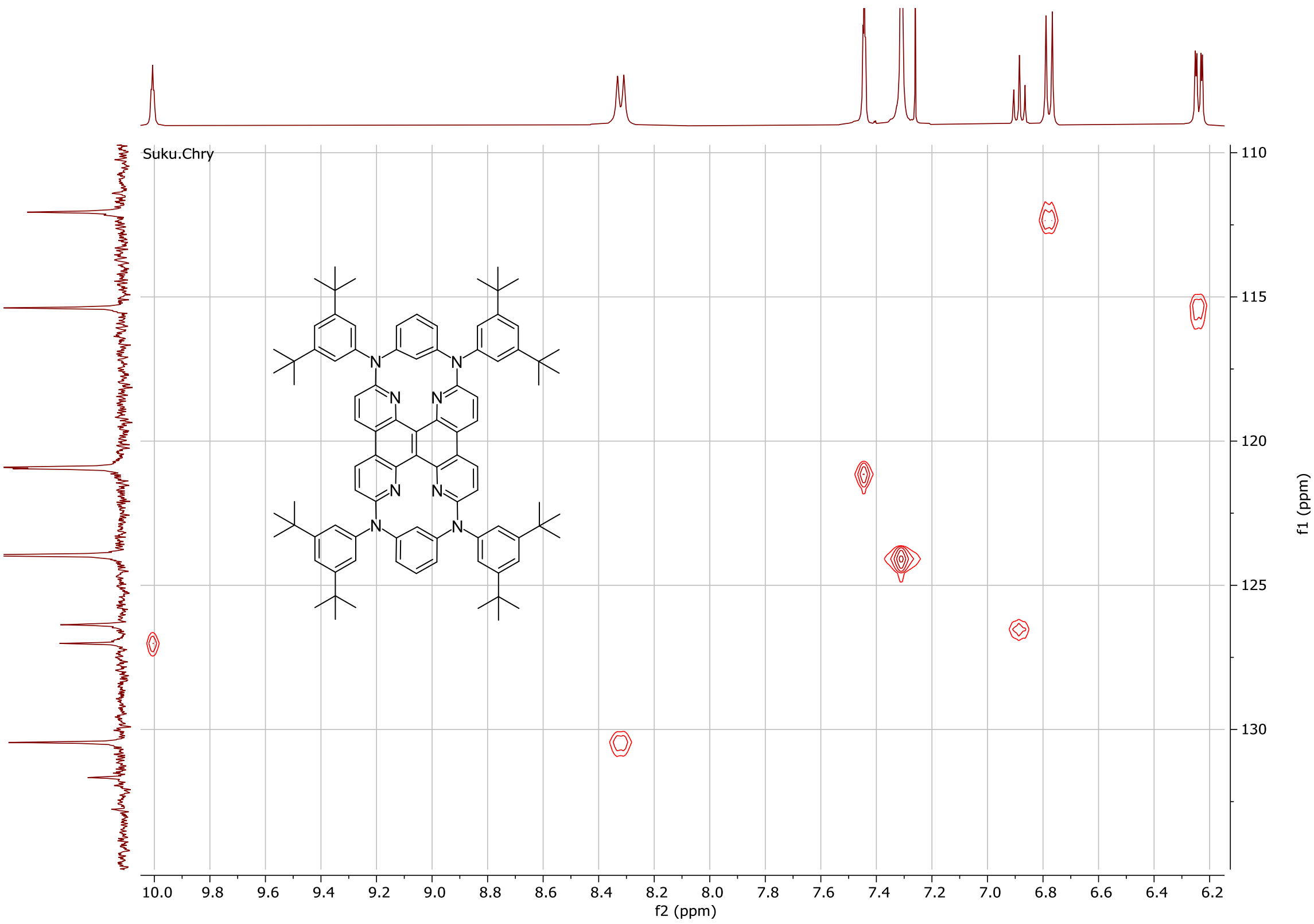

Figure S29. HSQC NMR spectrum (zoomed aromatic region) of **1** (10ul Et_3N in 0.6ml of $CDCl_3$, 298K, 400, 101 MHz).

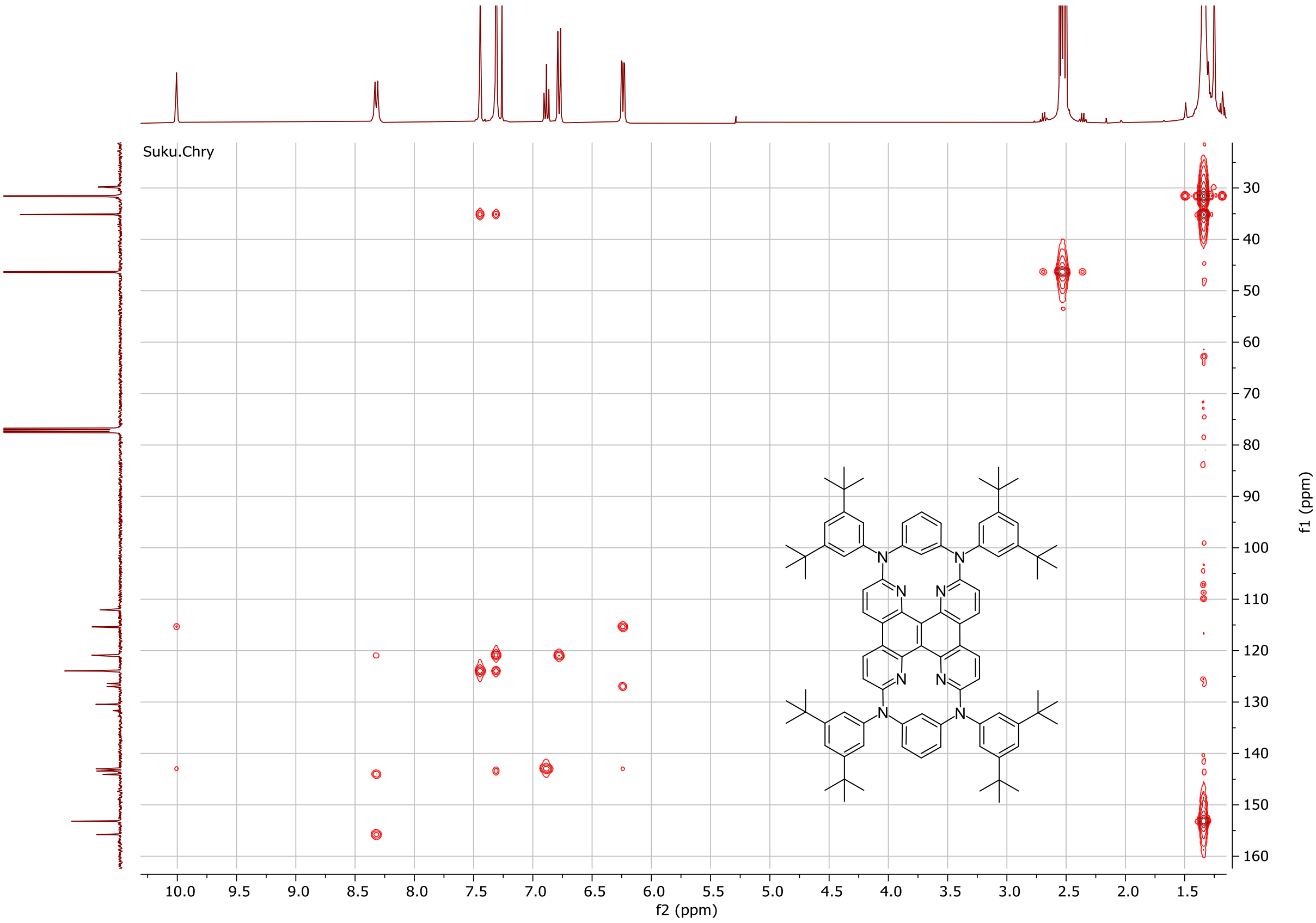

Figure S30. HMBC NMR spectrum of **1** (10ul Et_3N in 0.6ml of $CDCl_3$, 298K, 400, 101 MHz).

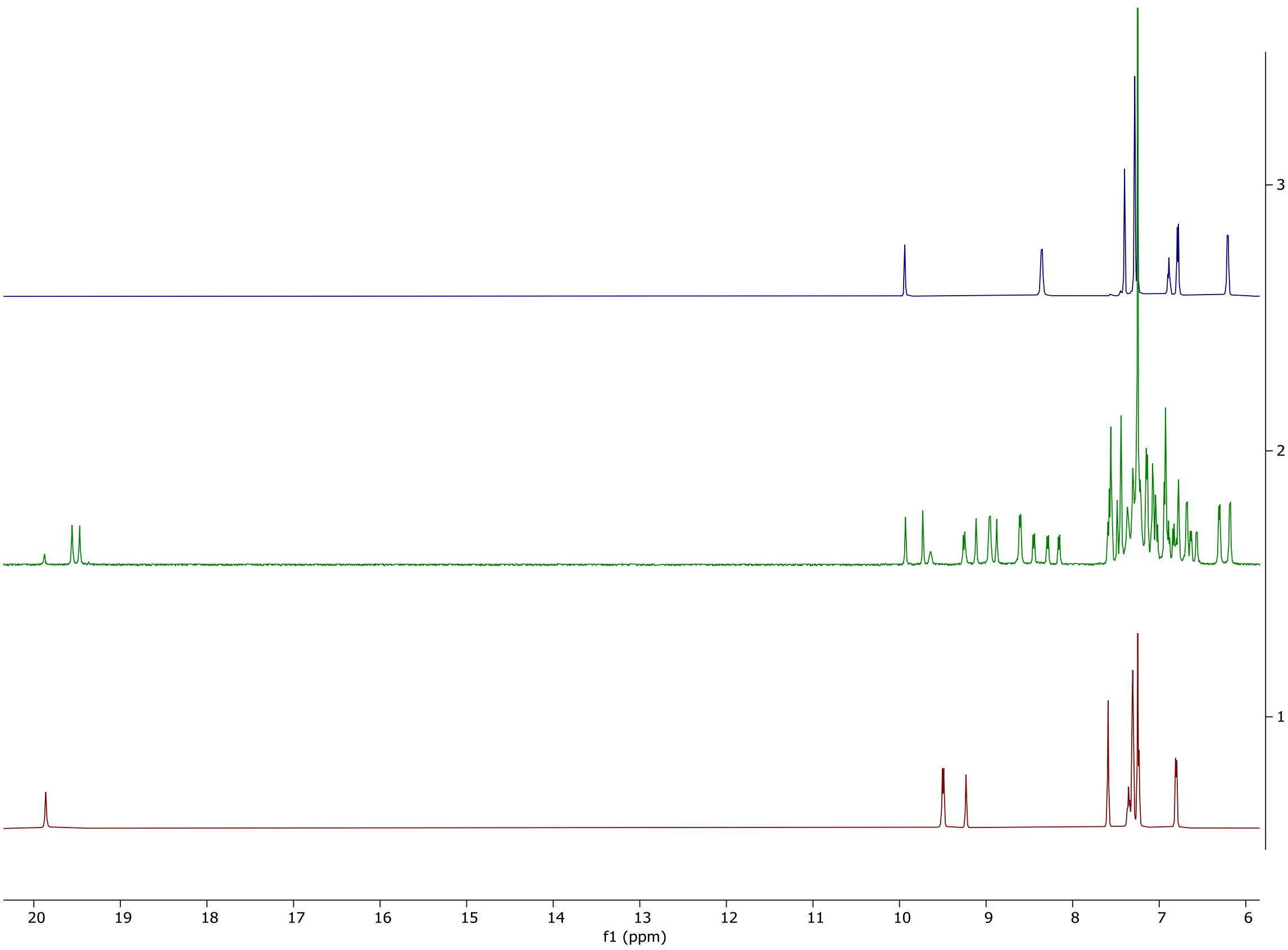

Figure S31. Zoom on aromatic region of the spectra during ^{1}H NMR-monitored titration of **1** with TFA ($CDCl_3$, 600 MHz, 233 K). 1 – 2 eq TFA, 2 -1 eq TFA, 3 – 0 eq TFA.

4. UV-VIS spectra

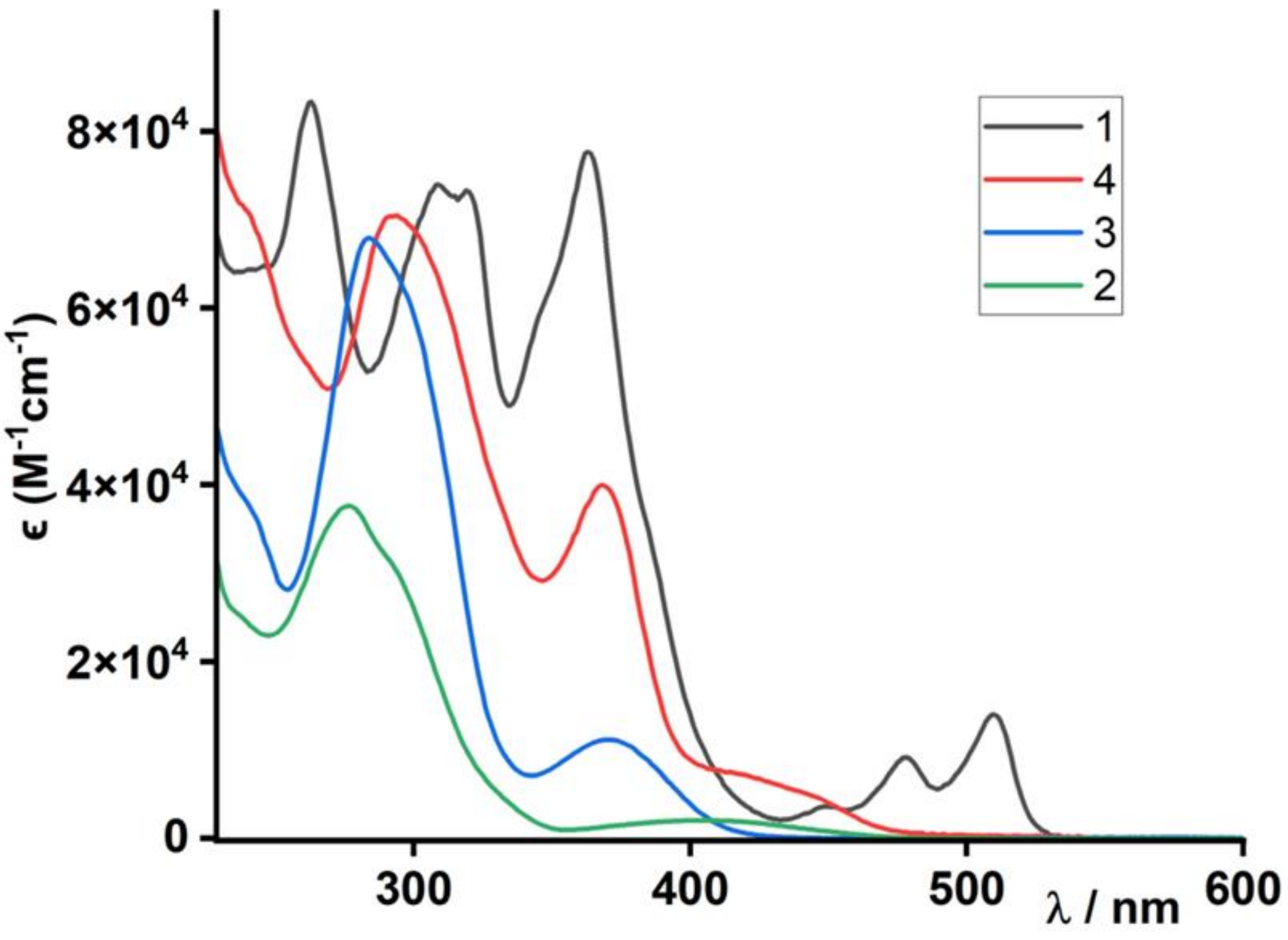

Figure S32. Molar Extinction spectra of the neutral macrocycles **4, 3, 2 and 1** in cyclohexane at 298 K.

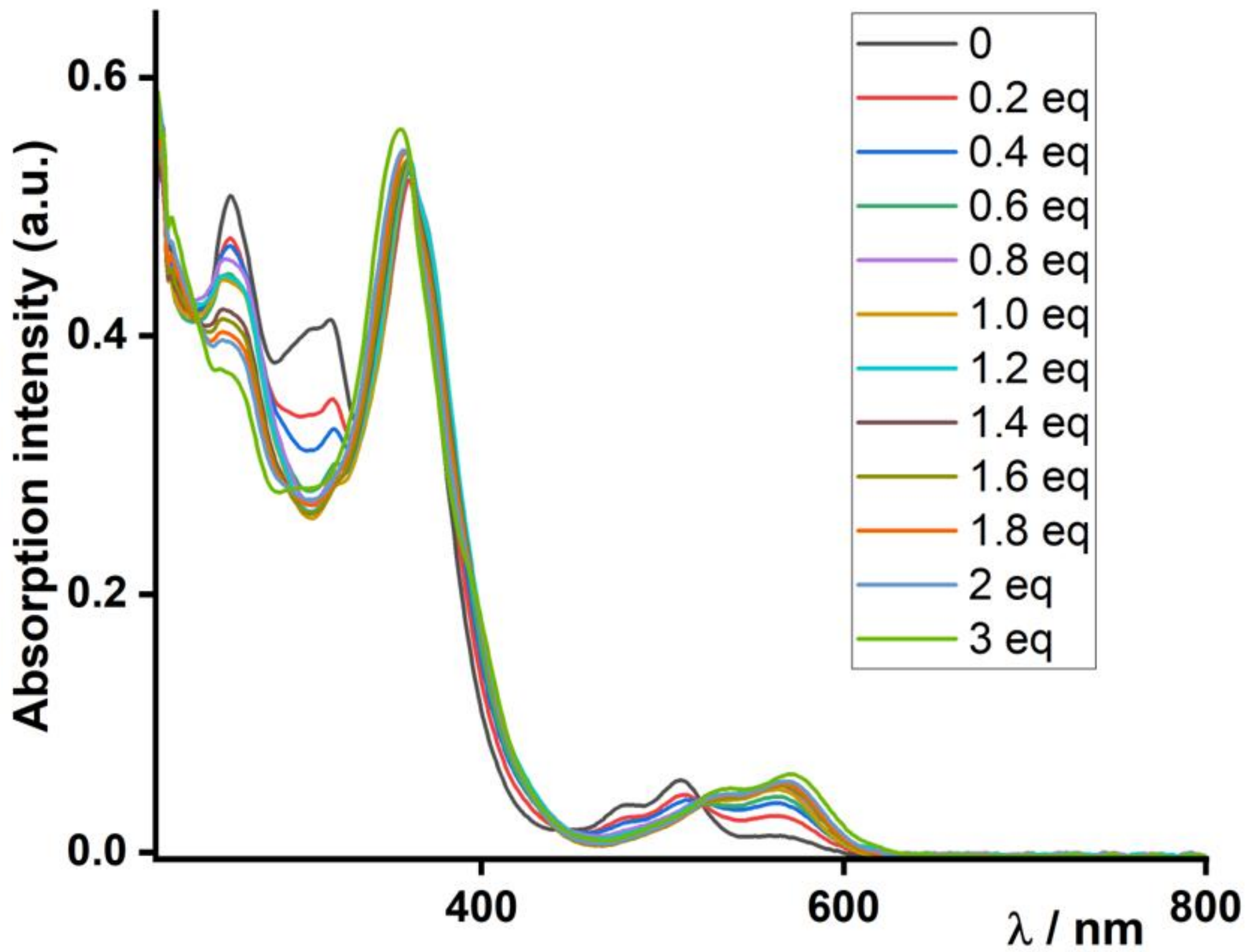

Figure S33. Absorption spectra recorded during titration of macrocycle **1** with CF_3COOH in DCM at 298K.

5. Emission spectra

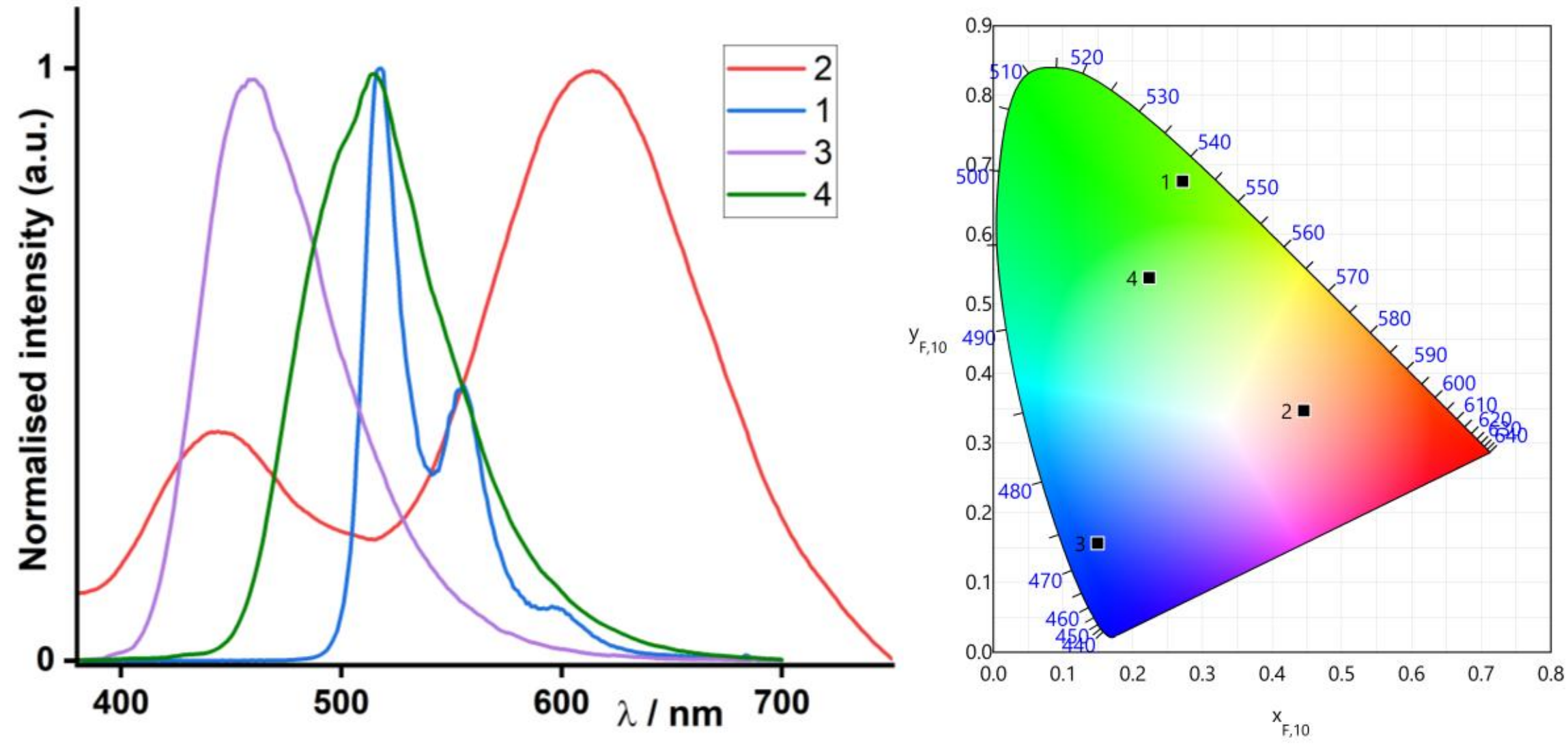

Figure S34. Normalized fluorescence emission spectra (left) and CIE 2015 colour coordinates of fluorescence emission spectra of the macrocycles **2, 3, 4 and 1** in cyclohexane at 298 K.

Structures	$\lambda_{emission}$[a], nm	$\lambda_{exitation}$[b], nm	Φ[c] (FLQY)	τ (ns)
2	614	310	1%	<1
3	459	358	8%	4.7
4	514	370	16%	5.4
1	517	347	43%	8.8

[a] Emission maxima. [b] Excitation wavelength. [c] Fluorescence Quantum yield.

Table S1. Fluorescence emission properties for macrocycles **2, 3, 4** and **1** in cyclohexane.

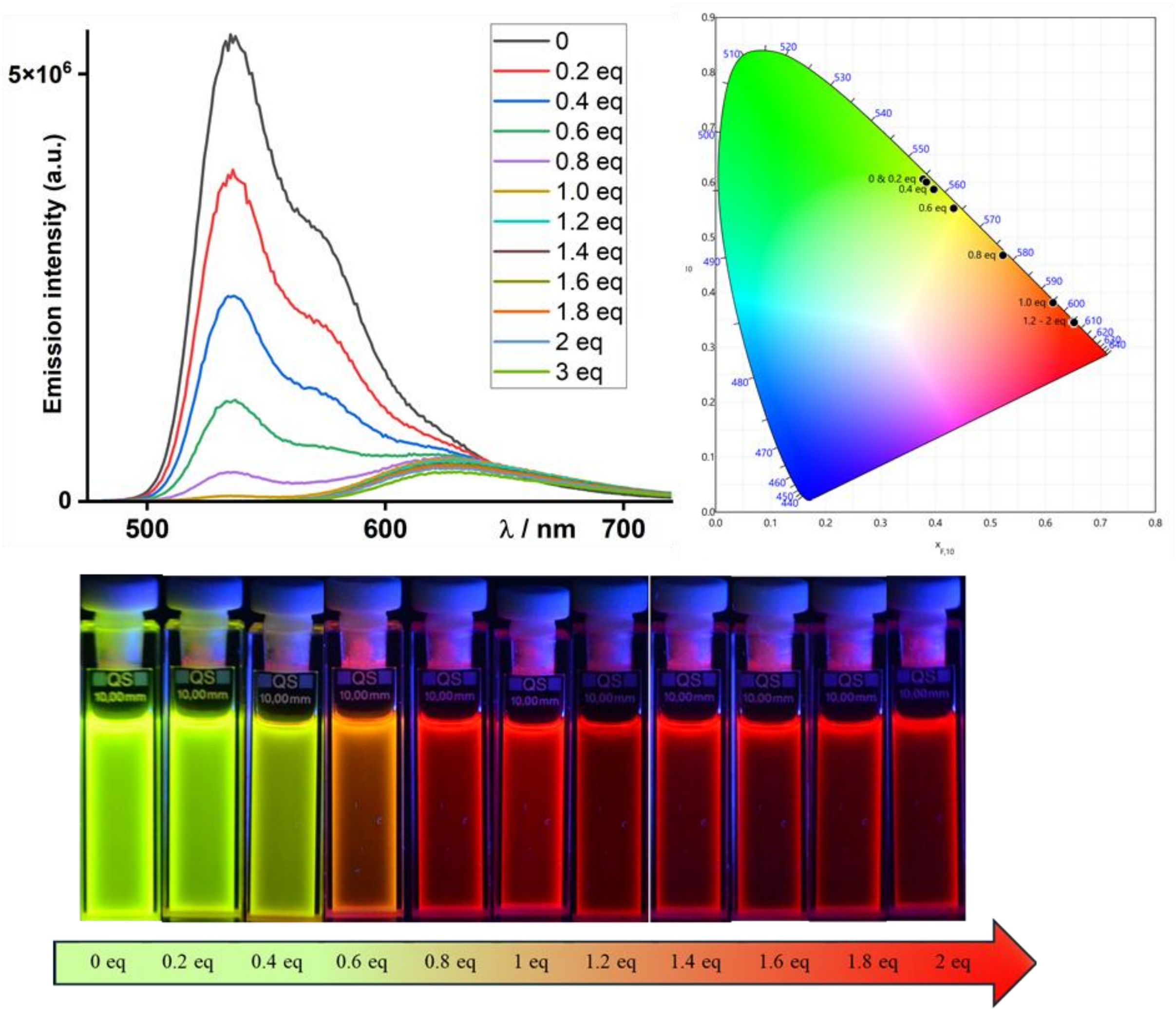

Figure S35. Fluorescence Emission spectra (top left), CIE 2015 colour coordinates (top right) and corresponding photographs (bottom) recorded during titration of macrocycle **1** with CF_3COOH in DCM at 298K.

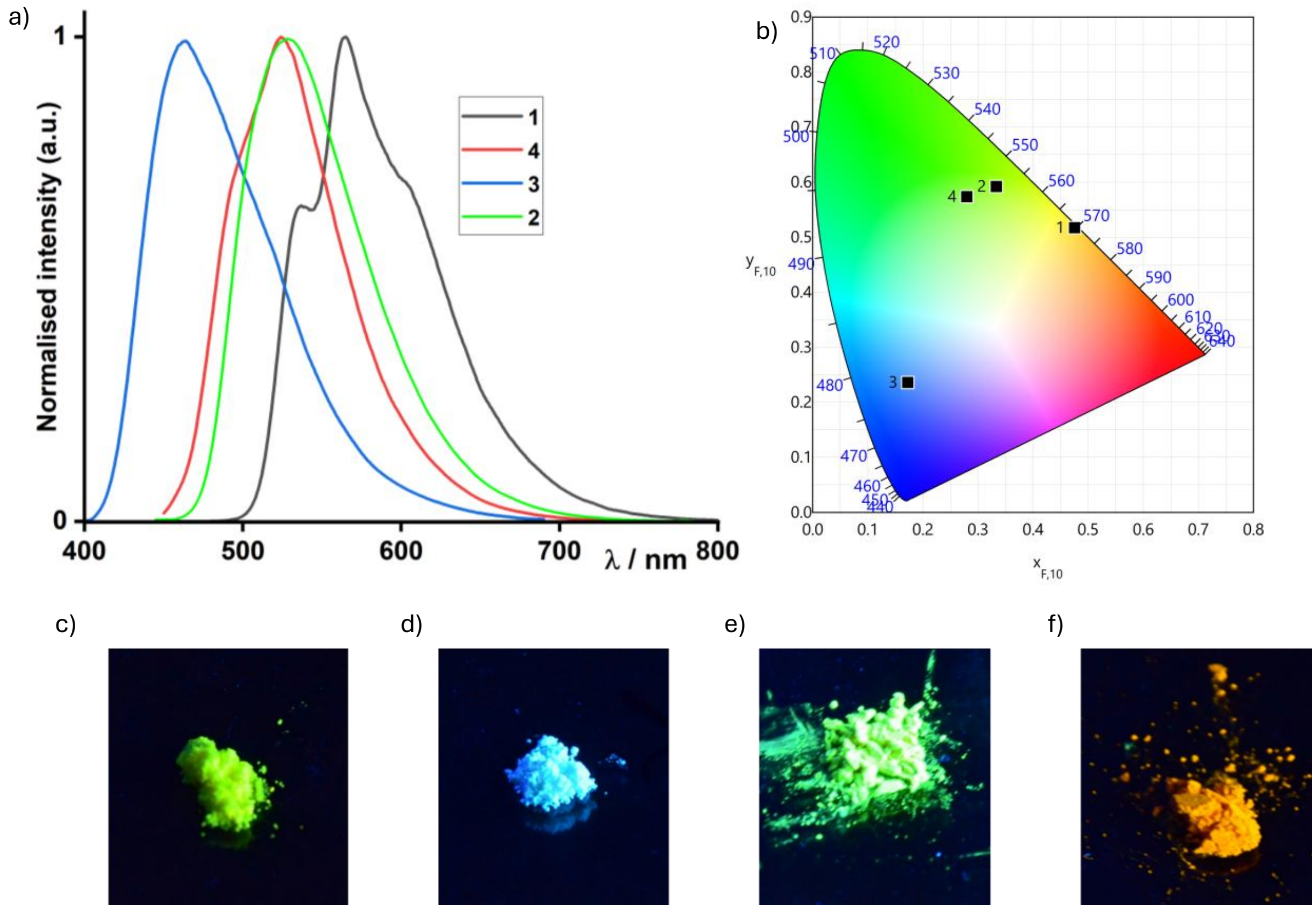

Figure S36. a) Normalized solid state emission spectra in solid state, b) CIE 2015 colour coordinates of solid-state emission, c, d, e, f) pictures of solid-state emission of the macrocycles **2**, **3**, **4** and **1** respectively at 298 K.

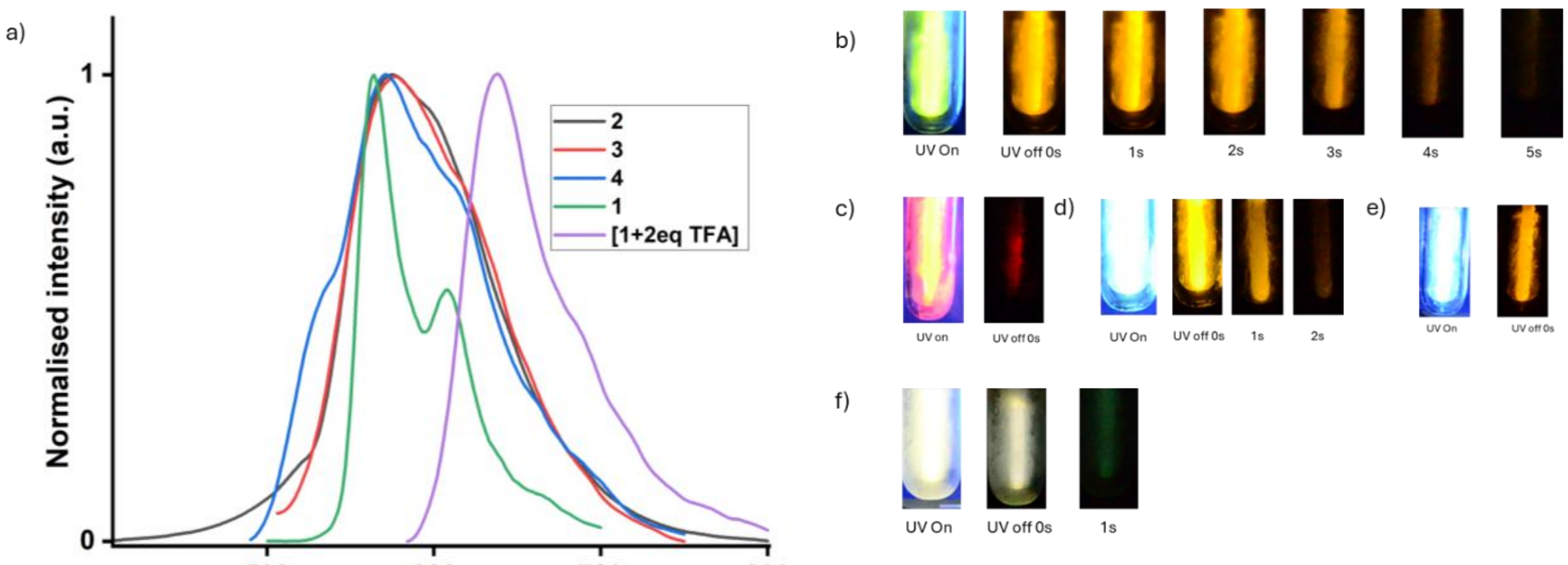

Figure S37. a) Phosphorescence emission spectra, b, c, d, e and f pictures of Phosphorescence emission of macrocycles **1,** [**1+2eq TFA]**, **4, 3** and **2** respectively in MeTHF at 77K.

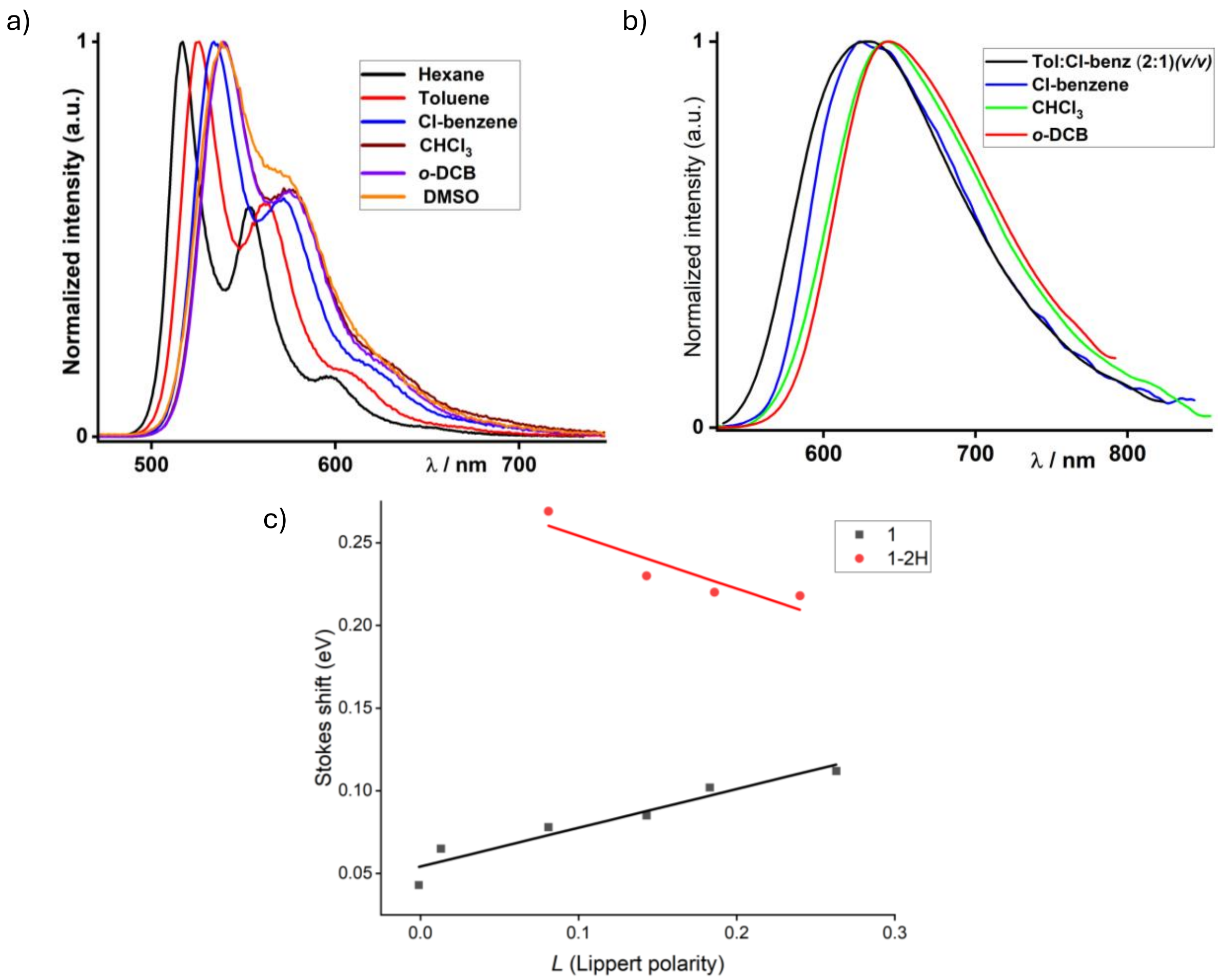

Figure S38. Solvatochromism of a) **1** and b) **1-2H** in various solvents, c) the Lippert Mataga Plot.

6. Lifetime Measurement

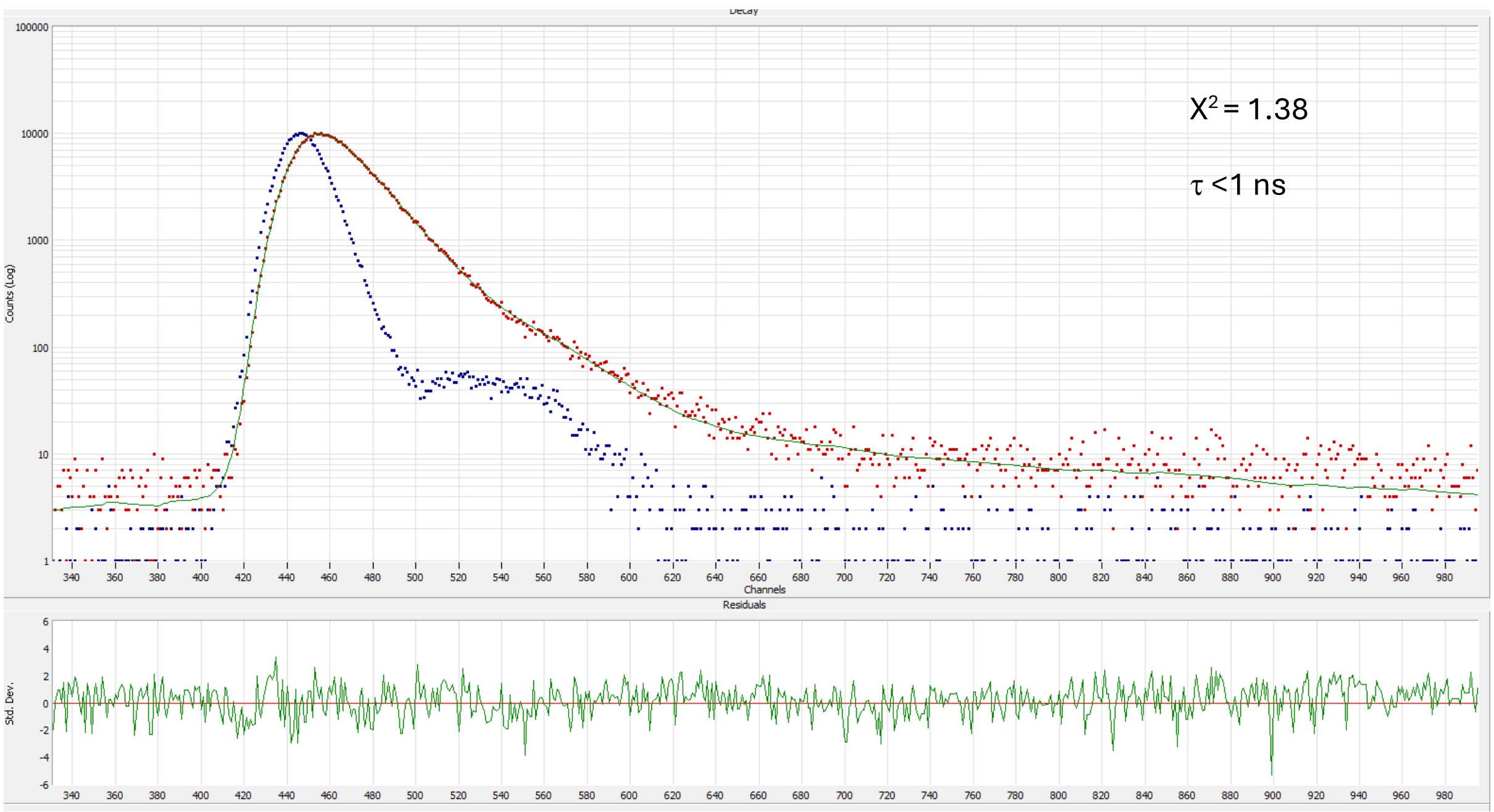

Figure S39. Fluorescence decay profile of **2** in cyclohexane at 298 K with a lifetime of <1 ns. (Calculated using triple-exponential, red dots - compound, blue dots - IRF (excitation))

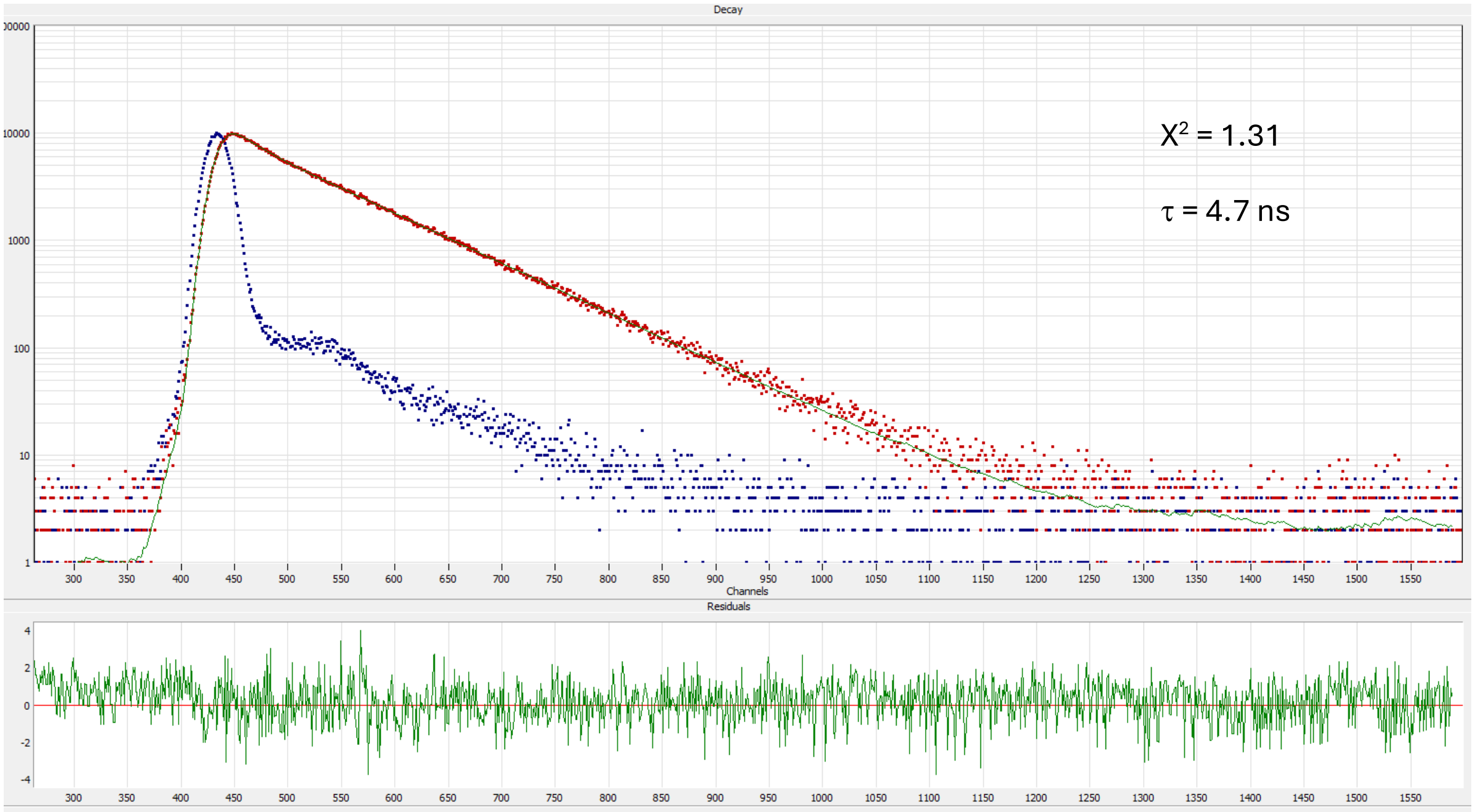

Figure S40. Fluorescence decay profile of **3** in cyclohexane at 298 K with a lifetime of 4.7 ns. (Calculated using mono-exponential, red dots -compound, blue dots - IRF (excitation))

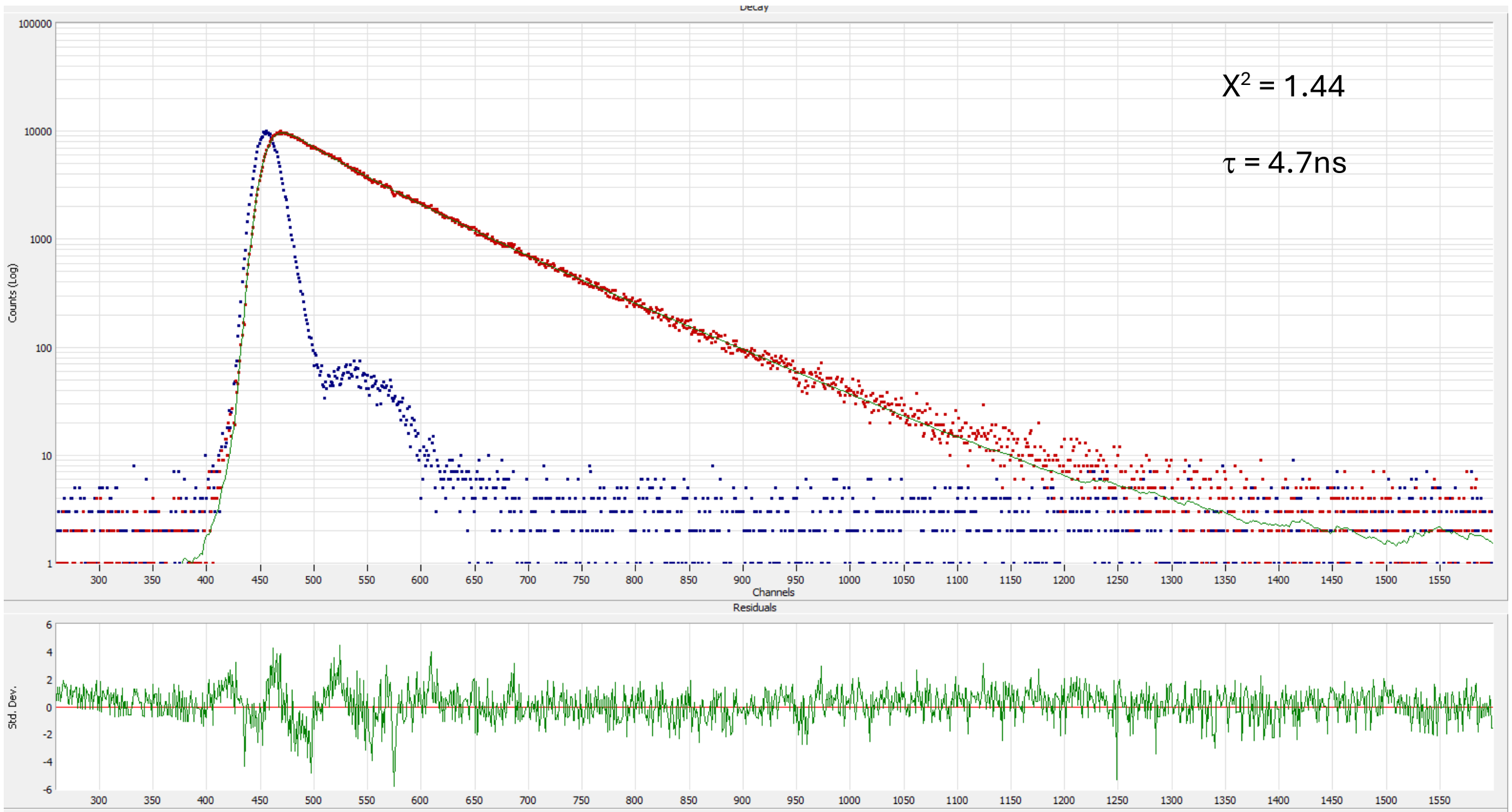

Figure S41. Fluorescence decay profile of **4** in cyclohexane at 298 K with a lifetime of 5.4 ns. (Calculated using double-exponential, red dots -compound, blue dots - IRF (excitation))

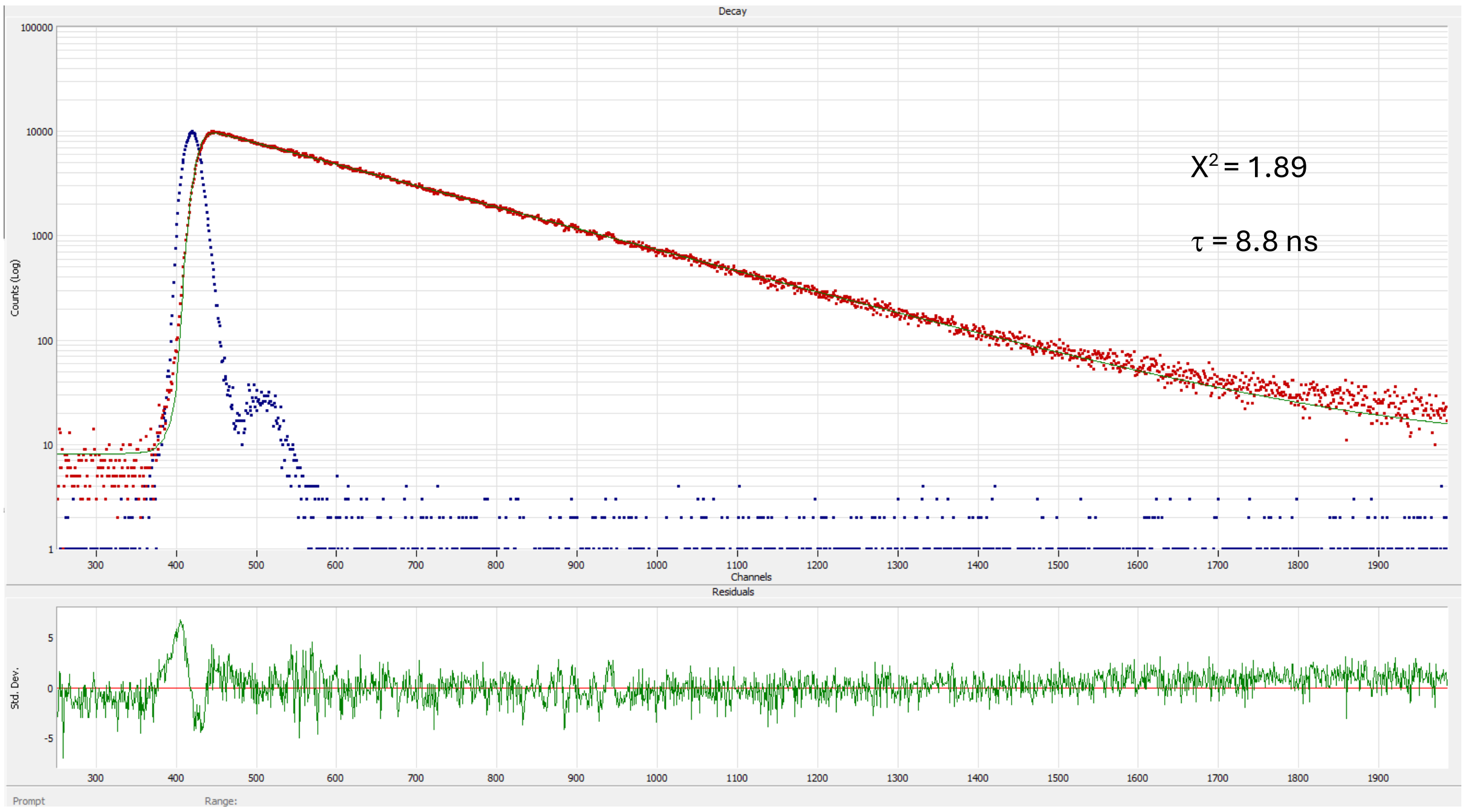

Figure S42. Fluorescence decay profile of **1** in cyclohexane at 298 K with a lifetime of 8.8 ns. (Calculated using mono-exponential, red dots -compound, blue dots - IRF (excitation))

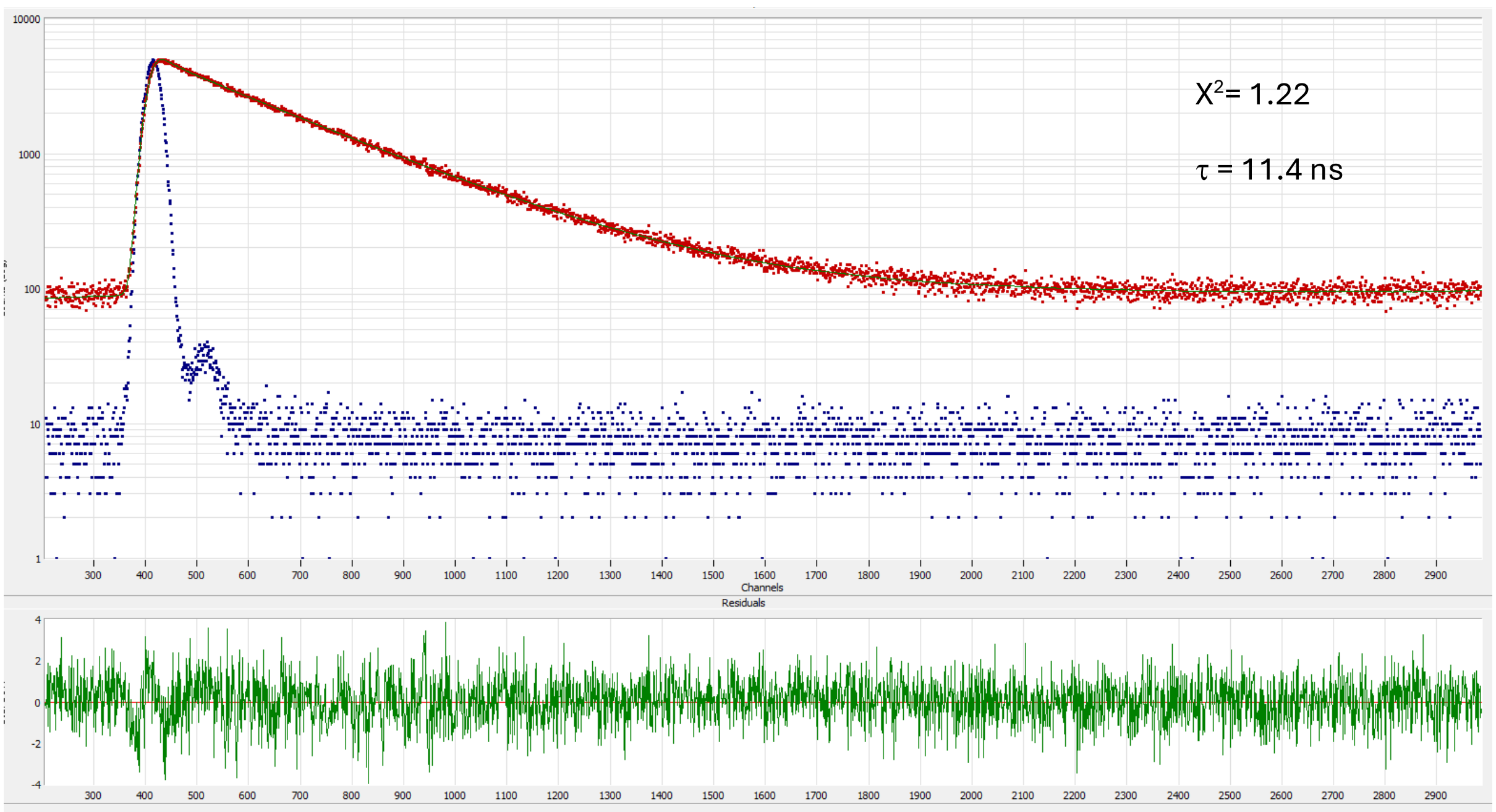

Figure S43. Fluorescence decay profile of [**1+2eq TFA]** in cyclohexane at 298 K with a lifetime of 11.4 ns. (Calculated using double-exponential, red dots -compound, blue dots - IRF (excitation))

7. TADF experiments.

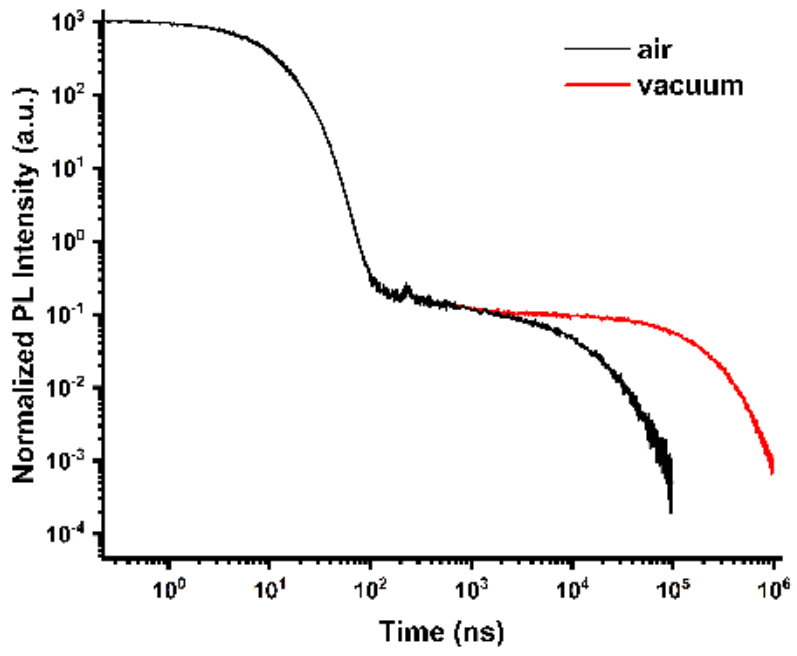

1	PLQY [%]	τ_{PF} [ns]	τ_{DF} [μs]
Air	39	9.8	14.4
Vacuum	93	9.8	190

Figure S44. PL intensity decays of **1** dispersed in ZNX film 0.1 % (w/w) under air conditions (normal pressure) and vacuum.

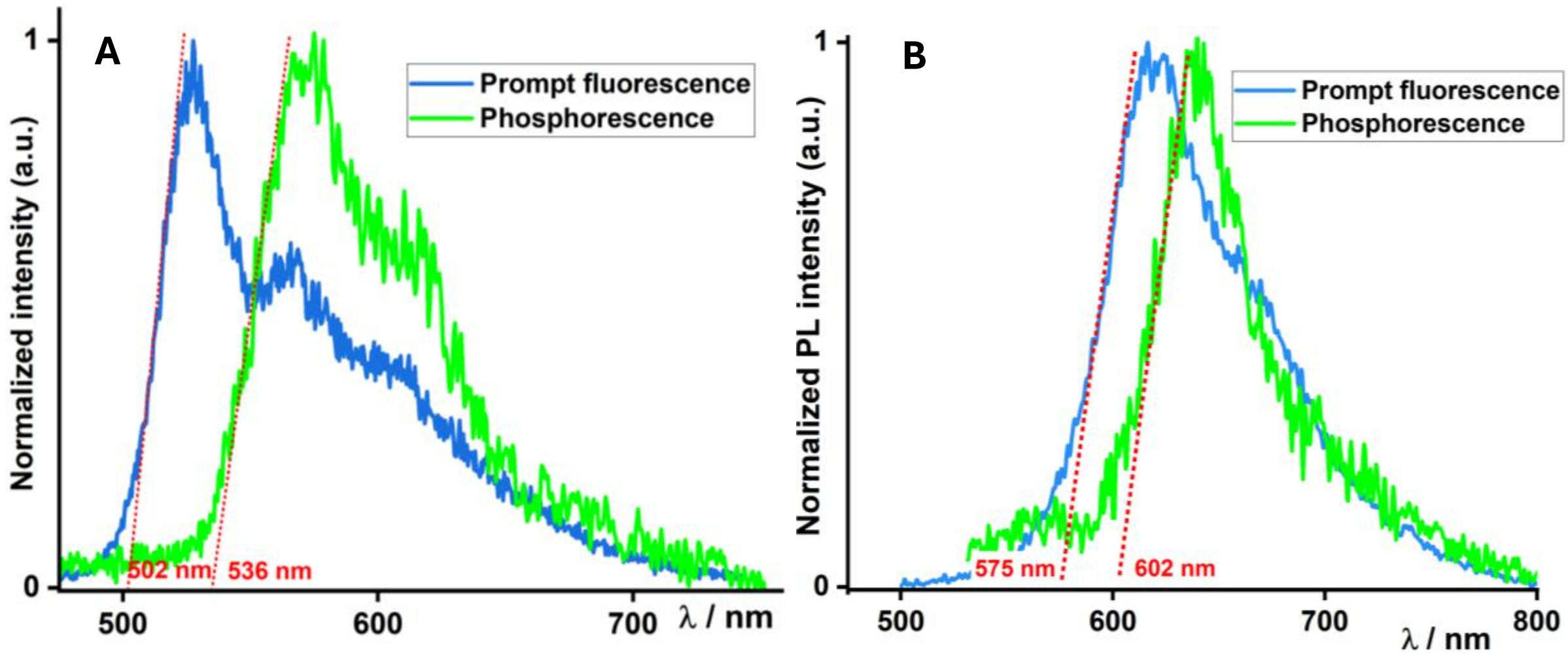

Sample	$\lambda_{flu\ onset}$ (nm)	$\lambda_{phos\ onset}$ (nm)	ΔE_{ST} (eV)
**1**/ZNX	527 (onset-502)	573 (onset-536)	0.16
**1-2H**/ZNX	620 (onset-575)	639 (onset-602)	0.11

Figure S45. Time-resolved emission spectra of **1** (A) and **1-2H** (B) in ZNX films at 10 K. Prompt fluorescence (PF) and phosphorescence spectra were recorded within 1-50 ns and 1-10 ms time delay, respectively.

Determination of photophysical parameters. Photophysical rate constants k_r, k_{ISC}, and k_{rISC} were calculated according to equations described previously[13] and below / in SI.
PL decay curves were fitted with the multiexponential equation:

$$I(t) = A_0 + \sum_{i=1}^{n} A_i \exp\left(-{}^{t}/_{\tau_i}\right) \tag{S1}$$

where A_i is the pre-exponential factor, τ_i is the decay time and $I(t)$ is emission intensity. R^2, χ^2 - were evaluated for the complete decay using the multiexponential function. Average lifetimes of prompt (τ_{PF}) and delayed fluorescence (τ_{DF}) were determined using the following formula:

$$\tau_{PF}, \tau_{DF} = \sum_{i=1}^{n} f_i \tau_i, \tag{S2}$$

where f_i is fractional contribution of i-th component expressed as:

$$f_i = \frac{A_i \tau_i}{\sum_{i=1}^{n} A_i \tau_i} \tag{S3}$$

The ratio of DF and PF quantum yields $\varphi_{DF}/\varphi_{PF}$ was determined as follows:

$$\frac{\varphi_{DF}}{\varphi_{PF}} = \frac{\sum_{i=1}^{n} \tau_{DF(i)} A_{DF(i)}}{\sum_{j=1}^{n} \tau_{PF(j)} A_{PF(j)}} \tag{S4}$$

where $A_{DF(i)}$ and $A_{PF(j)}$ is the pre-exponential factor of delayed and prompt fluorescence component, respectively; $\tau_{DF(i)}$ and $\tau_{PF(j)}$ is the lifetime of delayed and prompt fluorescence component, respectively. The rate constants of radiative (k_r) and nonradiative (k_{nr}) decay and intersystem crossing (k_{ISC}) are given by equations:

$$k_r = \frac{\varphi_{PF}}{\tau_{PF}}, \tag{S5}$$

$$k_{ISC} = \frac{\varphi_{DF}}{\varphi\, \tau_{PF}}, \tag{S6}$$

$$k_{nr} = \frac{1}{\tau_{PF}} - (k_r + k_{ISC}). \tag{S7}$$

*w*here φ is PLQY ($\varphi_{DF} + \varphi_{PF}$). Further, the quantum yields for ISC and rISC were calculated as

$$\varphi_{ISC} = k_{ISC} \tau_{PF}, \tag{S8}$$

$$\varphi_{rISC} = \frac{1 - \varphi_{PF}/\varphi}{\varphi_{ISC}}. \tag{S9}$$

Finally, the rate constant of rISC (k_{rISC}) was calculated as

$$k_{rISC} = \frac{\varphi_{rISC}}{\tau_{DF}} \left(\frac{\varphi}{\varphi_{PF}}\right). \tag{S10}$$

[13] a) Dias, F. B.; Penfold, T. J.; Monkman, A. P.; Photophysics of Thermally Activated Delayed Fluorescence Molecules. *Methods Appl. Fluoresc.* **2017**, *5*, 012001; b) Tao, Y.; Yuan, K.; Chen, T.; Xu, P.; Li, H.; Chen, R.; Zheng, C.; Zhang L.; Huang, W. Thermally Activated Delayed Fluorescence Materials Towards the Breakthrough of Organoelectronics. *Adv. Mater*. **2014**, *26*, 7931-7958.

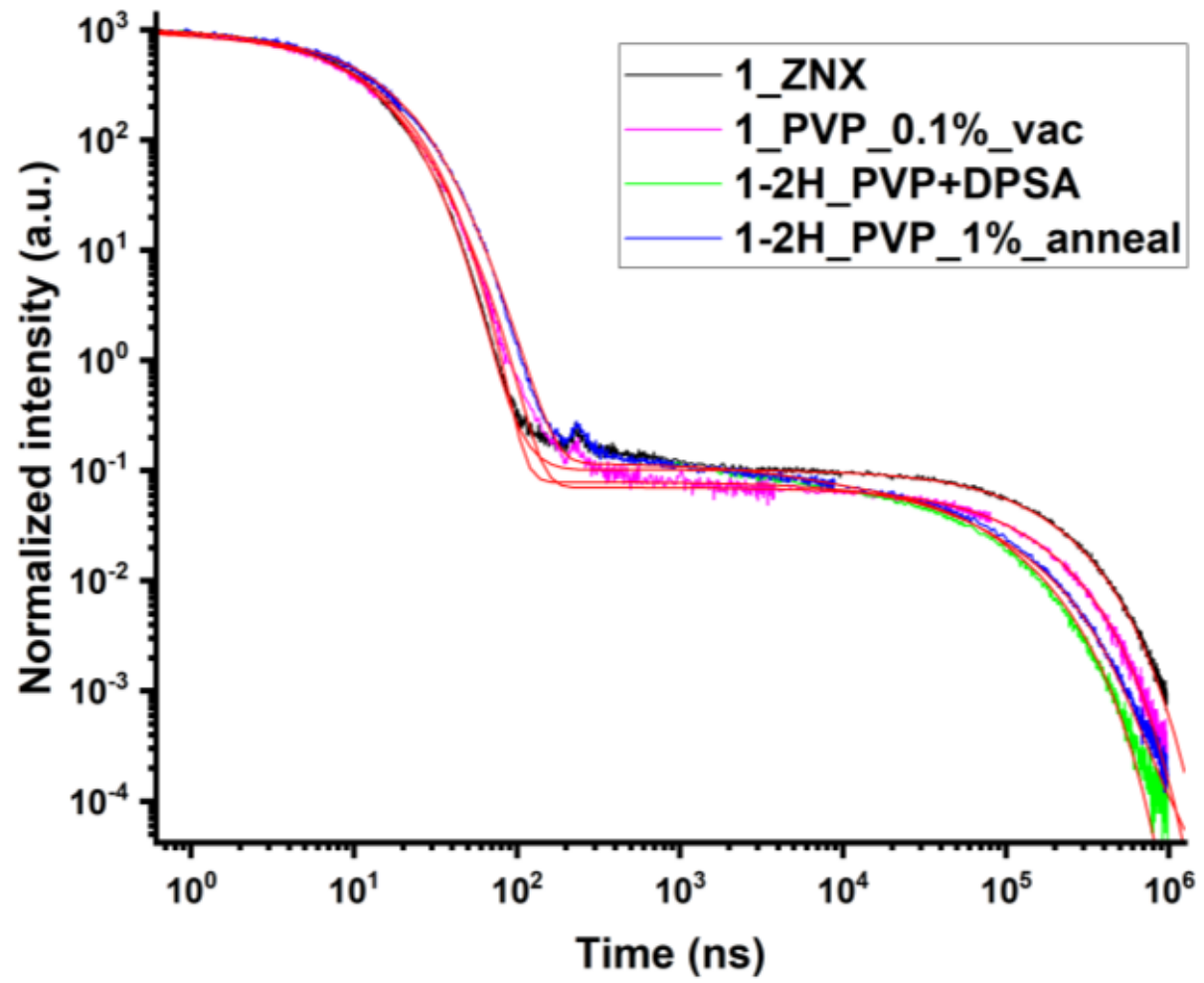

Figure S46. PL decay profiles of **1** and **1**-2H in ZNX and PVP films measured under vacuum at 293 K together with the corresponding multiexponential fits (red lines). Both time and PL intensity are presented on logarithmic scales to visualize the prompt and delayed emission regimes within the same decay profile.

Table S2. Multiexponential fitting parameters of the PL decays of **1** and **1**-2H in ZNX and PVP films at 293 K under vacuum.

Sample	Regime	A1	τ1	f1 [%]	A2	τ2	f2 [%]	A3	τ3	f3 [%]	R^2	χ_{red}^2
**1/ZNX**	PF	1042.9	9.76 ns	100							0.999	37
	DF	0.0296	96 μs	16	0.07203	207.5 μs	84					
**1-2H/ZNX (DPSA)**	PF	797.67	4.23 ns	54.3	297.64	9.55 ns	45.4	0.3668	51 ns	0.3	0.968	1916
	DF	0.005198	19 μs	33	0.001034	139 μs	48	8.83×10^{-5}	644 μs	19		
**1/PVP**	PF	851.19	9.00 ns	71.8	173.28	17.33 ns	28.2				0.998	88.1
	DF	0.02128	69 μs	15	0.04864	172 μs	85					
**1-2H/PVP annealed**	PF	111.81	0.35 ns	0.4	943.56	11.5 ns	99.6				0.989	574
	DF	0.03629	35.6 μs	18	0.04512	131 μs	82					
**1-2H/PVP (DPSA)**	PF	1008.21	11.1 ns	90.5	47.123	25 ns	9.5				0.999	73.7
	DF	0.03616	4.6 μs	2.58	0.04164	42.2 μs	26.5	0.03945	119.2 μs	71		

Table S3. Experimental and derived photophysical parameters used for the determination of the kinetic rate constants at 293 K.

**Sample**	**PLQY [%]**	**Φ_{DF}/Φ_{PF}**	**Φ_{PF} [%]**	**Φ_{DF} [%]**	**Φ_{ISC} [%]**	**τ_{PF} [ns]**	**τ_{DF} [µs]**	**k_r [10^7 s^{-1}]**	**k_{ISC} [10^7 s^{-1}]**	**k_{nr} [10^7 s^{-1}]**	**k_{rISC} [10^4 s^{-1}]**
**1**/ZNX	93.4	1.74	34.1	59	64	9.8	190	3.5	6.5	0.2	1.4
**1-2H**/ZNX (DPSA)	29	0.048	27.9	1	5	6.8	195	4.1	0.7	9.9	0.54
**1**/PVP	84	0.917	43.9	40	48	11.3	157	3.9	4.2	0.7	1.2
**1-2H**/PVP annealed	50	0.661	30.1	20	40	11.5	114	2.6	3.5	2.6	1.5
**1-2H**/PVP (DPSA)	47	0.529	30.7	16	35	12.4	95.8	2.5	2.8	2.8	1.6

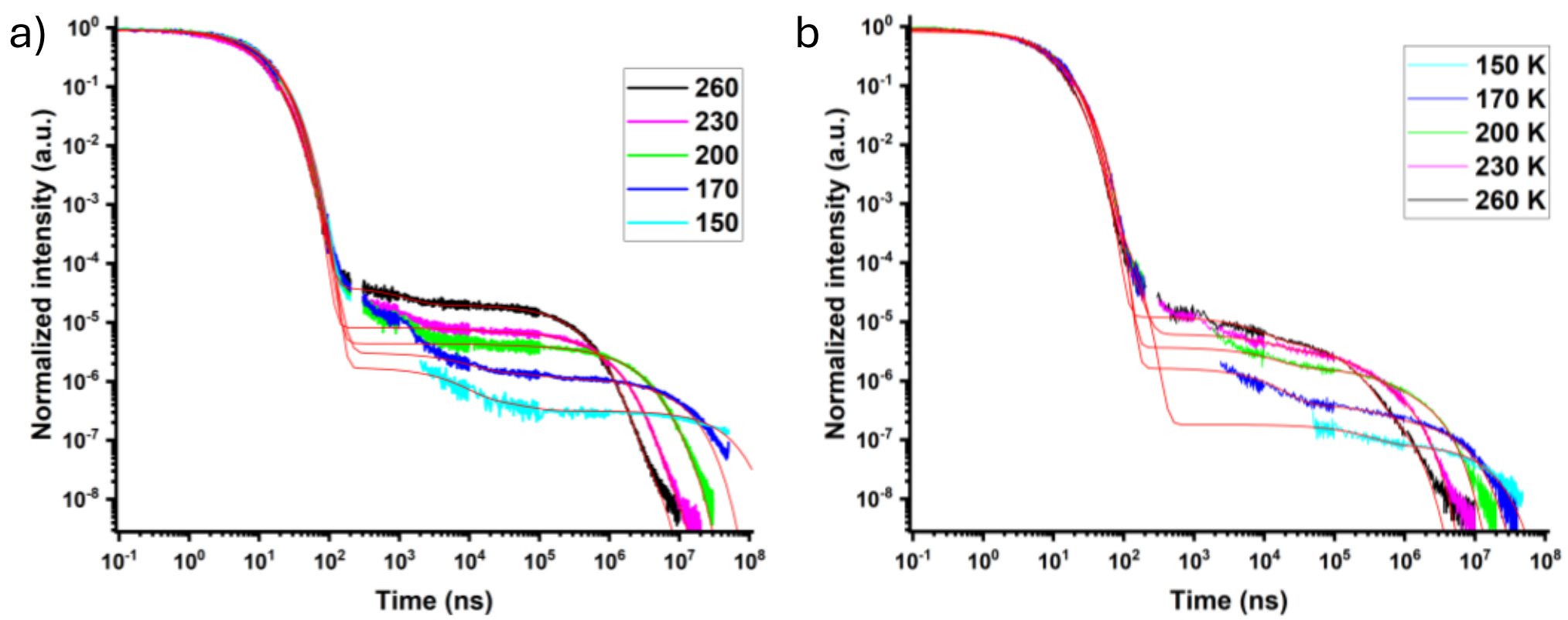

Figure S47. Temperature-dependent PL decay profiles and corresponding multiexponential fits (red lines) of (a) **1** (ZNX) and (b) **1**-2H (ZNX doped with DPSA). Both axes are shown on logarithmic scales.

Table S4. Multiexponential fitting parameters of the temperature-dependent PL decays of **1** in ZNX (see Figure S47, A).

**T [K]**		**A1**	**τ1**	**f1 [%]**	**A2**	**τ2**	**f2 [%]**	**A3**	**τ3 [µs]**	**f3 [%]**	**A4**	**τ4 [µs]**	f4 [%]	R2	χ_{red}^2
**260**	PF	0.83533	8 ns	82.9	0.08961	15 ns	17.1							0.99809	5.27×10^{-5}
	DF	2.209×10^{-5}	1 µs	0.3	9.931×10^{-6}	222.4 µs	30	9.682×10^{-6}	486.7	64	2.489×10^{-7}	1775	6		
**230**	PF	0.34419	5 ns	22.9	0.575	11 ns	77.1							0.99641	8.28×10^{-5}
	DF	1.159×10^{-6}	10.75 µs	0.2	3.266×10^{-6}	496.1 µs	26	3.659×10^{-6}	1124	66	1.503×10^{-7}	3317	8		
**200**	PF	0.87397	10 ns	89.9	0.06165	16 ns	10.1							0.99738	6.90×10^{-5}
	DF	2.954×10^{-7}	35.72 µs	0.1	4.595×10^{-7}	459.3 µs	2	2.953×10^{-7}	2251	63	6.774×10^{-7}	5452	35		
**170**	PF	0.89912	11 ns	98.2	0.007878	24 ns	1.8							0.99701	6.53×10^{-5}
	DF	1.585×10^{-6}	8.042 µs	0.11	3.548×10^{-7}	97.96 µs	0.3	1.862×10^{-7}	2489	4	9.529×10^{-7}	11670	96		
**150**	PF	0.55464	9 ns	47.2	0.39262	14 ns	52.8							0.9995	1.41×10^{-5}
	DF	1.180×10^{-6}	5.943 µs	0.13	2.312×10^{-7}	46.67 µs	0.2	1.991×10^{-7}	27097	100					

Table S5. Temperature-dependent photophysical parameters and derived kinetic rate constants of **1** in ZNX.
*PLQY was reconstructed from the ratio of the integrated areas under the PL decay curves measured at a given temperature and at 293 K.

Sample	PLQY* [%]	Φ_{DF}/Φ_{PF}	Φ_{PF} [%]	Φ_{DF} [%]	Φ_{ISC} [%]	τ_{PF} [ns]	τ_{DF} [µs]	k_r [10^7 s^{-1}]	k_{ISC} [10^7 s^{-1}]	k_{nr} [10^7 s^{-1}]	k_{rISC} [10^4 s^{-1}]
**293**	93.4	1.74	34.1	59	64	9.8	190	3.5	6.5	0.2	**1.44**
**260**	100	0.92	52.1	48	48	9.3	477	5.6	5.2	0	**0.40**
**230**	100	0.776	56.3	44	44	9.6	1124	5.8	4.5	0	**0.16**
**200**	100	1.086	47.9	52	52	10.6	3325	4.5	4.9	0	**0.063**
**170**	100	1.154	46.4	54	54	11	11267	4.2	4.9	0	**0.019**
**150**	100	0.516	66	34	34	11.4	27008	5.8	3	0	**0.0056**

Table S6. Multiexponential fitting parameters of the temperature-dependent PL decays of **1**-2H in ZNX (DPSA) (**see Figure S47, b**).

T [K]	Regime	A1	τ1	f1 [%]	A2	τ2	f2 [%]	A3	τ3	f3 [%]	R^2	χ_{red}^2
**293**	PF	0.73332	4.23 ns	54.3	0.27363	9.55 ns	45.4	3.372×10^{-4}	51 ns	0.3	0.99827	3.92×10^{-5}
	DF	4.779×10^{-6}	19 µs	33	9.502×10^{-7}	139 µs	48	8.118×10^{-8}	644 µs	19		
**260**	PF	0.59574	5.63 ns	46.8	0.33759	11.18 ns	53.2				0.99839	3.64×10^{-5}
	DF	7.383×10^{-6}	7 µs	5	4.344×10^{-6}	138 µs	58	5.624×10^{-7}	680 µs	37		
**230**	PF	0.83063	9.62 ns	99.2	0.001601	41.09 ns	0.8				0.99515	1.15×10^{-4}
	DF	2.816×10^{-6}	11.33 µs	2	2.133×10^{-6}	201.9 µs	27	1.304×10^{-6}	868.8 µs	71		
**200**	PF	0.57546	6.99 ns	45.9	0.34726	13.55 ns	54.1				0.99843	4.14×10^{-5}
	DF	1.993×10^{-6}	11.91 µs	1	8.073×10^{-7}	411.7 µs	14	9.065×10^{-7}	2226 µs	85		
**170**	PF	0.4377	6.22 ns	31.5	0.44765	13.09 ns	68.5				0.99678	6.44×10^{-5}
	DF	1.251×10^{-6}	12.42 µs	1	1.769×10^{-7}	439 µs	5	2.289×10^{-7}	6380 µs	94		
**150**	PF	0.8242	10.09 ns	98.8	0.002312	43.85 ns	1.2				0.99404	1.22×10^{-4}
	DF	9.798×10^{-8}	260.3 µs	3	7.423×10^{-8}	11110 µs	97					

Table S7. Temperature-dependent photophysical parameters and derived kinetic rate constants of **1**-2H in ZNX (DPSA).
*PLQY was reconstructed from the ratio of the integrated areas under the PL decay curves measured at a given temperature and at 293 K.

Sample	PLQY* [%]	Φ_{DF}/Φ_{PF}	Φ_{PF} [%]	Φ_{DF} [%]	Φ_{ISC} [%]	τ_{PF} [ns]	τ_{DF} [µs]	k_r [10^7 s^{-1}]	k_{ISC} [10^7 s^{-1}]	k_{nr} [10^7 s^{-1}]	k_{rISC} [10^4 s^{-1}]
**293**	29	0.048	27.9	1	5	6.8	195	4.1	0.7	9.9	**0.54**
**260**	44	0.145	38.2	6	13	8.6	334	4.5	1.5	5.7	**0.34**
**230**	34	0.198	28.8	6	17	9.9	673	2.9	1.7	5.5	**0.18**
**200**	34	0.272	26.5	7	21	10.5	1942	2.5	2.0	4.9	**0.066**
**170**	27	0.181	22.6	4	15	10.9	6023	2.1	1.4	5.7	**0.020**
**150**	26	0.101	24	2	9	10.5	10815	2.3	0.9	6.4	**0.010**

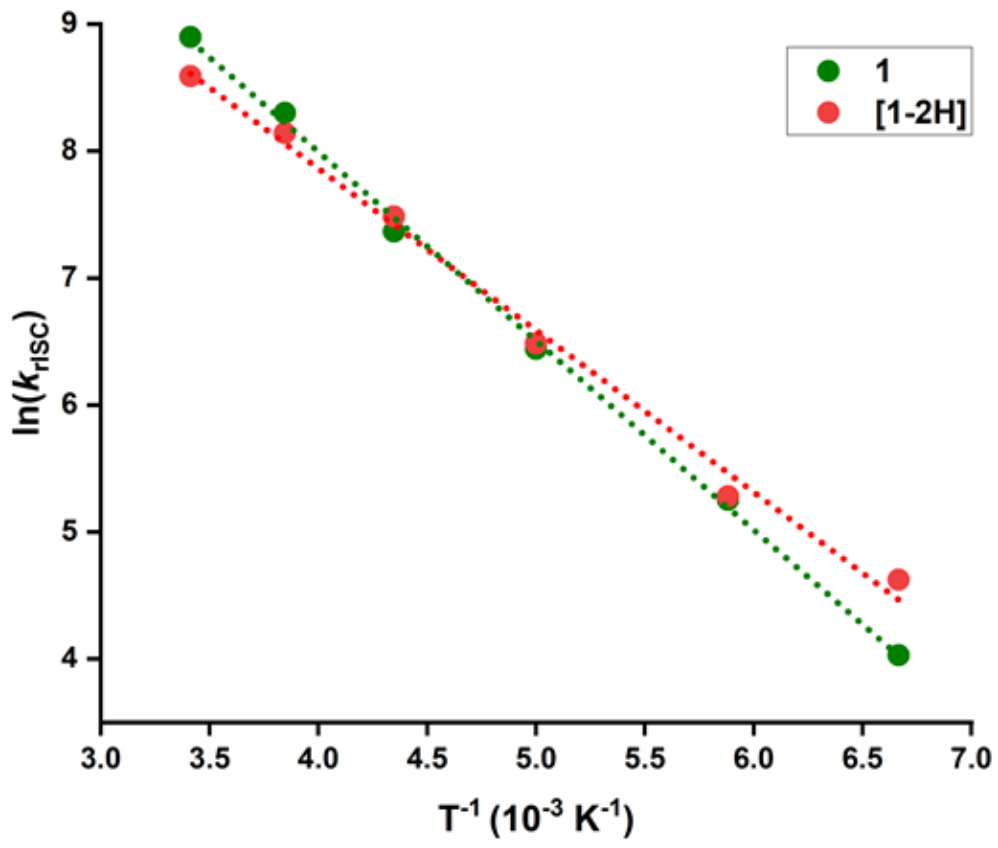

Figure S48. Arrhenius plot showing the temperature dependence of the rISC rate constant. Green symbols correspond to compound **1** in the ZNX film, while red symbols represent the protonated form **1-2H** obtained in a DPSA-doped ZNX film.

Table S8. Arrhenius parameters for *r*ISC (**1** measured in the ZNX film, **1**-2H measured in the DPSA-doped ZNX film, emitter conc. 0.1%, w/w, acid 0.3%, w/w.)

Sample	Intercept	Slope [K]	R^2	E_a [eV]
**1**	13.95 ± 0.15	-1489 ± 29	0.9984	0.128 ± 0.003
**1-2H**	12.96 ± 0.26	-1274 ± 51	0.9935	0.110 ± 0.004

8. Cyclic voltammograms

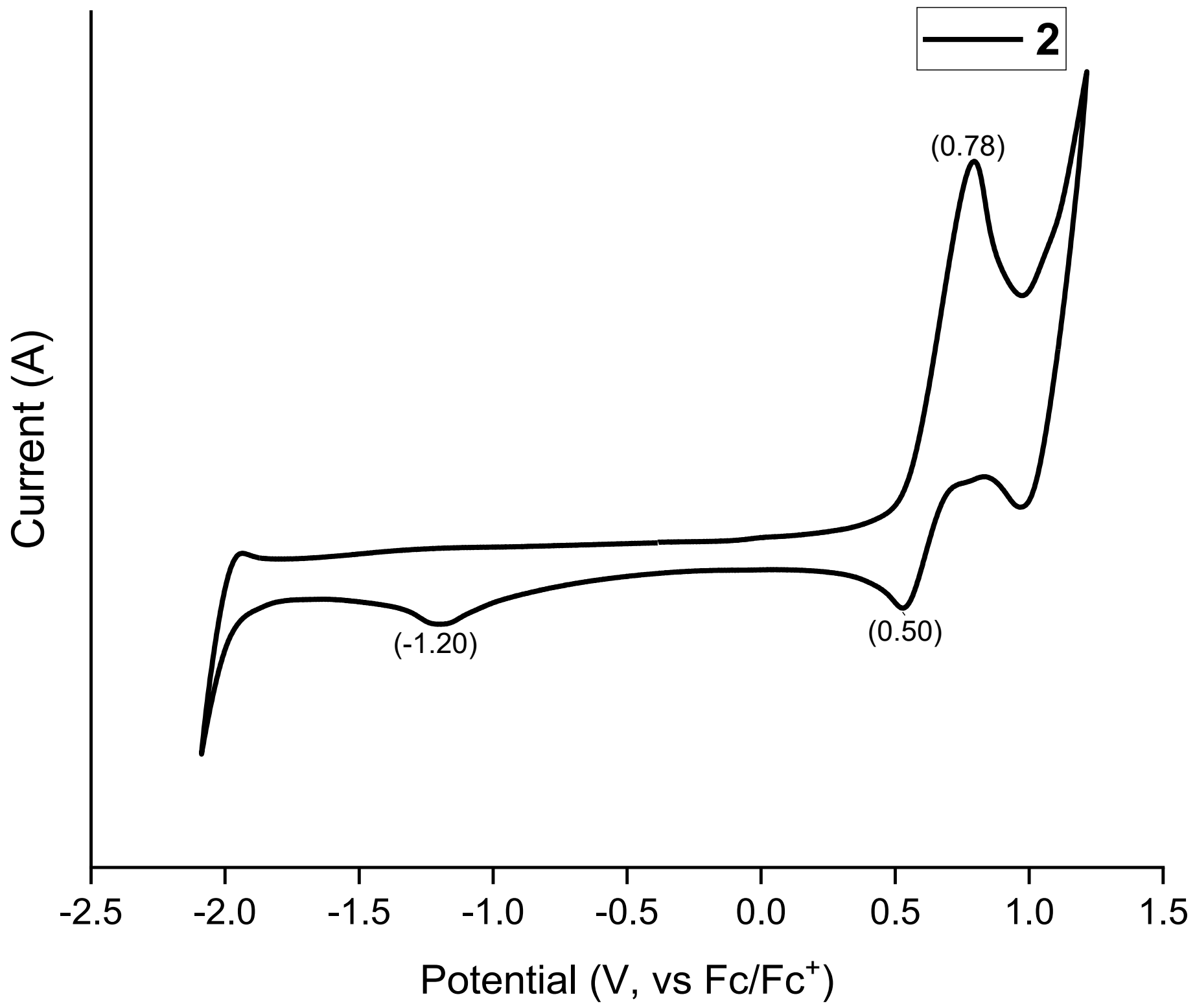

Figure S49. Cyclic voltammogram recorded for the oxidation waves of **2** in CH_2Cl_2, vs Fc/Fc^+ with n-Bu_4NPF_6 as electrolyte, ν = 100 mV/s.

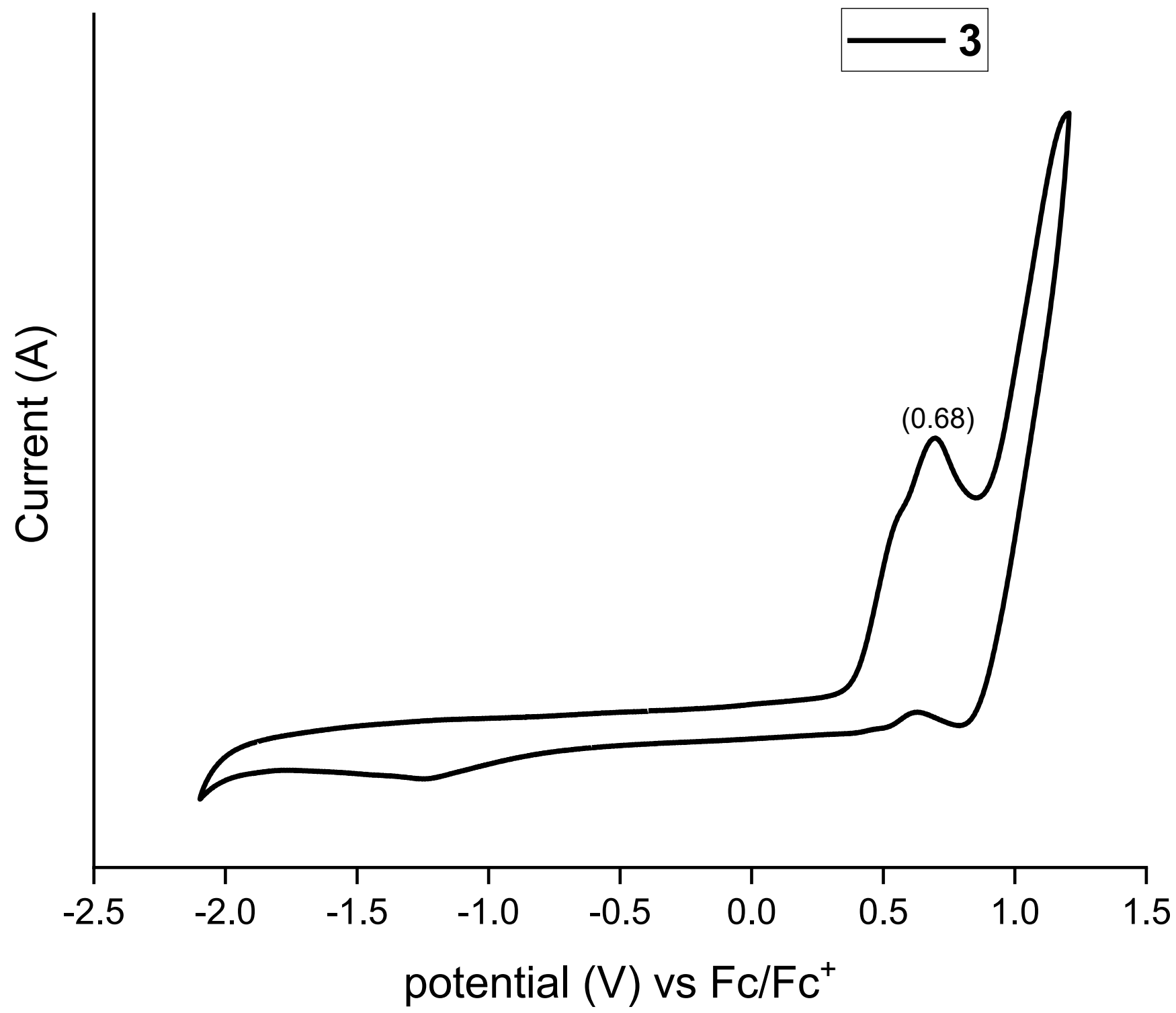

Figure S50. Cyclic voltammogram recorded for the oxidation waves of **3** in CH_2Cl_2, vs Fc/Fc^+ with n-Bu_4NPF_6 as electrolyte, ν = 100 mV/s.

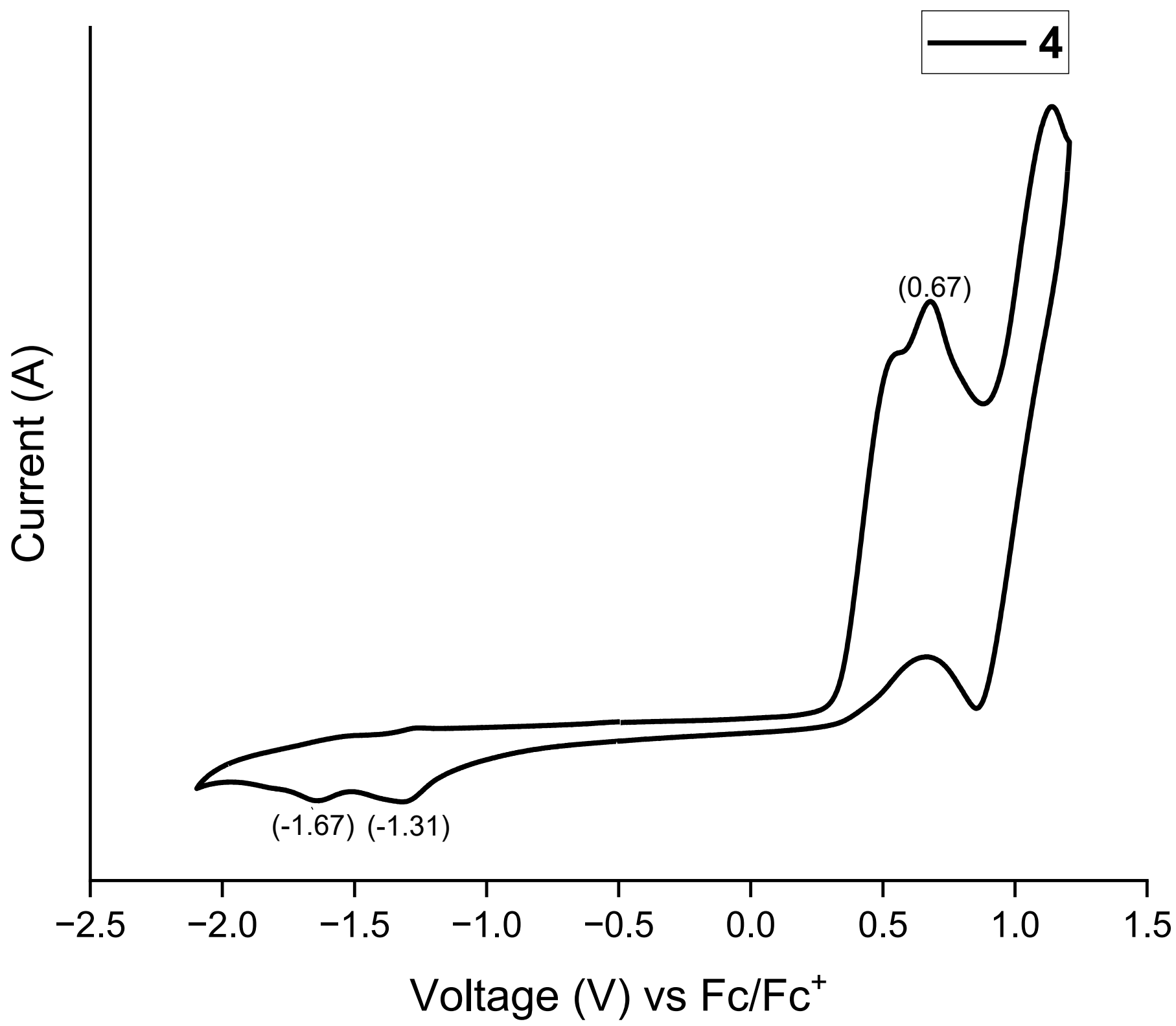

Figure S51. Cyclic voltammogram recorded for the oxidation waves of **4** in CH_2Cl_2, vs Fc/Fc^+ with n-Bu_4NPF_6 as electrolyte, ν = 100 mV/s.

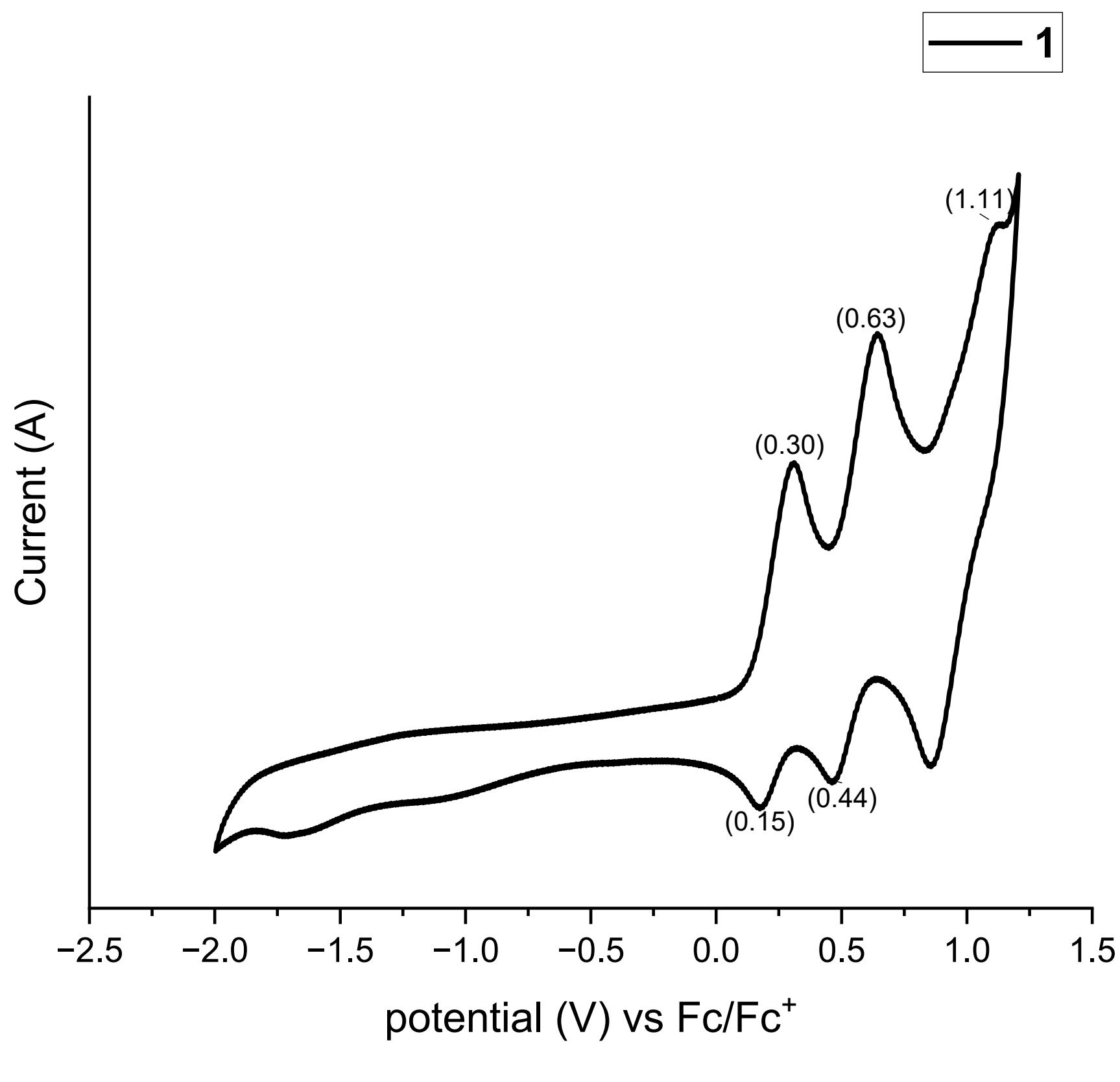

Figure S52. Cyclic voltammogram recorded for the oxidation waves of **1** in CH_2Cl_2, vs Fc/Fc^+ with *n*-Bu_4NPF_6 as electrolyte, ν = 100 mV/s.

9. ESI-MS spectra

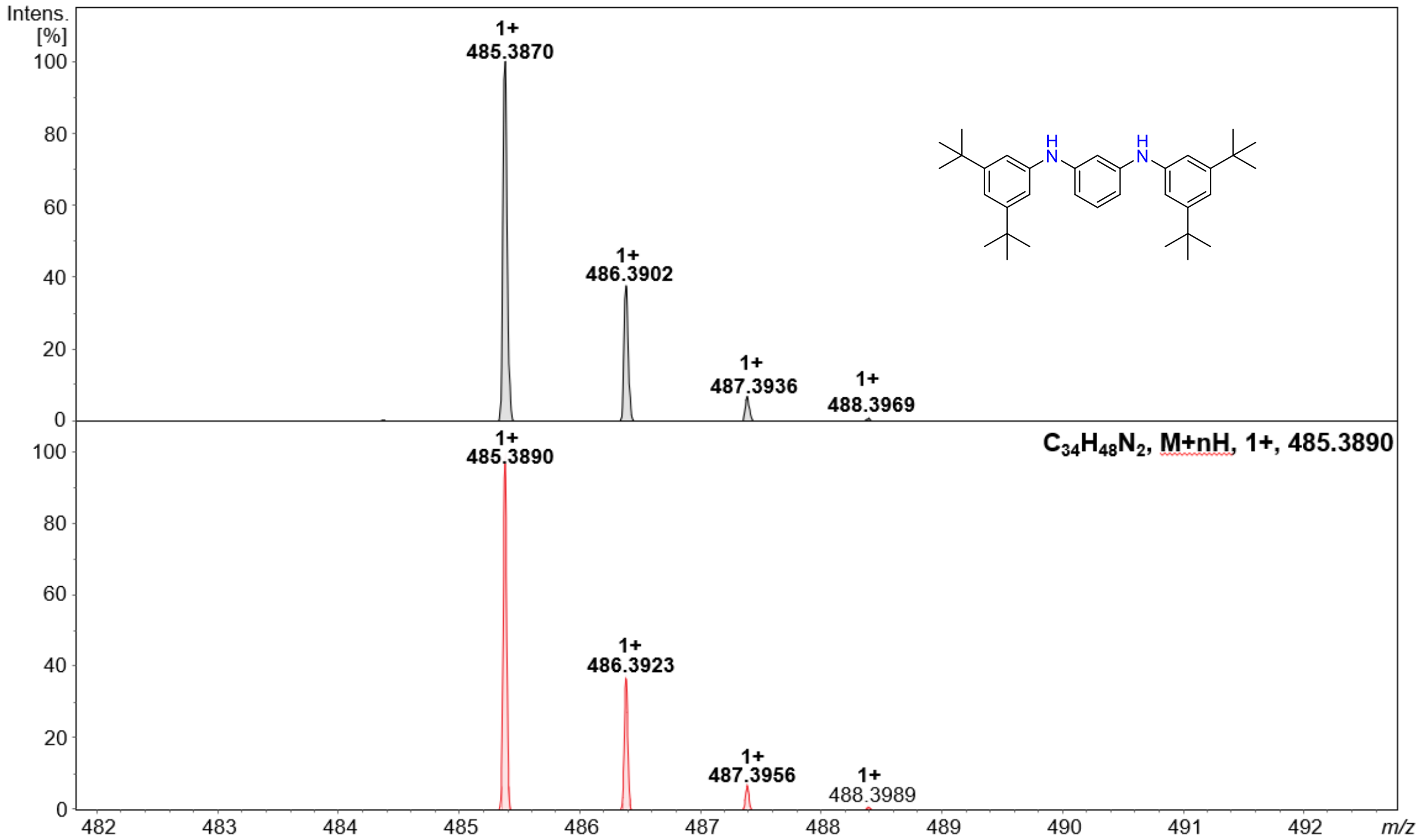

Figure S53. ESI-MS spectrum of **S1** with simulated spectrum (bottom panel - simulated isotopic patterns for molecular formula of the investigated compounds).

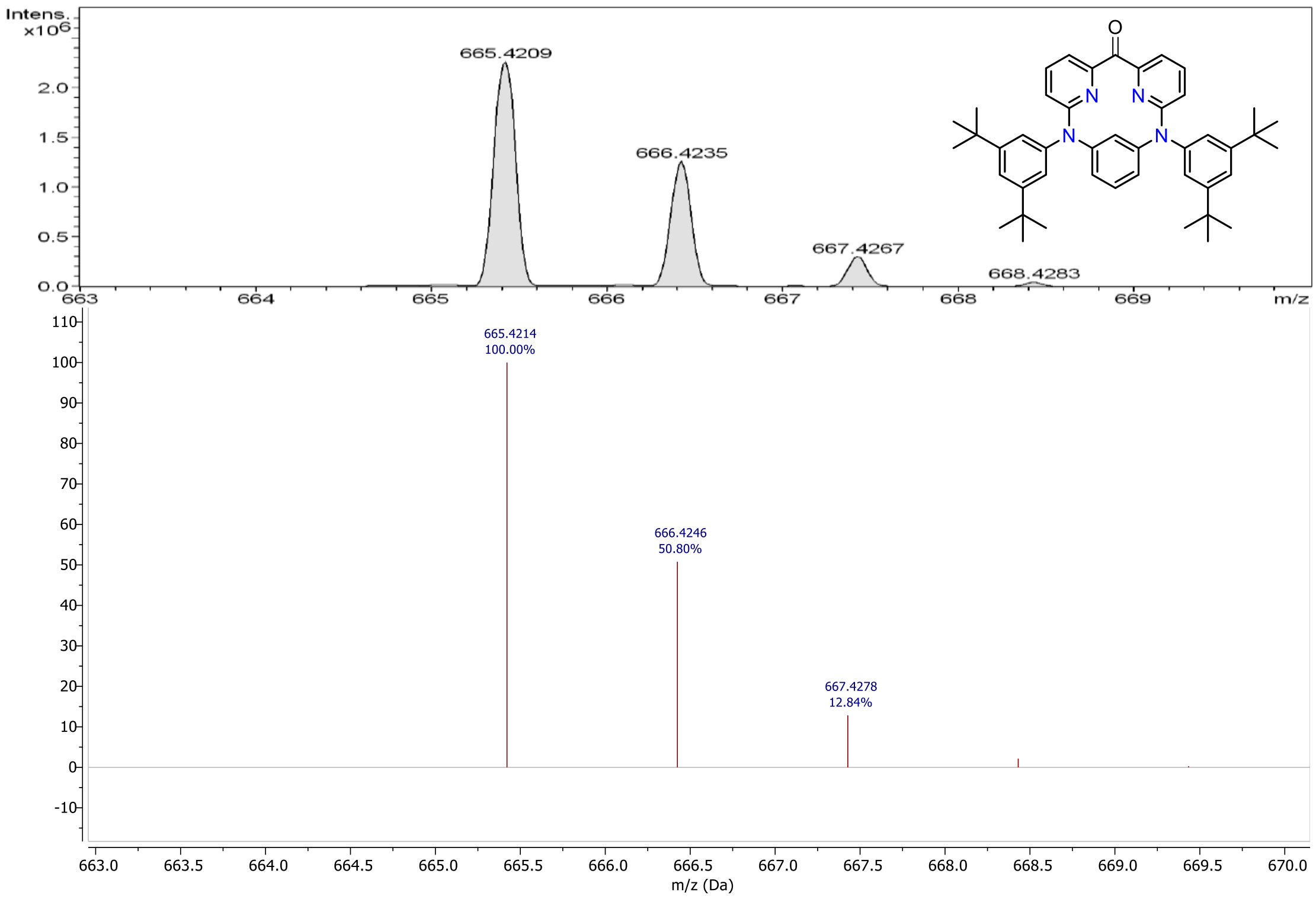

Figure S54. ESI-MS spectrum of **2** with simulated spectrum (bottom panel - simulated isotopic patterns for molecular formula of investigated compounds).

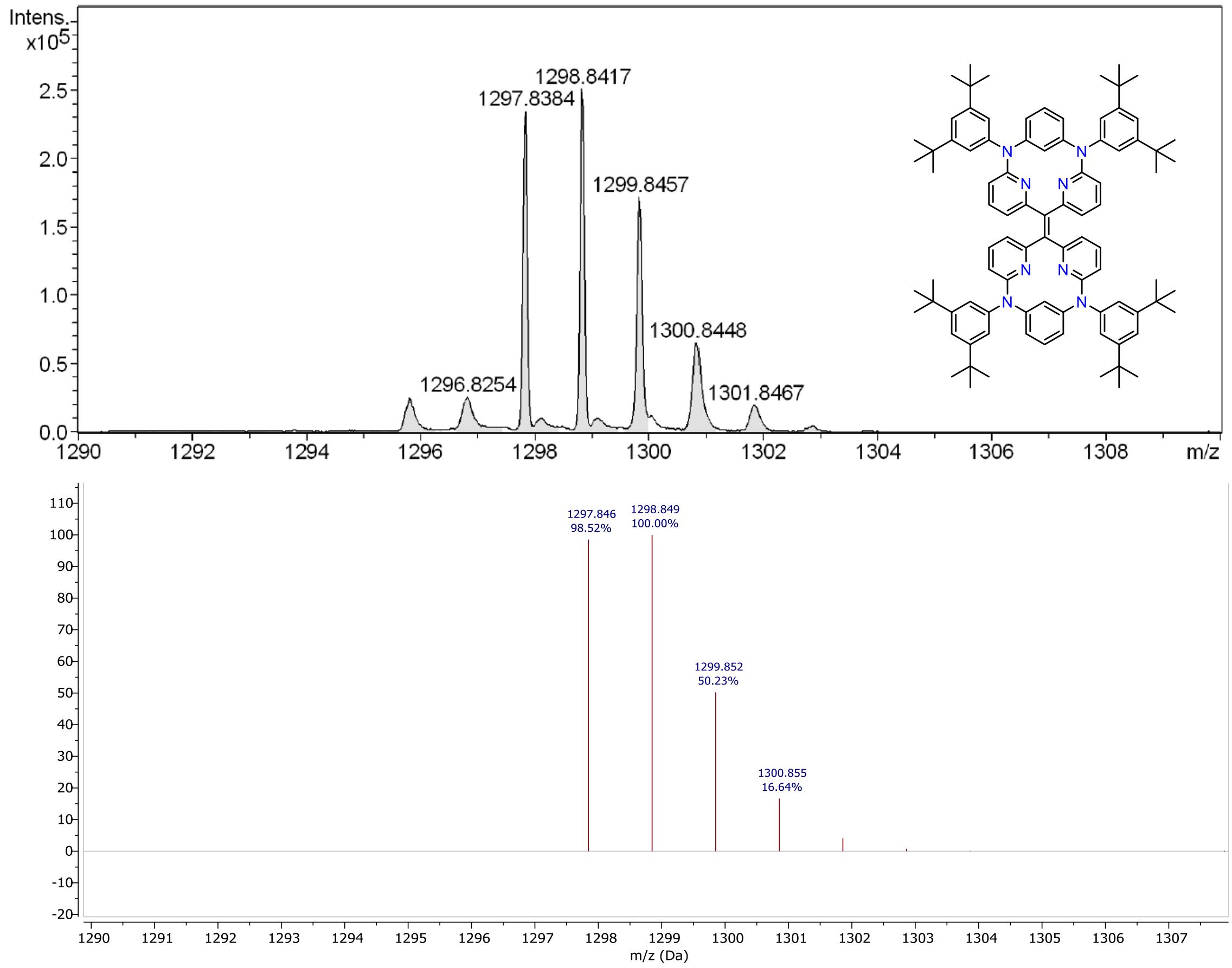

Figure S55. ESI-MS spectrum of **3** with simulated spectrum (bottom panel - simulated isotopic patterns for molecular formula of investigated compounds).

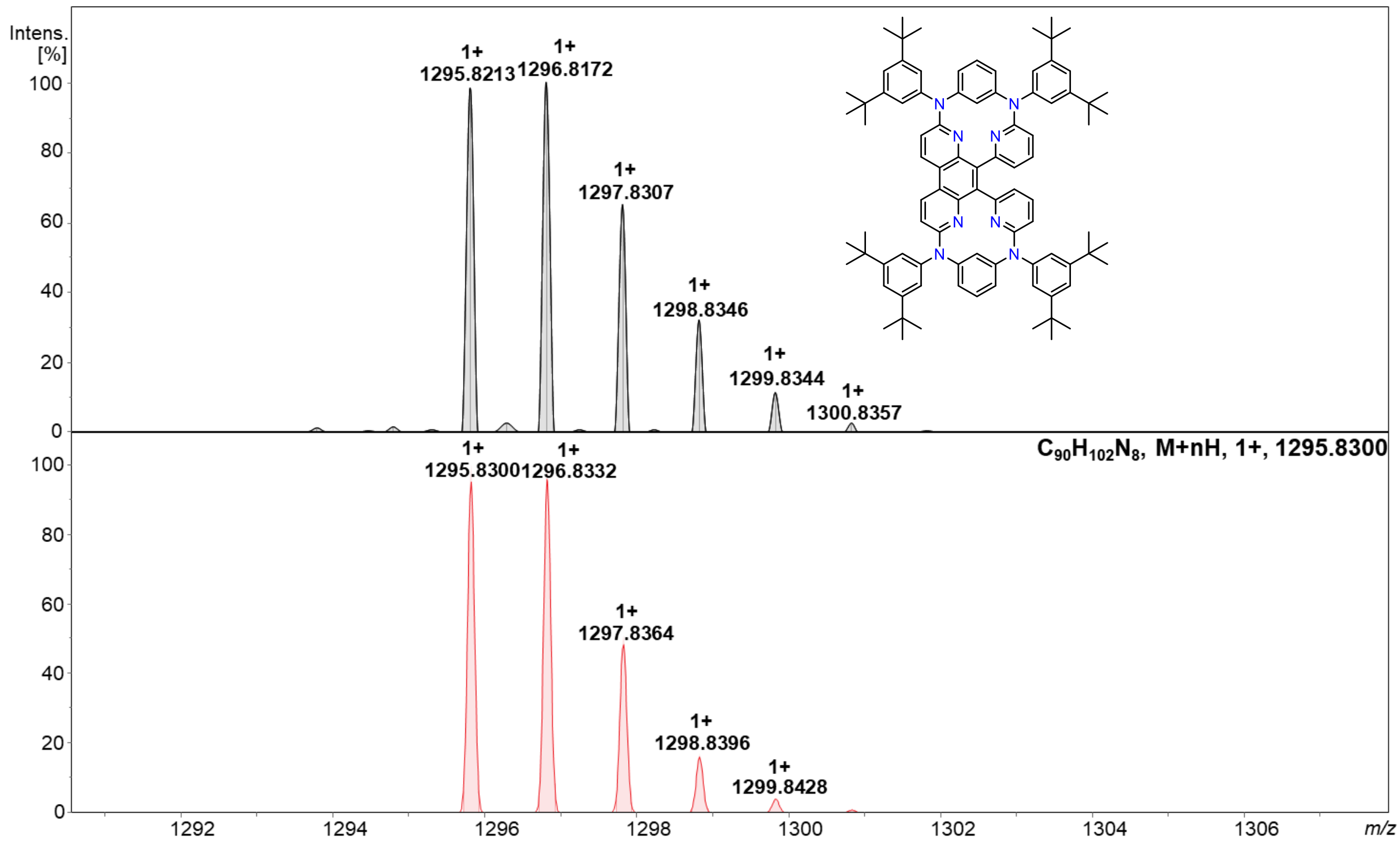

Figure S56. ESI-MS spectrum of **4** with simulated spectrum (bottom panel - simulated isotopic patterns for molecular formula of investigated compounds).

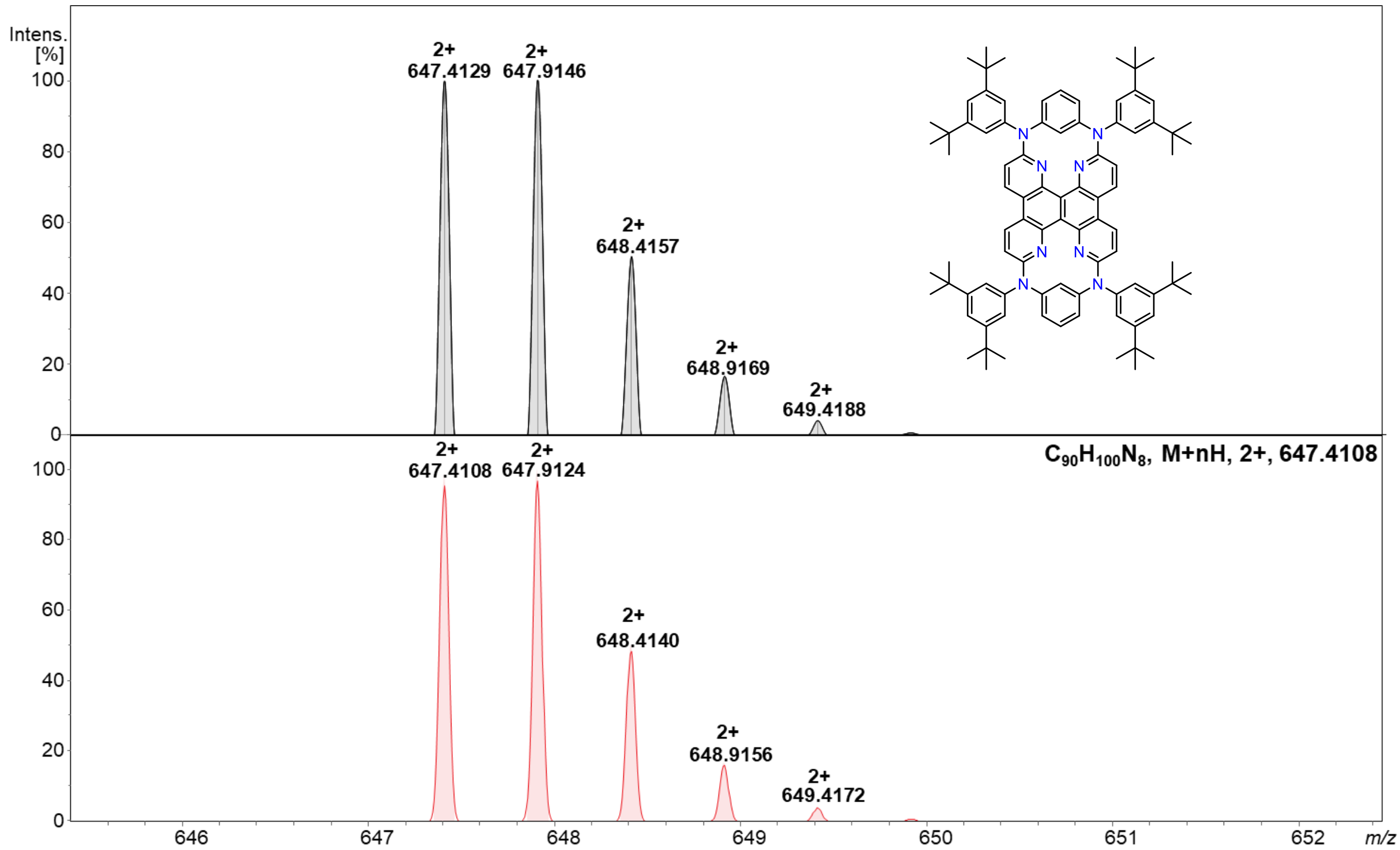

Figure S57. ESI-MS spectrum of **1** with simulated spectrum (bottom panel - simulated isotopic patterns for molecular formula of investigated compounds).

10. Theoretical calculations

10.1 NICS calculations

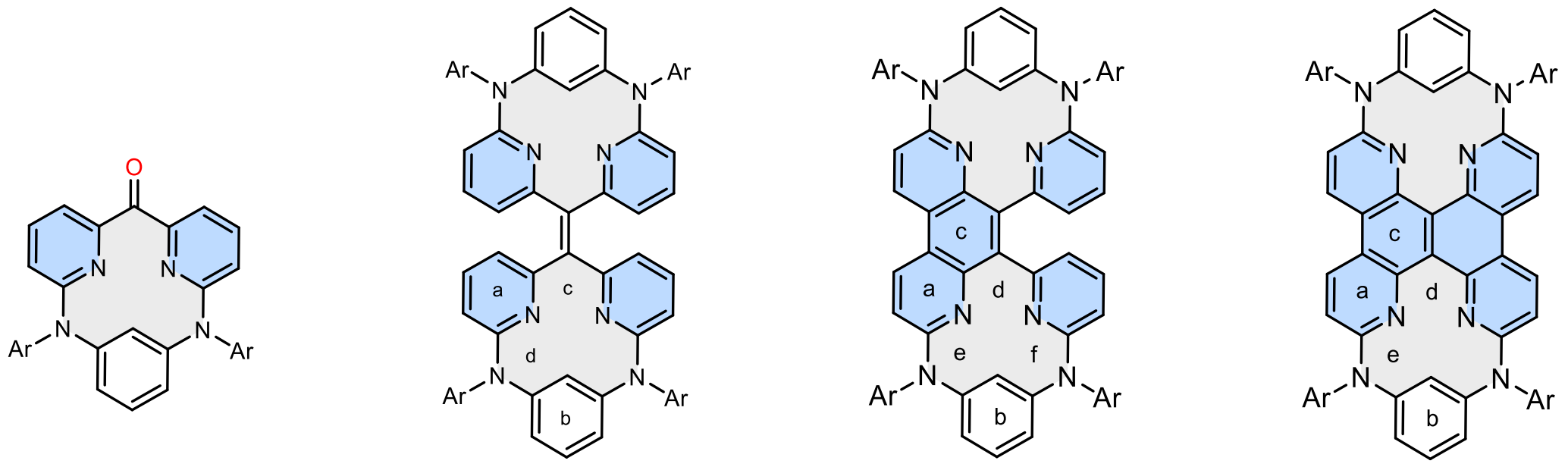

	2			3			4			1		
**NICS**	$(0)_{zz}$	$(1)_{zz}$	$(-1)_{zz}$	$(0)_{zz}$	$(1)_{zz}$	$(-1)_{zz}$	$(0)_{zz}$	$(1)_{zz}$	$(-1)_{zz}$	$(0)_{zz}$	$(1)_{zz}$	$(-1)_{zz}$
**a**	-1.93	-13.49	-15.39	-1.67	-7.00	-9.09	-0.26	-20.13	-18.24	1.90	-13.98	-15.37
**b**	-3.74	-3.18	-5.00	-1.82	0.02	-2.31	-3.32	-7.14	-5.74	-1.63	-5.25	-7.01
**c**	23.96	10.15	7.57	20.84	5.34	7.27	4.56	-13.14	-13.11	7.27	-8.69	-11.58
**d**	21.62	6.92	8.67	19.92	7.96	6.76	28.58	9.83	13.3	35.22	12.95	11.88
**e**	-	-	-	-	-	-	23.95	10.06	8.16	26.32	8.77	10.03
**f**	-	-	-	-	-	-	23.07	7.70	10.23	-	-	-

Table S9. Summary of NICS value calculated at b3lyp/6-31g(d,p) degree of theory for **2, 3, 4 & 1.**

10.2 GIAO calculations

Table S10. Summary of NMR spectra (SCF GIAO method) calculated at b3lyp/6-31g(d,p) Empirical Dispersion =GD3BJ degree of theory for **2, 3, 4** and **1** and compared with experimental values using $CDCl_3$ (for **2, 3 & 1**) and CD_2Cl_2 (for **4**) as solvent.

	2		3		4		1	
	δ (ppm)		δ (ppm)		δ (ppm)		δ (ppm)	
	**Obs.**	**Calc.**	**Obs.**	**Calc.**	**Obs.**	**Calc.**	**Obs.**	**Calc.**
1	7.52	7.62	6.78	6.71	6.90	6.93	8.32	8.39
2	7.50	7.57	7.11	7.25	7.35	7.46	6.78	7.06
3	6.68	7.00	6.41	6.81	6.74	6.94	10.01	9.75
4	8.59	8.42	7.67	7.49	8.54	8.60	6.24	6.28
5	6.37	6.38	6.46	6.61	6.75	6.86	6.89	7.01
6	6.95	7.06	6.91	7.15	7.00	7.06	-	-
7	-	-	-	-	6.53	6.33	-	-
8	-	-	-	-	6.92	7.03	-	-
9	-	-	-	-	8.38	8.38	-	-

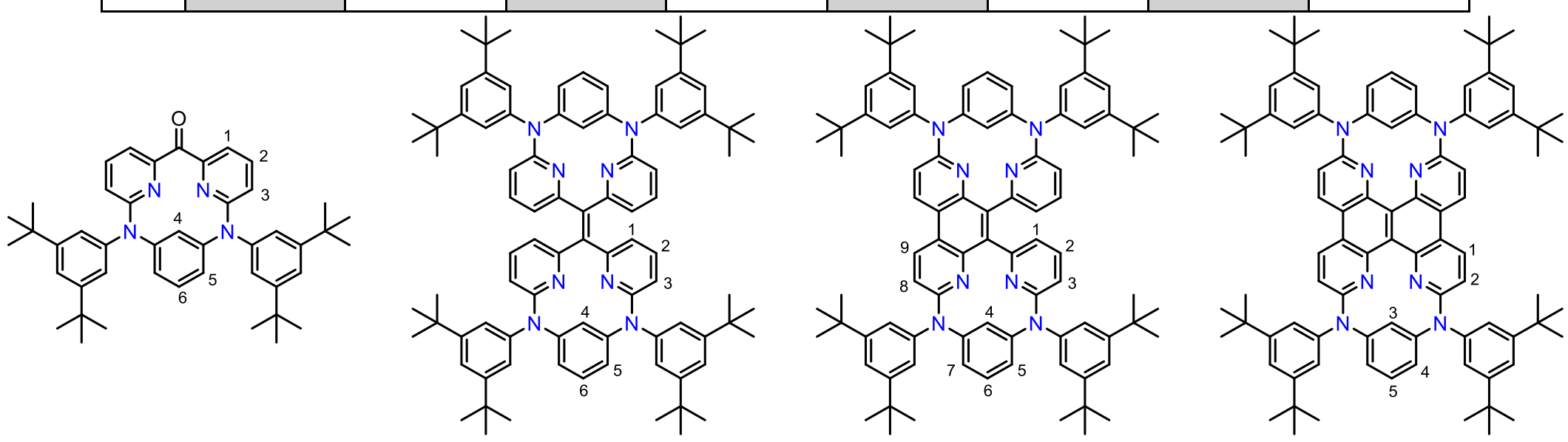

10.3 RMSD

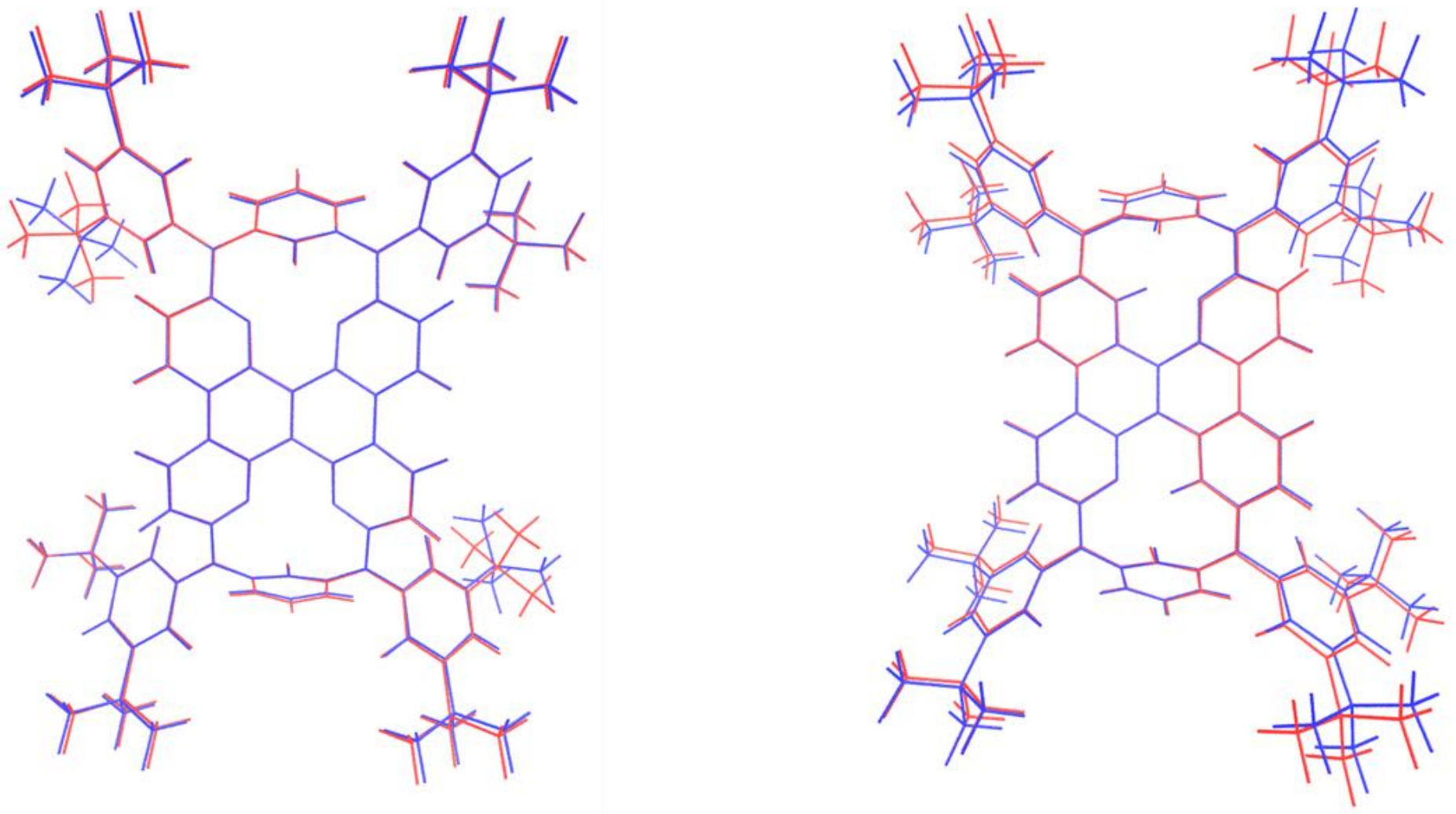

1
S_0-S_1
RMSD = 0.155 Å

[1+2eq TFA]
S_0-S_1
RMSD = 0.609 Å

Figure S58. Comparison of optimized structures of **1 (left),** [**1+2eq TFA] (right),** at S_0 (blue) and S_1 (red) states. DFT calculations at b3lyp/6-31g(d,p) Empirical Dispersion =GD3BJ degree of theory level in the gas phase.

10.4 Reorganizational Energy

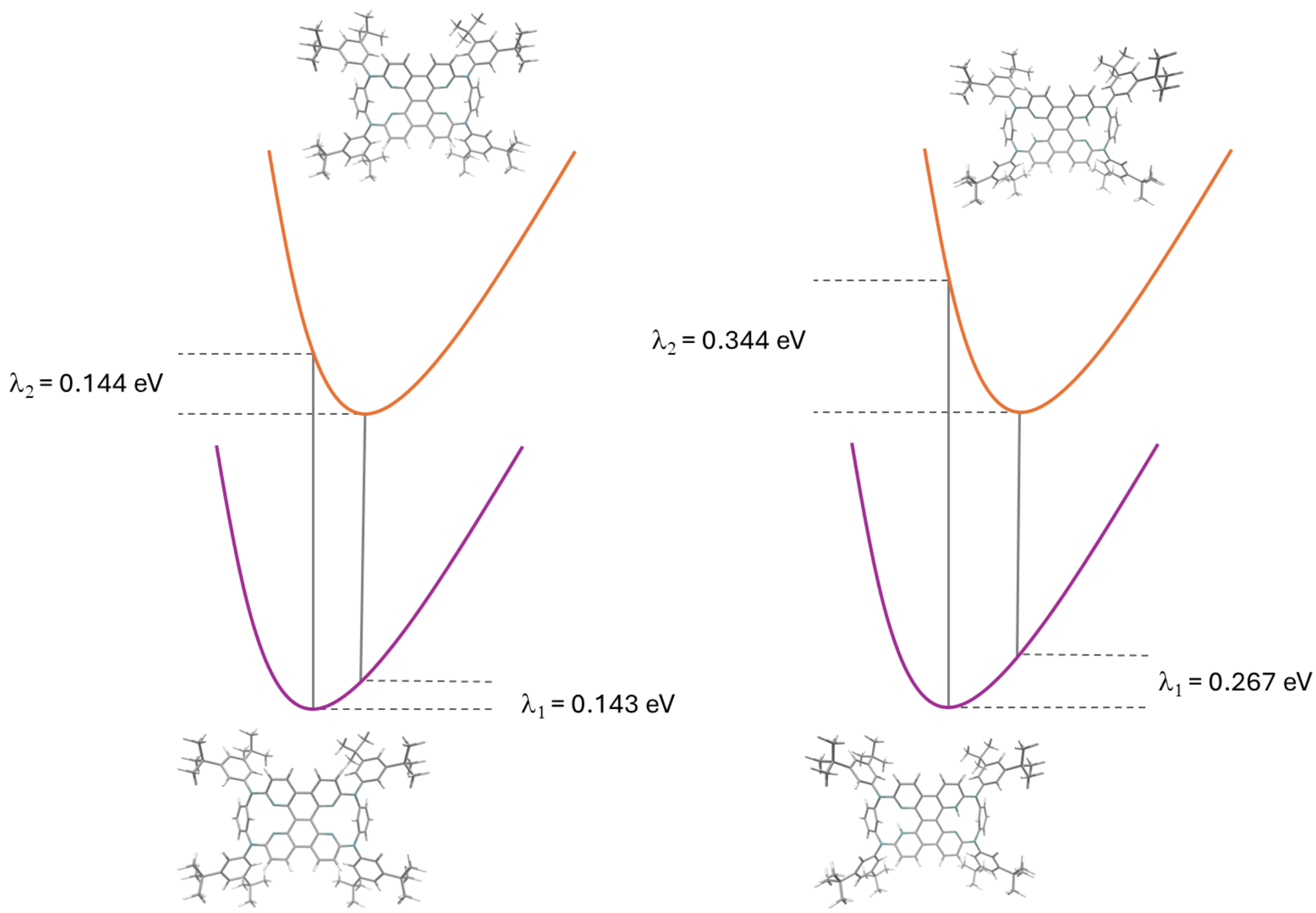

Figure S59. Optimized S_0 and S_1 geometries of **1 (left),** [**1+2eq TFA] (right),** and reorganization energy (λ_1 = S_0 to S_1 and λ_2 = S_0 to S_1). DFT calculations at b3lyp/6-31g(d,p) Empirical Dispersion = GD3BJ degree of theory level in the gas phase.

11. Cartesian Coordinates.

Cartesian coordinates for all geometries provided as pdb files attached to SI.

12. X-Ray Analysis

Table S11. Crystal data and structure refinement for **2, 3, 4** and **1**.

**Structure No.**	**2**	**3**	**4**	**1**
**CCDC**	**2540680**	**2540681**	**2533581**	**2540682**
Empirical formula	$C_{45}H_{52}N_4O$	$C_{90}H_{104}N_8$	$C_{90}H_{102}N_8$	$C_{90}H_{100}N_8$
Formula weight	664.90	1297.88	1295.86	1293.85
Temperature/K	100(2)	100(2)	100.15	100.15
Crystal system	Triclinic	Triclinic	Triclinic	Orthorhombic
Space group	P-1	P-1	P-1	P*bca*
a/Å	14.4874(8)	9.2558(8)	11.4225(11)	32.8329(4)
b/Å	16.2673(9)	11.3647(10)	19.3782(12)	22.7634(3)
c/Å	17.7398(10)	20.1855(16)	22.9979(17)	44.8059(5)
α/°	74.142(2)	104.382(3)	68.061(6)	90
β/°	78.907(2)	94.671(3)	83.592(7)	90
γ/°	86.439(2)	105.542(3)	84.917(6)	90
Volume/$Å^3$	3946.3(4)	1956.5(3)	4686.3(7)	33487.5(7)
Z	4	1	2	8
ρ_{calc} [g/cm^3]	1.119	1.102	1.097	0.692
μ/mm^{-1}	0.067	0.064	0.484	0.356
F(000)	1432.0	700.4	1670.0	7371.0
Radiation	MoKα (λ = 0.71073)	MoKα (λ = 0.71073)	CuKα (λ = 1.54184)	CuKα (λ = 1.54184)
2Θ range [°]	5.124 to 52.74	4.62 to 52.74	2.461 to 67.072	3.944 to 152.23
hkl ranges	-13 ≤ h ≤ 18, -19 ≤ k ≤ 20, -16 ≤ l ≤ 22	-11 ≤ h ≤ 11, -14 ≤ k ≤ 14, -25 ≤ l ≤ 25	-13 ≤ h ≤ 13, -23 ≤ k ≤ 23, -19 ≤ l ≤ 27	-33 ≤ h ≤ 41, -28 ≤ k ≤ 28, -52 ≤ l ≤ 56
Reflections collected/independent	18090/14019	40410/7990	56298/16559	234985/34515
restraints/parameters	30/987	0/443	161/952	0/2045
GooF on F2	1.083	1.056	0.953	1.053
R [I>=2σ (I)]/[all data]	R_1 = 0.1012/0.1465 wR_2 = 0.2117/0.2342	R_1 = 0.0983/0.1415 wR_2 = 0.2031/0.2253	R_1 = 0.1251/ 0.3251 wR_2 = 0.2612/ 0.4157	R_1 = 0.0945/ 0.1104 wR_2 = 0.2737/ 0.2889
Largest diff. peak/hole /e Å^{-3}	0.88/-0.45	1.51/-0.72	0.480/-0.279	3.09/-1.47

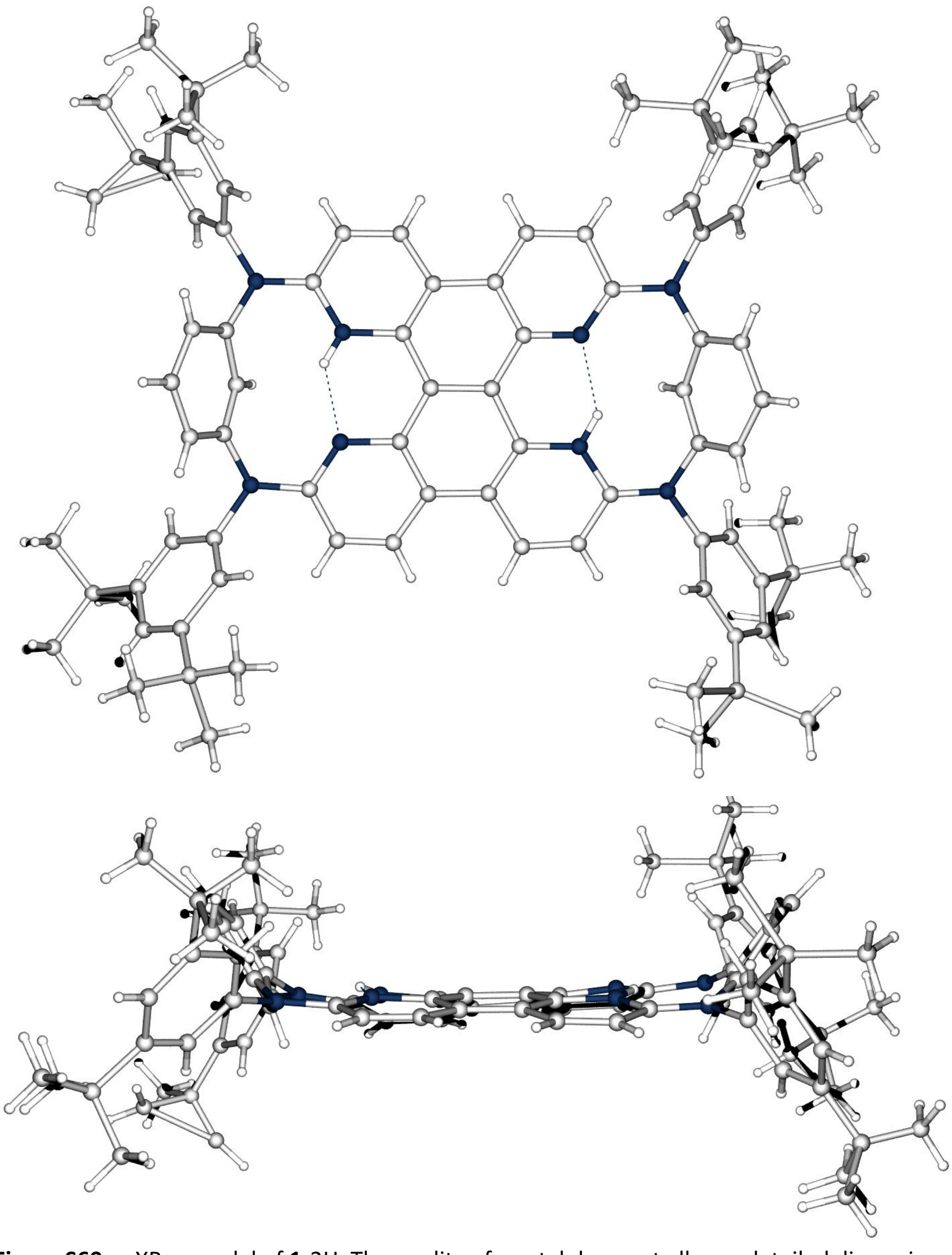

Figure S60. scXRay model of **1**-2H. The quality of crystal does not allow a detailed discussion.
